# PET Imaging of PARP Expression Using ^18^F-Olaparib

**DOI:** 10.2967/jnumed.118.213223

**Published:** 2019-04

**Authors:** Thomas C. Wilson, Mary-Ann Xavier, James Knight, Stefan Verhoog, Julia Baguña Torres, Michael Mosley, Samantha L. Hopkins, Sheena Wallington, Phillip D. Allen, Veerle Kersemans, Rebekka Hueting, Sean Smart, Véronique Gouverneur, Bart Cornelissen

**Affiliations:** 1 Department of Chemistry, University of Oxford, Oxford, United Kingdom; and; 2 CRUK/MRC Oxford Institute for Radiation Oncology, Department of Oncology, University of Oxford, Oxford, United Kingdom

**Keywords:** PET, olaparib, PARP, cancer, molecular imaging

## Abstract

Poly(ADP-ribose) polymerase (PARP) inhibitors are increasingly being studied as cancer drugs, as single agents, or as a part of combination therapies. Imaging of PARP using a radiolabeled inhibitor has been proposed for patient selection, outcome prediction, dose optimization, genotoxic therapy evaluation, and target engagement imaging of novel PARP-targeting agents. **Methods:** Here, via the copper-mediated ^18^F-radiofluorination of aryl boronic esters, we accessed, for the first time (to our knowledge), the ^18^F-radiolabeled isotopolog of the Food and Drug Administration–approved PARP inhibitor olaparib. The use of the ^18^F-labeled equivalent of olaparib allows direct prediction of the distribution of olaparib, given its exact structural likeness to the native, nonradiolabeled drug. **Results:** ^18^F-olaparib was taken up selectively in vitro in PARP-1–expressing cells. Irradiation increased PARP-1 expression and ^18^F-olaparib uptake in a radiation-dose–dependent fashion. PET imaging in mice showed specific uptake of ^18^F-olaparib in tumors expressing PARP-1 (3.2% ± 0.36% of the injected dose per gram of tissue in PSN-1 xenografts), correlating linearly with PARP-1 expression. Two hours after irradiation of the tumor (10 Gy), uptake of ^18^F-olaparib increased by 70% (*P* = 0.025). **Conclusion:** Taken together, we show that ^18^F-olaparib has great potential for noninvasive tumor imaging and monitoring of radiation damage.

See an invited perspective on this article on page 502.

Genomic instability in tumor tissue results from oncogenic and replicative stress, exogenous genotoxic insults, and tumor-specific DNA repair defects (1). Manipulating this genomic instability provides numerous therapeutic opportunities, and inhibitors of DNA damage repair enzymes have been explored as anticancer drugs (2). These include inhibitors of poly(ADP-ribose) polymerase (PARP). PARP enzymes are part of a 17-member subfamily of enzymes with similar function. PARP-1–PARP-3 sense DNA damage by binding to nicked DNA via their zinc-finger domains and play important roles in base excision repair, with PARP-1 being the most studied. PARP inhibitors reduce the enzymes’ catalytic activity (formation of poly-ADP-ribose chains from nicotinamide adenine dinucleotide) by binding to their nicotinamide adenine dinucleotide binding pocket and interfere with the ability of the PARP-enzyme-inhibitor complex to dissociate from damaged DNA (3,4). PARP inhibitors have been extensively studied as single agents and as radiation sensitizers and are especially effective in tumors with BRCA mutations (5). Radiation sensitization using PARP inhibition is known to act via several mechanisms, including straightforward inhibition of DNA repair and synthetic lethality. Further mechanisms include inhibition of chromatin remodeling, vasodilatory effects, decreased hypoxia, contextual lethality in hypoxic cells, replication-dependent radiation sensitization, G2/M arrest leading to time cooperation (6), and a possible role for reduction of Treg cells (7). The first clinically approved and most studied PARP inhibitor is olaparib (ku-0059436, AZ2281, Lynparza [AstraZeneca]). It inhibits the catalytic activity of PARP isoforms 1 and 2, and, albeit to a lesser extent, PARP-3. At present, over 100 clinical trials are ongoing using olaparib as a single drug or in combination with chemotherapy, immunotherapy, or radiation therapies. Other PARP inhibitors in clinical use or clinical trial include the recently approved rucaparib (Clovis Oncology), niraparib (Tesaro Inc.), talazoparib (Pfizer), as well as veliparib (AbbVie Inc.) (8).

Resistance to PARP inhibition, however, is common. It has been reported that 30%–70% of patients with mutations in DNA damage repair machinery do not respond to therapies including PARP inhibitors (9). Apart from some molecular mechanisms (10), resistance is often due to low PARP enzyme expression or to the inability of the drug to penetrate tumor tissue or part of the tumor tissue, because of increased interstitial pressure and desmoplasia—especially relevant in pancreatic adenocarcinomas—or an intact blood–brain barrier, in the case of brain tumors or brain metastases. Increased expression of drug efflux pumps may also prevent drug uptake in the tumor, most relevant for gastrointestinal and pancreatic tumors (11).

Recently, several reports have suggested that accurately measuring and monitoring PARP expression in vivo provides critical information on disease prognosis (12), as PARP expression has been found to independently correlate with worse outcomes in breast, ovarian, and other tumors (13,14). Assessment of DNA damage repair signaling activation may also contribute to genotoxic treatment evaluation, after chemo- or radiotherapy. To date, PARP expression and BRCA-ness status in tumors can be determined by immunohistochemistry or genetic sequencing on biopsy samples. However, many tumors are known to be extremely heterogeneous—because of their increased genomic instability—yet this heterogeneity is overlooked when sampling tissue from a single biopsy site. Furthermore, acquisition of reliable and high-quality biopsies is a significantly invasive and nontrivial procedure in many disease sites, such as lung, brain, or pancreas.

Given these challenges, scientists have sought to use alternative methods to measure PARP expression in vivo, especially PARP-1. Of those molecular imaging techniques available, PET has been shown to be ideal. PET allows for noninvasive, whole-body, repeatable visualization of olaparib delivery and its binding to PARP-1 to PARP-3 (15). In recent years, molecules structurally related to olaparib labeled with positron-emitting radionuclides such as ^18^F or ^123/131^I have been investigated for this purpose (15,16). In 2011, Reiner et al., via an inverse electron demand Diels-Alder reaction, reported access to ^18^F-BO, a molecule that binds PARP yet deviates significantly from the parent molecule (Fig. 1, compound 2) (17). Subsequently, a range of fluorescently and radiolabeled derivatives has been reported, of which both PARPi-FL and ^18^F-PARPi have been used to successfully measure uptake and distribution of PARP isoforms in vivo or to perform molecular radionuclide therapy (Fig. 1) (16,18–21).

**FIGURE 1.**
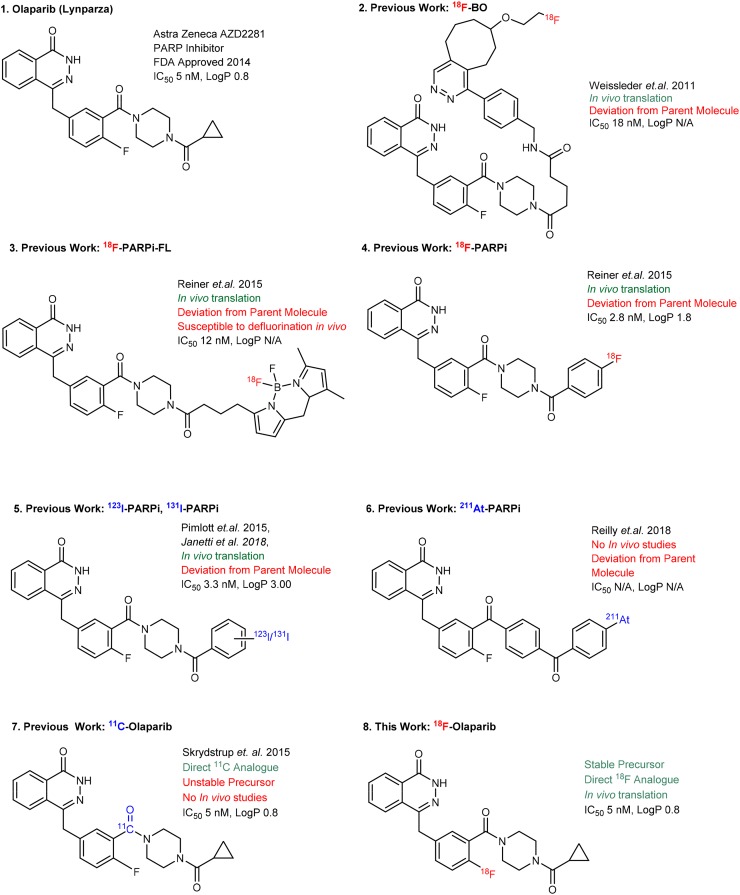
Reported olaparib-based radiolabeled PARP imaging and radionuclide therapy agents.

Alternatively, several radiolabeled compounds based on analogs of the PARP inhibitor rucaparib have been developed. Radioiodinated (^123^I, ^125^I), ^211^At-labeled, and ^18^F-labeled versions have been described (18,19,22,23). One of these compounds, ^18^F-fluorthanatrace, has been evaluated clinically, demonstrating good correlation between PARP-1 expression and ^18^F-fluorthanatrace uptake (24). In 2015, Andersen et al. reported an intricate 3-component carbonylation of aryl palladium species with the positron-emitting isotope ^11^C in the form of ^11^C-carbon monoxide (25). This gave the first direct, radiolabeled analog of olaparib, ^11^C-olaparib (Fig. 1, compound 7). The unstable nature of the palladium precursor, however, detracts from what is otherwise an elegant reaction. Moreover, ^18^F labeling would allow for a longer shelf life and results in intrinsically better spatial resolution.

Gouverneur’s group has developed a wide range of novel radiofluorination reactions, enabling radiolabeling of otherwise challenging motifs (26–28). Most significantly, a copper-mediated aromatic nucleophilic ^18^F-fluorination of aryl pinacol–derived boronic esters has enabled access to radioligands difficult to obtain applying alternative methodologies. Indeed, this method facilitated access to an isotopolog, ^18^F-labeled olaparib itself, from an appropriately protected, bench-stable, aryl boronic ester precursor (Fig. 2). Here, we demonstrate that, using ^18^F-olaparib, measurement of the distribution, uptake, and PARP binding of olaparib with PET imaging in mouse models of pancreatic ductal adenocarcinoma could be achieved. Furthermore, we report the use of ^18^F-olaparib for detecting DNA damage response after external-beam irradiation and its relationship with tumor hypoxia. To the best of our knowledge, this work represents the first radiosynthesis of ^18^F-olaparib and its in vivo translation for PET imaging.

**FIGURE 2.**
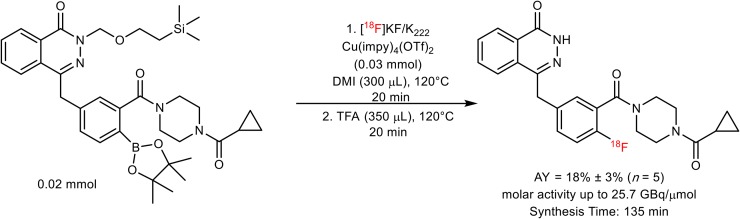
Synthesis of ^18^F-olaparib from *N*-silanyl–protected boronic pinacol ester precursor.

## MATERIALS AND METHODS

Full materials and methods are presented in the supplemental material accompanying this article (supplemental materials are available at http://jnm.snmjournals.org) (29–40).

### Synthesis

^18^F-olaparib was obtained via the copper-mediated ^18^F-fluorodeboronation of the corresponding boronic ester precursor (Fig. 2), using methodology previously described by Tredwell et al. (26).

### In Vitro Methods

PARP-1 levels were determined in a limited selection of cell lines (PSN-1, MiaPaCa-2, and CAPAN-1) using Western blot and confirmed by immunofluorescence microscopy (the supplemental material provides full details). STR profiling was used to authenticate all cell lines. CAPAN-1 cells used in this study did not match the American Type Cell Collection–held profile. However, low PARP enzyme expression was confirmed by Western blot and immunohistochemistry. Uptake of ^18^F-olaparib in cells was determined by exposing aliquots of cells growing in 24-well plates to external-beam radiation (0–10 Gy; using a ^137^Cs irradiator [0.8 Gy/min]), letting the cells recover for 2, 24, or 48 h, after which the cells were incubated with ^18^F-olaparib (50 kBq) for 30 min. Uptake was reported as a percentage of the total amount added, per milligram of total protein recovered from the isolated cells, as determined by bicinchoninic acid assay. Specificity of uptake was determined by blocking olaparib binding sites using an excess of cold, unlabeled olaparib or other PARP inhibitors (rucaparib, talazoparib; the supplemental material provides full details).

### In Vivo Methods

All animal procedures were performed in accordance with the U.K. Animals (Scientific Procedures) Act of 1986 and with local ethical committee approval. Tumor xenografts or allografts were generated by subcutaneous injection of PSN-1 or Capan-1 cell suspensions in the hind flank of BALB/c *nu/nu* animals. CaNT xenografts were generated by harvesting CaNT tumors from donor animals and injecting a tumor homogenate subcutaneously in the flank of wild-type CBA/Carl mice. A single tumor was implanted per animal. Tumors were irradiated (10 Gy, 2 Gy/min), or sham-irradiated, using a 300-kV x-ray device.

Dynamic PET images were acquired after an intravenous bolus administration of ^18^F-olaparib. Biodistribution studies, in which selected tissues were harvested, were performed 1 h after injection. Some animals were also administered an excess of cold, unlabeled olaparib for evaluating the specificity of tumor uptake (0.5 mg). EF5, a hypoxia-selective marker, was injected in some of the animals, 2 h before culling. The amount of ^18^F uptake in selected tissue was determined and reported as a percentage of the injected dose per gram of tissue (%ID/g). Digital autoradiography was performed on tumor sections. The PET images were analyzed using PMOD (the supplemental material provides full details).

All statistical analyses and nonlinear regression were performed using GraphPad Prism (GraphPad Software). Data were tested for normality and analyzed by 1-way ANOVA, with Dunnet posttests to calculate the significance of differences between groups for multiple comparisons. All data were obtained at least in triplicate, and results are reported as mean ± SD unless indicated otherwise.

## RESULTS

### ^18^F-Olaparib Synthesis via Copper-Mediated ^18^F-Fluorodeboronation of Aryl Pinacol Boronic Esters

^18^F-olaparib was prepared via copper-mediated ^18^F-fluorodeboronation of a protected boronic pinacol ester precursor (Fig. 2; Supplemental Fig. 1) in an activity yield of 18% ± 3% (non–decay-corrected radiochemical yield, *n* = 5, synthesis time of 135 min) with molar activities of up to 25.7 GBq/μmol (26). In the case of ^18^F-olaparib, additive preliminary screening experiments suggested that an unprotected boronic ester would be incompatible with the ^18^F-fluorodeboronation (Supplemental Figs. 1 and 2), potentially because of interference of the phthalazone nitrogen with the copper-complex catalyst (41). Protected boronic ester substrates bearing *N-*methyl or *N*-[2-(trimethylsilyl)ethoxymethyl] protection, respectively, underwent successful ^18^F-fluorodeboronation, as predicted by screening experiments (Supplemental Figs. 3–5). The optimal catalyst for ^18^F-fluorodeboronation of this substrate, of those tested, was shown to be Cu(OTf)_2_(impy)_4_ (impy = imidazo[1,2-*b*]pyridazine), whereas the use of 1,3-dimethyl-2-imidazolidinone proved to be the solvent that led to the optimal radiochemical yield (Supplemental Figs. 4–7; Supplemental Table 1). Crucially, an unwanted side product due to proto-deboronation of the precursor could be separated from the desired product (Supplemental Fig. 5; Supplemental Table 2), thus allowing for isolation of the pure labeled product (Supplemental Figs. 6 and 7; Supplemental Table 3).

### ^18^F-Olaparib Is Taken Up in PARP-1–Expressing Cell Lines In Vitro

**FIGURE 3.**
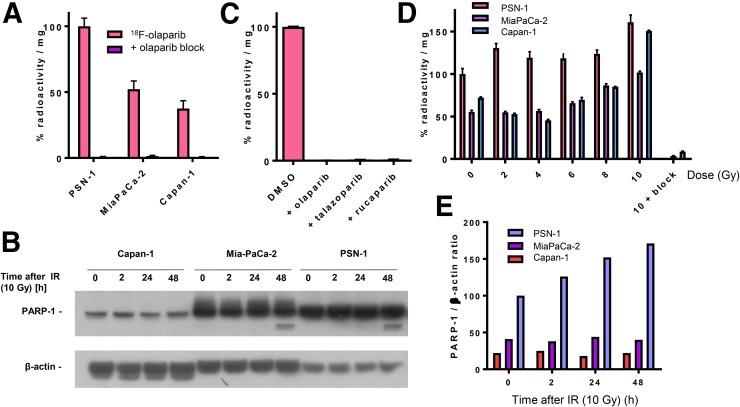
(A) Uptake of ^18^F in PSN-1, MiaPaCa-2, and Capan-1 cells 30 min after addition of ^18^F-olaparib. Uptake can be blocked using excess of cold, unlabeled olaparib. (B) Western blot probing for PARP-1 in panel of pancreatic ductal adenocarcinoma cell lines before and after 2–48 h of external-beam irradiation (10 Gy). (C) Blocking of uptake of ^18^F-olaparib in PSN-1 cells by excess of olaparib, talazoparib, or rucaparib. (D) Uptake of ^18^F-olaparib in panel of cell lines 48 h after external-beam irradiation with increasing doses. As control, uptake of ^18^F-olaparib irradiated at 10 Gy could additionally be blocked by excess of cold, unlabeled olaparib.

To demonstrate PARP targeting, a set of pancreatic ductal adenocarcinoma cell lines with varying PARP-1 expression was exposed to ^18^F-olaparib in vitro. ^18^F-olaparib was taken up within 30 min by PSN-1 cells, showing the highest uptake (Fig. 3). Results at 60 min after initial exposure of the cells to ^18^F-olaparib were similar. These results agreed with the relative PARP-1 expression levels in these cells as observed by Western blot (Fig. 3B) and immunocytochemistry (Supplemental Fig. 8). Cell-associated uptake of ^18^F-olaparib in all 3 cell lines could be blocked nearly completely (>99%) by addition of a large excess of cold, unlabeled olaparib (*P* < 0.0001), suggesting a highly specific interaction of ^18^F-olaparib with its binding sites (on PARP-1, -2, and -3), with very little nonspecific binding (Figs. 3A and 3C). In PSN-1 cells, efficient blocking was also achieved using an excess of 3 nicotinamide adenine dinucleotide pocket binding PARP inhibitors, olaparib (mean half-maximal inhibitory concentration [IC_50_], 20 nM; 95% confidence interval [95%CI], 16–25 nM), talazoparib (mean IC_50_, 5.2 nM; 95%CI, 3.2–8.5 nM), and rucaparib (mean IC_50_, 12 nM; 95%CI, 8.2–18 nM) (all *P* < 0.001) (Fig. 3C; Supplemental Fig. 9). Uptake in Capan-1 and MiaPaCa-2 cells was significantly lower than in PSN-1 cells, consistent with our findings that PARP-1 expression is lower as determined by Western blot and immunofluorescence (Fig. 3B; Supplemental Fig. 8).

### ^18^F-Olaparib Shows Hepatobiliary Clearance in Naïve Mice

**FIGURE 4.**
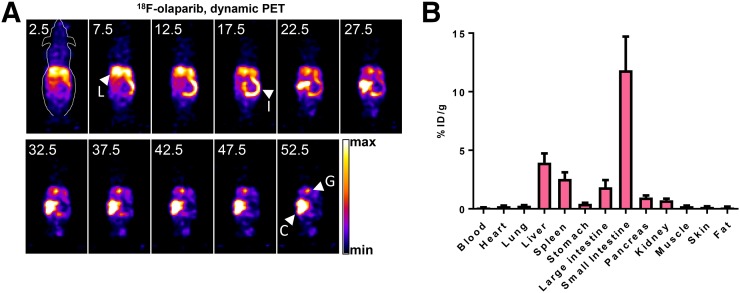
(A) Representative dynamic PET images after intravenous bolus injection of ^18^F-olaparib (3 MBq). Middle of time frames is indicated in minutes. Images are presented as maximum-intensity projections. (B) Biodistribution in wild-type CBA mice at 1 h after injection of ^18^F-olaparib. C = cecum; G = gallbladder; I = small intestine; L = liver.

Biodistribution and kinetics of ^18^F-olaparib were explored in wild-type, tumor-naïve animals. In vivo dynamic PET imaging of wild-type naïve CBA mice revealed fast pharmacokinetics and a hepatobiliary clearance pattern, after intravenous administration of 3 MBq of ^18^F-olaparib (Fig. 4A). On the basis of volume-of interest analysis of ^18^F signal originating from a region drawn around the heart, the blood clearance of ^18^F-olaparib followed a biphasic pattern (Supplemental Fig. 10), with fast and slow half-lives of 2.8 min (44%; 95%CI, 2.1–4.0 min) and 32.3 min (56%; 95%CI, 27.6–39.1 min), respectively, resulting in a weighted half-life of 19.3 min. Given the very small injected dose of compound (0.0065 mg/kg), the blood half-life of ^18^F-olaparib was markedly shorter than the previously reported half-life of 58 min (in female mice) after intravenous administration of a 20 mg/kg bolus of olaparib but slower than the 5.5 min for an ^18^F-labeled olaparib-based PARP inhibitor reported by Carney et al. (42). Removal of selected tissues after imaging and measurement of their ^18^F content confirmed the results obtained though image-based quantification (Fig. 4B), showing low blood retention and high intestinal uptake of ^18^F-olaparib at 1 h after injection.

### ^18^F-Olaparib Is Taken Up in PARP-1–Expressing Xenografts In Vivo

**FIGURE 5.**
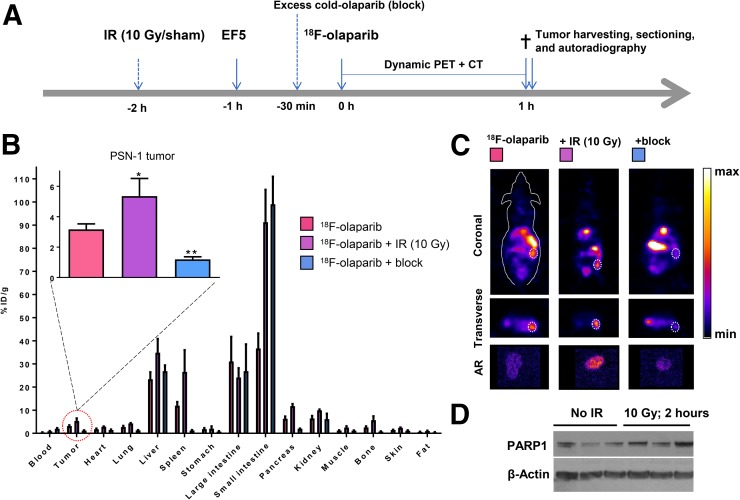
(A) Schematic of experimental design of imaging experiments. Mice were irradiated or sham-irradiated 2 h before injection of ^18^F-olaparib. (B) Biodistribution in mice bearing PSN-1 xenografts 1 h after injection of ^18^F-olaparib (3 MBq). (C) Representative coronal and transverse images of PSN-1 xenograft–bearing mice 1 h after injection of ^18^F-olaparib. Dashed lines indicate position of xenograft tumor. Insets represent autoradiograms of tumor sections, corroborating PET imaging results. (D) Western blot shows increased PARP-1 levels in 3 irradiated compared with 3 nonirradiated PSN-1 xenografts.

To study PARP targeting in tumor tissue, subcutaneous tumor–bearing mice were injected intravenously with ^18^F-olaparib. The biodistribution and clearance pattern in xenograft-bearing animals was similar to that in naïve wild-type mice (Fig. 5). Uptake in tissues expressing PARP-1, -2, and -3 such as the spleen, bone, and pancreas was observed, and this uptake could be blocked by coinjection of an excess of cold, unlabeled olaparib, demonstrating the specificity of PARP targeting. Uptake of ^18^F-olaparib in PARP-1–expressing PSN-1 xenografts amounted to 3.16 ± 0.36 %ID/g, measured by biodistribution experiments at 1 h after intravenous bolus injection. This uptake correlated with PARP-1 expression measured by Western blot and immunohistochemistry (Supplemental Figs. 11 and 12). The specificity of this uptake was demonstrated by a significant decrease in tumor uptake after coinjection of an excess of cold, unlabeled olaparib, with uptake dropping to 1.20 ± 0.17 %ID/g (*P* = 0.0016). Coronal and transverse sections through the tumor are shown in Supplemental Figure 13. Full details on the ex vivo biodistribution results are presented in Supplemental Tables 4–7.

In contrast to PSN-1 tumors, Capan-1 xenografts expressed lower levels of PARP-1, as determined by Western blot and immunohistochemistry (Fig. 5D; Supplemental Figs. 11–13). Consistent with lower tumor PARP-1 levels, and consistent with lower in vitro uptake of ^18^F-olaparib, intravenous administration resulted in lower average uptake of ^18^F-olaparib in these tumors (2.59 ± 0.32 %ID/g), although this was not statistically significant. Uptake in Capan-1 xenografts could be blocked (to 1.00 ± 0.17; *P* = 0.015), indicating specific uptake of the tracer. CaNT tumors, also expressing low amounts of murine PARP-1 (Supplemental Figs. 14 and 15) had significantly lower uptake of ^18^F-olaparib than did PSN-1 xenografts (1.71 ± 0.59 %ID/g; *P* = 0.03), and again this uptake could also be blocked by an excess of cold, unlabeled olaparib (to 0.80 ± 0.19 %ID/g). Overall, PARP-1 expression, as measured by Western blot, correlated well with the uptake of ^18^F-olaparib in xenograft tissues (*R* = 0.84, *P* < 0.0001, Supplemental Fig. 16).

### ^18^F-Olaparib Uptake in PSN-1 Cells and Xenografts Is Increased After External-Beam Irradiation

PARP-1 and -2 are essential for proficient base excision repair. To explore whether ^18^F-olaparib, which targets PARP, could be used to measure the effects of DNA-damaging therapies such as external-beam radiotherapy, we irradiated a panel of cell lines and evaluated PARP-1 expression and uptake of ^18^F-olaparib at several time intervals later. In vitro, 2 h after γ-irradiation (10 Gy) of cells, Western blot analysis revealed an increase in PARP-1 expression in MiaPaCa-2 and PSN-1 cells (Fig. 3B). PARP-1 expression in Capan-1 cells proved low (Fig. 3B), although immunofluorescence microscopy suggested some expression of PARP-1 (Supplemental Fig. 8). In line with these results, ^18^F-olaparib uptake was increased in all cells after γ-irradiation, in a radiation dose–dependent manner, which was more pronounced at later times after irradiation and in PSN-1 cells (Fig. 3D).

In vivo, 2 h after irradiation of PSN-1 xenografts (10 Gy), uptake of ^18^F-olaparib in the tumor tissue was increased by 70%, from 3.16 ± 0.36 to 5.35 ± 1.16 %ID/g (*P* = 0.025) (Fig. 5). This result was consistent with earlier data reported by Kossatz et al. (43), who used a fluorescently labeled version of olaparib, visualized using in vivo optical fluorescence imaging or ex vivo confocal microscopy. The increased expression of PARP-1 in PSN-1 xenografts was confirmed ex vivo using Western blot (Fig. 5D; Supplemental Fig. 11).

### Relationship Between Hypoxia and PARP-1 Expression

It is well known that genomic stability in tumor tissue is greatly reduced in hypoxic regions (44). To investigate the relationship between hypoxia and PARP-1 expression in vivo in tumor xenografts, we compared PARP-1 signal intensity with EF5 staining, which is known to accumulate in regions of clinically relevant hypoxia. A clear correlation between PARP-1 staining and EF5 uptake could be observed in all samples (a representative example is shown in Supplemental Fig. 17), both irradiated and nonirradiated. This observation, in combination with the increased delivery of ^18^F-olaparib to tumors with increased PARP-1 content, suggests that olaparib and other PARP inhibitors will be more effective in hypoxic tumor tissue. This prediction is consistent with previous observations by Jiang et al., who demonstrated contextual synthetic lethality for hypoxia and PARP inhibition, with hypoxic cells and tumors being up to a third more sensitive to PARP inhibition than are fully oxygenated controls (45).

## DISCUSSION

PARP inhibitors are being intensively studied after the elucidation of the role of PARylation enzymes in DNA damage repair, and the identification of the PARP enzyme, now known as PARP-1 (one of 17 in the PARP enzyme superfamily). Clinical trials were set up to study the effects of a wide variety of compounds, including olaparib (AZD2281, KU0059436), rucaparib (AG-014699, PF-01367338), veliparib (ABT-888), niraparib (MK-4827), talazoparib (BMN-673), and CEP-9722, all of which have been designed to compete for binding to the nicotinamide adenine dinucleotide binding pocket of the PARP enzymes. Most of these inhibitors are selective for PARP-1 and -2, with PARP-3 also being inhibited by some, although to a lesser degree. PARP inhibition is largely successful, especially when used in synthetic lethal combination settings, such as in preselected tumors with BRCA-ness signatures (46).

Nonetheless, resistance to PARP inhibitor treatment is common. Aside from some genetic mechanisms, the failure to deliver therapeutically adequate amounts of drug to the tumor tissue has been suggested as a likely cause, especially in brain and pancreatic tumors (10,11). It has been argued that this may be due to low systemic doses (e.g., as a result of poor oral availability), rapid clearance or metabolism, nonspecific sequestration by nontarget organs, rapid export by drug transporter (olaparib is known substrate of p-glycoprotein), lack of the target enzyme, or mutations in the binding pocket, reducing drug affinity (47). For all these reasons, using a radiolabeled form of the drug will reveal if drug accumulation in the tumor is therapeutically limiting. Imaging of PARP expression using radiolabeled PARP inhibitors, and imaging the delivery of a radiolabeled version of the drug, will assist in identifying those patients who will or will not respond to the treatment.

A variety of radiolabeled PARP inhibitors has been developed in recent years, all based on either olaparib-like structures (Reiner’s group (17,18,20,42,43), Zmuda et al. (48), Huang et al. (49), this study) or rucaparib-like structures (Makvandi’s group, Reilly et al. (19,23–25)). An excellent review on the subject has been previously published (16). Some radiolabeled PARP inhibitors are also being studied as radionuclide therapy agents (20). Although a direct comparison between ^18^F-olaparib (this work) and other radiolabeled PARP imaging agents is hampered by the different model systems that are being used, the main advantage of ^18^F-olaparib is its relatively high tumor uptake, a good contrast between PARP-expressing tumors and non–PARP-expressing tumors, and fast tumor uptake kinetics. One key challenge ^18^F-olaparib shares with all other radiolabeled PARP inhibitors is its hepatobiliary clearance pattern, making imaging in the mouse abdomen more challenging. In the clinic, this challenge could be averted by PET/CT or PET/MRI, which would make localization of lesions more straightforward, especially in ovarian and breast tumors, for which PARP inhibitors are primarily used. Being an isotopolog of the unlabeled drug olaparib, ^18^F-olaparib benefits from a wealth of clinical data already available, which will aid its translation to the clinic. As an exact chemical match of olaparib, ^18^F-olaparib allows direct imaging of the delivery of olaparib to tumor tissues and can act as a direct companion imaging biomarker for treatment prediction. The radiofluorination chemistry we have used here would allow use of a similar strategy on a much larger set of cancer drugs, since it allows radiolabeling of a much larger group of compounds than was previously possible. The use of isotopologs for PET imaging benefits from a large set of structure–activity relationship data and extensive toxicity profiling available through the drug development process. However, it does not necessarily follow that the superior therapeutic agent also makes for a better PET imaging agent, given that an imaging agent should comply with a different set of criteria. The main advantage of an isotopolog is the faster route to clinical translation that may be possible, given the reduced need for toxicity data that should be presented to the regulator.

Although PARP-1 is by far the most abundant of the DNA damage repair–relevant isoforms of PARP, and a PARP-1–selective imaging agent may predict the outcome of PARP inhibition therapy better (18), ^18^F-olaparib is not selective for one specific isoform. It has previously been well recognized that olaparib affects PARP-1, PARP-2, and PARP-3 alike, albeit with varying effects (IC_50_ of olaparib for PARP-1 and PARP-2 is 5 and 1 nM, respectively). It is also known that replacement of the cyclopropyl side chain in olaparib with a methoxy-moiety (AZD2461 (50)) significantly reduces its affinity toward PARP-3 and consequently reduces PARP-3–mediated bone marrow toxicity in a murine model. In addition, this compound has been described to have a lower affinity for p-glycoprotein drug efflux pumps (8). Here, we did not evaluate the influence of p-glycoprotein pumps for which olaparib, and thus ^18^F-olaparib, is a known substrate, although one of the cell lines we used here, Capan-1, does express some, as demonstrated by Western blot on whole-cell lysates (Supplemental Fig. 18).

Thus, different versions of radiolabeled PARP inhibitors may therefore provide subtly different readouts, and care should be taken in comparing them directly. For example, none of the small-molecule inhibitors of PARP described so far uniquely bind to PARP-1 alone. The only imaging agent with this ability is a PARP-1–selective single-domain antibody–red fluorescent protein fusion protein (51). Its use, however, is limited to transfected cells in vitro, as it cannot cross the cell membrane, thus limiting the set of currently available in vivo imaging agents for PARP to radiolabeled small molecules.

The use of PARP imaging for evaluating irradiated tissue was first explored by Kossatz et al. using a fluorescently labeled version of olaparib (43). Molecular imaging of the biologic effects of radiation therapy holds great promise for precision cancer therapy and may aid in tailoring radiotherapy dose schedules to the individual tumor’s response. Previously, we explored an imaging agent targeting γH2AX for this purpose, ^111^In- or ^89^Zr-labeled anti-γH2AX-TAT (15,52–54). γH2AX is a phosphorylated histone expressed around DNA double-strand breaks and has been used extensively as a radiation damage marker. Given that this imaging agent is based on an antibody, clinical translation may be more challenging. Therefore, imaging the radiation damage response using radiolabeled PARP inhibitors may offer an alternative, since some are already being evaluated in early-phase clinical trials.

We hypothesize that, when translated to the clinic, PET imaging with radiolabeled compounds such as ^18^F-olaparib will allow better patient selection by determining tumor drug uptake; measurement of the biologic effects of genotoxic cancer treatment, such as chemo- and radiotherapy; and better patient stratification, making the therapeutic use of PARP inhibitors even more effective, albeit in a more stringently selected patient population. One early phase 1 clinical trial using a PARP inhibitor–based imaging agent is now published (using ^18^F-fluorthanatrace; www.clinicaltrials.gov), the results of which will undeniably inform the field of future directions (55).

## CONCLUSION

Taken together, we show here that ^18^F-olaparib can be used for quantifying olaparib tumor accumulation in vitro and in vivo. This study serves as an expansion of the evidence base for the use of PARP imaging agents for clinical PET. The use of the ^18^F-labeled isotopolog of olaparib allows direct prediction of the distribution of olaparib, given its exact structural likeness to the native, nonradiolabeled drug. The translation of this diagnostic strategy to the clinic will significantly improve patient stratification as well as therapy response monitoring.

## DISCLOSURE

This research was supported by Pancreatic Cancer U.K.; the Pancreatic Cancer Research Fund; CRUK through the Oxford Institute for Radiation Oncology, the CRUK Oxford Centre, and the CRUK/EPSRC Imaging Centre in Oxford; the EPSRC (EP/L025604/1, NS/A000024/1); and CRUK C5255/A16466. The work herein is subject to a U.K. priority patent application. This research was also supported by a Wolfson Research Merit Award from the Royal Society (Véronique Gouverneur) and a Junior Group Leader award from Cancer Research U.K. (Bart Cornelissen). No other potential conflict of interest relevant to this article was reported.
