# Supplementary material for: PET Imaging of PARP Expression Using 18F-Olaparib
Source: J Nucl Med. 2019 Apr;60(4):504–10. doi: 10.2967/jnumed.118.213223 (PMC6448459; doi:10.2967/jnumed.118.213223)
Supplement: Supplementary file 1 [file jnm213223SupplementalData.pdf]

1. Supplemental Material: Chemistry

All chemicals were purchased from Acros, Alfa Aesar, Fluorochem, Sigma-Aldrich and used as received without further purification. Solvents were purchased from Fisher, Rathburn or Sigma-Aldrich. When anhydrous solvents were required they were purified by expression through an activated alumina column built as described by Pangborn and Grubbs (*1*). Reactions were monitored by thin-layer chromatography (TLC) carried out on Merck Kiesegel 60 F254 plates, silica gel column chromatography was performed over Merck silica gel C60 (40-60 µm). All NMR spectra were recorded on Bruker DPX200, AV400, AVB400, AVC500, AVB500 and DRX500 spectrometers. Proton and carbon-13 NMR spectra are reported as chemical shifts (δ) in parts per million (ppm) relative to the solvent peak using the Bruker internal referencing procedure (edlock). Fluorine-19 NMR spectra are referenced relative to $CFCl_3$ in $CDCl_3$. Coupling constants (J) are reported in units of hertz (Hz). The following abbreviations are used to describe multiplicities – s (singlet), d (doublet), t (triplet), q (quartet), m (multiplet), brs (broad singlet). High resolution mass spectra (HRMS, m/z) were recorded on a Bruker MicroTOF spectrometer using positive electrospray ionization ($ESI^{+)}$ or on a Micromass GCT spectrometer using filed ionization (FI^+) or chemical ionization (CI^+). Infrared spectra were recorded either as the neat compound or in a solution using a Bruker Tensor 27 FT-IR spectrometer. Absorptions are reported in wavenumbers (cm^{-1}) and only peaks of interest are reported. Optical rotations were measured on a PerkinElmer Polarimeter model 341 Specific rotations are reported in 10^{-1} deg cm^2 g^{-1} and concentrations in g/100 mL. Melting points of solids were measured on a Griffin apparatus and are uncorrected. IUPAC names were obtained using the ACD/I-Lab service.

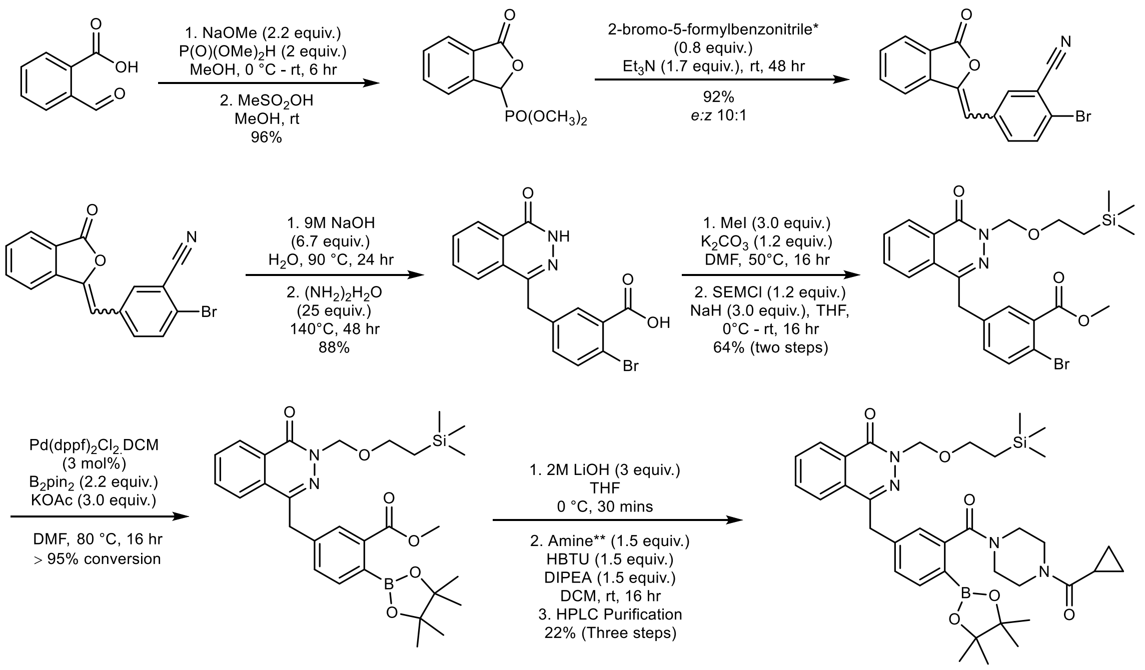

Supplemental Figure 1. Synthesis of 4-(3-(4-(cyclopropanecarbonyl)piperazine-1-carbonyl)-4-(4,4,5,5-tetra methyl-1,3,2-dioxaborolan-2-yl)benzyl)-2-((2-trimethlsilyl)ethoxy)methyl)phthalazin-1(*2H*)-one. *2-bromo-5-formylbenzonitrile was synthesized over two steps from 2-amino-5-bromobenzo-nitrile *via* formylation/Sandmeyer (46% over two steps), ***N*-(cyclopropylcarbonyl)piperazine.

1. Synthesis methods

See Supplemental Figure 1 for the full synthesis scheme. For each compound relating to supplemental figure 1, where appropriate, a commentary is provided to aid the reader in translating this chemistry to other laboratories.

Dimethyl (3-oxo-1,3,-dihydroisobenzofuran-1-yl)phosphonate

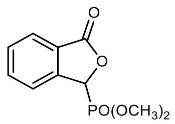

Dimethylphosphite (0.92 mL, 10.0 mmol) was added dropwise to a solution of sodium (0.58 g, 10.8 mmol) in MeOH (15 mL) at 0 °C. To the solution, 2-carboxybenzaldehyde (1.00 g, 6.66 mmol) was added portion-wise while stirring. The mixture was gradually warmed to room temperature and stirred for 6 hours. Methanesulfonic acid (0.77 mL, 11.9 mmol) was added dropwise and the mixture was stirred for another 30 minutes. The solution was concentrated in vacuo to produce a white solid, to which water was added (30 mL) and the crude product was extracted into DCM (3 × 30 mL). The organic layer was washed with water (2 × 30 mL), dried with $MgSO_4$ and filtered. The filtrate was concentrated in vacuo and washed with Et_2O (3 × 20 mL), affording Dimethyl-(3-oxo-1,3-dihydrobenzofuran-1-yl)phosphonate (1.54 g, 96%) as a white solid.

^{1}H NMR (400 MHz, CD_3OD) δ = 7.94 (d, *J* = 7.5 Hz 1H), 7.83 (t, *J* = 7.5 Hz, 1H), 7.75 (d, *J* = 7.5 Hz, 1H), 7.68 (t, *J* = 7.5 Hz, 1H), 6.10 (d, *J* = 10.8, 1H), 3.91 (d, *J* = 10.8 Hz, 3H), 3.72 (d, *J* = 10.8 Hz, 3H). Data is in accordance with known literature; **Mp**: 90 - 92°C.(*2*)

2-Fluoro-5-((3-oxoisobenzofuran-1(*3H*)-ylidene)methyl)benzonitrile

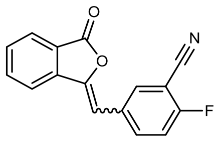

A solution of Dimethyl-(3-oxo-1,3-dihydrobenzofuran-1-yl)phosphonate (1.00 g, 4.13 mmol) and 2-fluoro-5-formylbenzonitrile (0.62 g, 4.13 mmol) in THF (50 mL) was prepared at room temperature. The solution was then cooled to 0 °C followed by the addition of Et_3N (0.69 mL, 4.96 mmol). The reaction mixture was allowed to warm up to room temperature and was stirred for 48 h, followed by concentration in vacuo to produce a white solid. The solid was suspended in water, collected by vacuum filtration and washed with hexane (2 × 20 mL), Et_2O (2 × 20 mL), and MeOH (2 × 20 mL) affording 2-Fluoro-5-((3-oxoisobenzofuran-1(*3H*)-ylidene)methyl)benzonitrile (0.88 g, 80%) as a white solid.

NMR spectra showed a 3:1 mixture of E and Z isomers. Where possible, shifts are assigned to each respective isomer. **^{1}H NMR** (400 MHz, DMSO-d_6) δ = 8.21 – 8.12 (m, 1H), 8.08 (dt, J 8.0, 1.0 Hz, 1H), 8.00 – 7.97 (m, 1H), 7.92 (t, J 7.6 Hz, 1H), 7.75 – 7.67 (m, 1H), 7.65 (t, J 9.0 Hz, 1H), 6.98 (s, 1H); **Mp**: 163 - 165°C. Data is in accordance with known literature.(*2*)

2-Fluoro-5-((4-oxo-3,4,-dihydrophthalazin-1-yl)methyl)benzoic acid

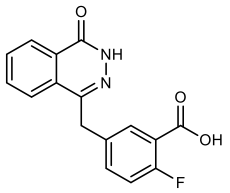

2-Fluoro-5-[(3’-oxo-2’- benzofuran-1’-ylidene)methyl]benzonitrile (0.50 g, 1.89 mmol) was suspended in water (3 mL) and 13 M NaOH was added (0.67 mL). The mixture was heated to 90 °C and stirred for 24 h, after which it was cooled to 70 °C, followed by the addition of hydrazine monohydrate (1.34 mL, 26.9 mmol) and a further 72 h of stirring. The mixture was then cooled to room temperature and acidified with 8 M HCl to an approximate pH of 4. The solid precipitate was collected by vacuum filtration and washed with water (3 × 25 mL) and Et_2O (4 × 25 mL) affording 2-Fluoro-5-((4-oxo3,4-dihydrophthalazin-1-yl)methyl)benzoic acid (0.38 g, 67%) as a red solid.(*2*)

^{1}H NMR (400 MHz, DMSO-d_6) δ = 12.57 (s, 1H), 8.26 (dd, *J* = 7.8, 0.8 Hz, 1H), 7.98 (d, *J* = 7.9 Hz, 1H), 7.93 – 7.86 (m, 1H), 7.86 – 7.79 (m, 2H), 7.61 – 7.54 (m, 1H), 7.23 (dd, J 10.8, 8.5 Hz, 1H), 4.35 (s, 2H); **{^{1}H}^{19}F NMR** (376 MHz, DMSO-d_6) δ = -114.0; **Mp**: 217 - 219°C. Data is in accordance with known literature.(*2*)

4-(3-(4-(cyclopropanecabonyl)piperazine-1-carbnonyl)-4-fluorobenzyl)phthalazin-1(*2H*)-one (Olaparib)

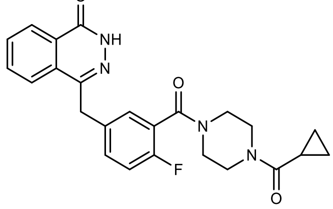

To a solution of 2-Fluoro-5-((4-oxo3,4-dihydrophthalazin-1-yl)methyl)benzoic acid (50 mg, 0.168 mmol) in DMA (1 mL) was added DIPEA (56 *µ*L, 0.336 mmol) and HBTU (64 mg, 0.170 mmol). The reaction mixture was stirred for 1 hour before addition of cyclopropylpiperazine-1-ylmethanone (0.170 mmol) was carried out. The reaction mixture was stirred at room temperature for 48 h. The reaction mixture was then extracted with DCM (3 × 5 mL) and washed with water (3 × 20 mL). The organic layers were collected, dried with $MgSO_4$ and the excess solvent removed *in vacuo*. Purification

via reverse phase HLPC was carried out affording 4-(3-(4 (cyclopropanecarbonyl) piperazine-1-carbonyl)-4-fluorobenzyl)phthalazin-1(*2H*)-one (olaparib) (25 mg, 34**%**) as a white solid.

^{1}H NMR (400 MHz, $CDCl_3$) δ = 10.65 (s, 1H), 8.44 – 8.37 (m, 1H), 7.75 – 7.61 (m, 3H), 7.34 – 7.22 (m, 2H), 6.97 (t, *J* = 8.9 Hz, 1H), 4.22 (s, 2H), 3.90 – 3.09 (m, 8H), 1.79 – 1.52 (s, 3H), 0.99 – 0.88 (m, 2H), 0.81 – 0.63 (s, 2H); **{^{1}H}^{19}F NMR** (376 MHz, $CDCl_3$) δ = - 117.6; **Mp**: 69 - 71°C. Data is in accordance with known literature.(*2*)

2-Amino-5-formylbenzonitrile

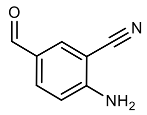

To a flame-dried round bottom flask was added 2-amino-5-bromo-benzonitrile (1.00 g, 5.08 mmol) and THF (30 mL). The solution was cooled to – 78 °C before *n*-BuLi in hexanes (2.5 M, 4.49 mL, 11.2 mmol) was added dropwise. The reaction was left to stir at -78 °C for 2 h before quenching with DMF (0.92 mL, 12.7 mmol) and allowed to warm to room temperature. The solution was then extracted with $NaHCO_3$ (40 mL) and washed with DCM (3 x 30 mL). The organic layers were collected, dried with $MgSO_4$, and the excess solvent removed *in vacuo*. The crude material was then purified *via* flash column chromatography (*n*-Pent:EtOAc 3:1) affording 2-amino-5-formylbenzonitrile (0.53 g, 71%) as a pale yellow solid.

^{1}H NMR (400 MHz, $CDCl_3$) δ = 9.76 (s, 1H), 7.92 (d*,* *J* = 1.8 Hz, 1H), 7.87 (dd, *J* = 8.6, 1.8 Hz, 1H), 6.82 (d, *J* = 8.6 Hz, 1H), 5.00 (bs, 2H); **^{13}C NMR** (101 MHz, $CDCl_3$) δ = 188.6, 153.7, 136.5, 134.3, 127.4, 116.2, 115.1, 95.9. The data is in accordance with known literature.(*3*)

2-Bromo-5-formylbenzonitrile

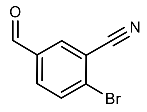

To a round bottom flask containing 2-Amino-5-formylbenzonitrile (17.95 g, 126 mmol) at 0 °C was added 6M HCl (107 mL) and fuming H_2SO_4 (107 mL). After being allowed to cool, a solution of sodium nitrite (18.5 g, 268 mmol) in H_2O (40 mL) was added dropwise before allowing the reaction to stir for 30 minutes. The reaction mixture was then added dropwise to a solution of copper(II) bromide (40.1 g, 179 mmol) in 48% HBr (107 mL) at 0 °C. The reaction was then stirred at 0 °C for 60 min before being allowed to warm to room temperature and being stirred for another hour. Upon completion, the reaction was poured into an ice/water mixture before the organic layer was extracted with DCM (3 x

150 mL). The organic layer was then dried with $MgSO_4$ and the excess solvent removed *in vacuo*. The crude material was purified by flash column chromatography (*n*-Pent:EtOAc 10:1) to afford 2-Bromo-5-formylbenzonitrile as a pale yellow solid (16.8 g, 80.6 mmol, 64%).

^{1}H NMR (400 MHz, $CDCl_3$) δ = 10.00 (s, 1H), 8.14 (s, 1H), 7.95 (d, 8.4, 1H), 7.90 (d, *J* = 8.3 Hz, 1H); **^{13}C NMR** (101 MHz, $CDCl_3$) δ = 188.9, 135.4, 135.1, 134.3, 133.7, 131.9, 117.2, 116.1; **IR** (*v,* cm^{-1}): 1702, 1562, 1174, 1008; **HRMS** (ESI) for $C_8H_5{}^{79}BrNO$ $[M+H]^+$ requires 209.9549 found 209.9551; **Mp**: 108 – 110 °C.

2-Bromo-5-((3-oxoisobenzofuran-1(*3H*)-ylidene)methyl)benzonitrile

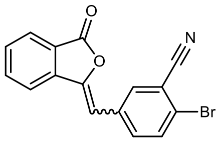

A solution of dimethyl-(3-oxo-1,3-dihydrobenzofuran-1-yl)phosphonate (5.00 g, 20.7 mmol) and 2-bromo-5-formylbenzonitrile (3.58 g, 17.2 mmol) in THF (100 mL) was prepared at room temperature. The solution was cooled to 0 °C followed by the addition of Et_3N (4.78 mL, 34.4 mmol). The reaction mixture was warmed to room temperature and was stirred for 48 h, followed by concentration in vacuo to produce a white solid. The solid was suspended in water, collected by vacuum filtration and washed with hexane (2 × 20 mL) and Et_2O (3 × 20 mL) affording 2-Bromo-5-((3-oxoisobenzofuran-1(*3H*)-ylidene)methyl)benzonitrile (5.14 g, 15.8 mmol, 92%) as a white solid in a mixture of *e:z* stereoisomers (10:1) and a purity of 90%. NMR spectra showed a 10:1 mixture of E and Z isomers.

^{1}H NMR (400 MHz, DMSO-d$_6$) δ = 8.24 – 8.06 (m, 2H, 2H*), 8.01 – 7.90 (m, 3H, 3H*), 7.83 – 7.50 (m, 2H, 2H*), 7.00 (s, 1H*), 6.97 (s, 1H) (Where possible, shifts are assigned to each respective isomer); **^{13}C NMR** (100 MHz, DMSO-d$_6$) δ = 166.3, 146.6, 140.0, 135.9, 135.7, 135.4, 134.3, 134.1, 132.0, 131.6, 125.9, 123.9, 121.5, 117.5, 115.5, 103.9 (only those peaks corresponding to the major isomer are reported); **IR** (*v,* cm^{-1}): 2977, 2225, 1776, 1643, 1495, 954, 745, 681.

; **HRMS** (ESI) for $C_{16}H_9{}^{79}BrNO_2$ $[M+H]^+$ requires 325.9746 found 325.9745; **Mp**: 118 – 120 °C.

2-Bromo-5-((4-oxo-3,4-dihydrophthalazin-1-yl)methyl)benzoic acid

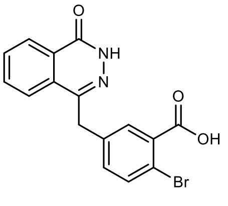

2-Bromo-5-[(3′-oxo-2′- benzofuran-1′-ylidene)methyl]benzonitrile (12.0 g, 36.9 mmol) was suspended in water (75 mL) and 13 M NaOH was added (19 mL, 247 mmol). The mixture was heated to 90 °C and stirred for 24 h, after which it was warmed to reflux (140 °C), followed by the addition of hydrazine monohydrate (25.0 mL, 780 mmol) and a further 48 h of stirring. The mixture was then cooled to room temperature and acidified with 5 M HCl to an approximate pH of 2. The solid precipitate was collected by vacuum filtration and washed with water (50 mL) and Et_2O (3 × 50 mL) affording 2-Bromo-5-((4-oxo3,4-dihydrophthalazin-1-yl)methyl)benzoic acid (12.27 g, 88%) as a red solid.

^{1}H NMR (400 MHz, DMSO-d_6) δ = 12.61 (s, 1H), 8.25 (dd, *J* = 7.7, 1.4 Hz, 1H), 7.99 – 7.94 (m, 1H), 7.89 (ddd, *J* = 8.1, 7.1, 1.5 Hz, 1H), 7.83 (td, *J* = 7.5, 1.2 Hz, 1H), 7.71 (d, *J* = 2.3 Hz, 1H), 7.62 (d, *J* = 8.2 Hz, 1H), 7.36 (dd, *J* = 8.2, 2.3 Hz, 1H), 4.33 (s, 2H); **^{13}C NMR** (100 MHz, DMSO-d_6) δ = 167.7, 159.8, 145.2, 138.5, 134.3, 134.1, 133.4, 132.1, 131.4, 129.5, 128.3, 126.5, 125.9, 118.4, 100.0, 36.9; **IR** (*v,* cm^{-1}): 2890, 1771, 1472, 1394, 970, 760, 689; **HRMS** (ESI) for $C_{16}H_{10}{}^{79}BrN_2O_3$ $[M-H]^-$ requires 356.9945 found 356.9948; **Mp**: > 250 °C.

Methyl-2-bromo-5-((4-oxo-3,4-dihydrophthalazin-1-yl)methyl)benzoate

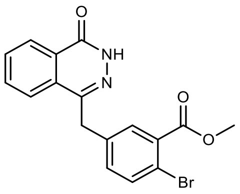

To a solution of 2-Bromo-5-((4-oxo-3,4-dihydrophthalazin-1-yl)methyl)benzoic acid (12.32 g, 34.4 mmol) in anhydrous DMF (100 mL) was added iodomethane (6.38 mL, 103.2 mmol) and anhydrous potassium carbonate (5.70 g, 41.3 mmol). The reaction was then heated to 50 °C and stirred overnight. Upon completion, the excess solvent was removed *in vacuo* and water (100 mL) was added to the crude material. The precipitate formed was filtered and washed with Et_2O (3 x 100 mL) before drying *in vacuo* affording Methyl-2-bromo-5-((4-oxo-3,4-dihydrophthalazin-1-yl)methyl) benzoate as a dull brown solid (11.4 g, 30.6 mmol, 89%).

^{1}H NMR (400 MHz, DMSO-d_6) δ = 12.60 (s, 1H), 7.99 – 7.93 (m, 1H), 7.90 (ddd, *J* = 8.1, 7.1, 1.5 Hz, 1H), 7.83 (ddd, *J* = 8.4, 7.2, 1.3 Hz, 1H), 7.73 (d, *J* = 2.2 Hz, 1H), 7.67 (d, *J* = 8.2 Hz, 1H), 4.35 (s, 2H), 3.83 (s, 3H); **^{13}C NMR** (100 MHz, DMSO-d_6) δ = 166.6, 159.8, 145.1, 138.6, 134.4, 134.1, 134.0, 132.8, 132.1, 131.6, 129.5, 128.3, 126.5, 125.9, 118.5, 53.1, 36.9; **IR** (*v,* cm^{-1}): 2983, 1650, 1250, 1205, 1104, 772; **HRMS** (ESI) for $C_{17}H_{13}{}^{79}BrN_2{}^{23}NaO_3$ $[M+Na]^+$ requires 395.0002 found 395.0001; **Mp**: 211 – 213 °C.

Methyl-2-bromo-5-((4-oxo-((2-(trimethylsilyl)ethoxy)methyl)-3,4-dihydro phthalazin-1-yl)methyl) benzoate

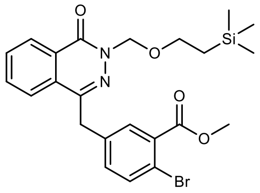

To a round bottom flask under an atmosphere of argon containing Methyl-2-bromo-5-((4-oxo-((2-(trimethylzilyl)ethoxy)methyl)-3,4-dihydro phthalazin-1-yl)methyl) benzoate (11.0 g, 29.6 mmol) at 0 °C was added anhydrous THF (200 mL) and sodium hydride (60% in dispersion oil, 3.55 g, 88.8 mmol). The reaction was stirred at 0 °C for 30 min before warming to room temperature upon which 2-(trimethylsilyl)ethoxymethyl chloride (6.25 mL, 35.6 mmol) was added dropwise. The reaction was stirred overnight before the excess solvent was removed *in vacuo* and purified directly *via* flash column chromatography (*n*-Pent:EtOAc 10:3) affording Methyl-2-bromo-5-((4-oxo-((2-(trimethylsilyl)ethoxy)methyl)-3,4-dihydro phthalazine-1-yl)methyl)benzoate as a white solid (10.7 g, 72%, 21.3 mmol).

^{1}H NMR (400 MHz, $CDCl_3$) δ = 8.52 – 8.45 (m, 1H), 7.77 – 7.68 (m, 3H), 7.66 – 7.58 (m, 1H), 5.58 (s, 2H), 4.28 (s, 2H), 3.91 (s, 3H), 3.79 – 3.73 (m, 2H), 1.02 – 0.97 (m, 2H), 0.01 (s, 9H); **^{13}C NMR** (100 MHz, $CDCl_3$) δ = 167.8, 161.3, 145.7, 138.6, 136.0, 134.9, 133.9, 133.8, 133.0, 132.6, 130.5, 129.8, 129.2, 126.3, 121.3, 80.4, 68.6, 54.0, 39.5, 19.5, 0.00 (3C); **IR** (*v*, cm^{-1}): 2952, 1721, 1652, 1431, 1294, 1223, 1074, 1027, 854, 832, 745; **HRMS** (ESI) for $C_{23}H_{27}{}^{79}BrN_2{}^{23}NaO_4{}^{28}Si$ $[M+Na]^+$ requires 525.0826 found 525.0827; **Mp**: 85 – 87 °C.

4-(3-(4-(cyclopropanecarbonyl)piperazine-1-carbonyl)-4-(4,4,5,5-tetramethyl-1,3,2-dioxaborolan-2-yl)benzyl)-2-((2-trimethlsilyl)ethoxy)methyl)phthalazin-1(*2H*)-one

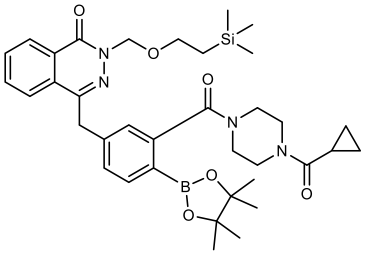

To a Schlenk tube under an atmosphere of argon was added, Methyl-2-bromo-5-((4-oxo-((2-(trimethylsilyl)ethoxy) methyl)-3,4-dihydro phthalazin-1-yl)methyl)benzoate (1.48 g, 2.94 mmol), bis(pinacolato)diboron (1.49 g, 5.89 mmol), Pd(dppf)Cl_2 (72 mg, 0.09 mmol) and potassium acetate (0.87 g, 8.88 mmol). The reaction flask was then backfilled with argon and placed in an oil bath at 90 °C upon which degassed DMF (13 mL) was added and the reaction left to stir overnight. Upon

completion, the reaction was cooled and passed through a plug of Celite before extracting with EtOAc (100 mL) and washing with Lithium Chloride solution (3 x 50 mL). The organic phases were then collected and the excess solvent was removed *in vacuo.* The crude material was then purified by flash column chromatography (10:3 *n*-Pent: EtOAc) to afford a mixture of Methyl-5-((4-oxo-3-((2-(trimethylsilyl)ethoxy)methyl)-3,4-dihydro phthalazin-1-yl)methyl)-2-(4,4,5,5-tetramethyl-1,3,2-dioxaborolan-2-yl)benzoate and bis(pinacolato)diboron (2.00 g) with the following ^{1}H NMR spectra being observed. **^{1}H NMR** (400 MHz, $CDCl_3$) δ = 8.43 (d, *J* = 7.8 Hz, 1H), 7.85 (s, 1H), 7.76 – 7.57 (m, 2H), 7.53 (d, *J* = 8.0 Hz, 1H), 7.38 (s, 2H), 5.58 (s, 2H), 4.32 (s, 2H), 3.86 (s, 3H), 3.76 (t, *J* = 7.3 Hz, 1H), 1.37 (s, 12H), 0.99 (t, *J* = 7.6 Hz, 2H), - 0.03 (s, 9H). Peaks reported only correspond to the product. The crude material was taken forward without further purification.

To a round bottom flask containing Methyl-5-((4-oxo-3-((2-(trimethylsilyl)ethoxy) methyl)-3,4-dihydrophthalazin-1-yl) methyl)-2-(4,4,5,5-tetramethyl-1,3,2-dioxaborolan-2-yl)benzoate and bis(pinacolato) diboron (2.00 g) was added THF (100 mL) before cooling to 0 °C. Upon cooling, 2M lithium hydroxide (7 mL, 14 mmol) was added dropwise before allowing to reaction to stir for 30 min at 0 °C. 1M HCl was then added dropwise to the reaction mixture at 0 °C until reaching pH 4. The mixture was then extracted with EtOAc (3 x 80 mL) and washed with Brine (3 x 50 mL). The organic phases were combined, and the excess solvent removed *in vacuo*. The crude material (1.4 g) was transferred to a round bottom flask upon which DCM (100 mL), HBTU (2.97 g, 7.84 mmol) and DIPEA (1.36 mL, 7.84 mmol) were added. After stirring at room temperature for 30 minutes, *N*-cyclopropylcarbonylpiperazine (1.11 mL, 7.85 mmol) was added and the reaction stirred overnight. After stirring for 16 hours, the excess solvent was removed *in vacuo* and the crude material isolated by flash column chromatography (Pure EtOAc) before purification by reverse phase HPLC was carried out to afford 4-(3-(4-(cyclopropanecarbonyl) piperazine-1-carbonyl)-4-(4,4,5,5-tetramethyl-1,3,2-dioxaborolan-2-yl)benzyl)-2-((2-trimethlsilyl) ethoxy)methyl)phthalazin-1(*2H*)-one (419 mg, 0.62 mmol, 22% - three steps) as a white solid in a mixture of rotamers. In each case the shift relating to the minor rotamer has been donated with an asterisk.*

^{1}H NMR (400 MHz, $CDCl_3$) δ = 8.46 (dd, *J* = 7.8, 1.6 Hz, 1H + 1H*), 7.79 – 7.55 (m, 4H + 4H*), 7.32 – 7.28 (m, 1H + 1H*), 7.14 (bs, 1H + 1H*), 5.59 (s, 2H + 2H*), 4.33 (s, 2H + 2H*), 3.87 – 3.04 (m, 10H + 10H*), 1.28 (s, 12H + 12H*), 1.04 – 0.97 (m, 4H + 4H*), 0.90 – 0.85 (m, 1H + 1H*), 0.83 – 0.71 (m, 2H + 2H*), 0.00 (s, 9H + 9H*); **^{13}C NMR** (100 MHz, $CDCl_3$) δ = 172.3 (1C + 1C*), 171.0 (1C + 1C*), 160.0 (1C + 1C*), 144.7 (1C + 1C*), 142.9 (1C + 1C*), 141.3 (1C + 1C*), 136.3 (1C + 1C*), 133.3 (1C + 1C*), 131.5 (1C + 1C*), 129.2 (1C + 1C*), 128.4 (1C + 1C*), 128.2 (1C + 1C*), 127.7 (1C + 1C*), 125.4 (1C + 1C*), 125.2 (1C + 1C*), 84.1 (2C + 2C*), 79.0 (1C + 1C*), 67.3 (1C + 1C*), 47.3 (1C*), 46.9 (1C), 45.2 (1C*),

44.8 (1C), 41.9 (1C*), 41.7 (2C), 41.4 (1C*), 39.1 (1C + 1C*), 24.9 (4C + 4C*), 18.1 (1C + 1C*), 11.1 (1C + 1C*), 7.66 (2C + 2C*), -1.36 (3C + 3C*) (the carbon bearing the boron substituent is not observed); **IR** (*v,* cm^{-1}): 2981, 2889, 2360, 2341, 1641, 1382, 1354, 1146, 1087, 956; **HRMS** (ESI) for $C_{36}H_{49}N_4O_6{}^{10}B^{23}Na^{28}Si$ $[M+Na]^+$ requires 694.34429 found 694.34418; **Mp**: 78 – 80 °C.

2-Methylphthalazin-1(*2H*)-one

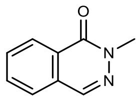

To a round bottom flask containing DMF (10 mL) under an atmosphere of argon was added, phthalazone (300 mg, 2.05 mmol). The solution was then cooled to 0 °C before sodium hydride (60% in dispersion oil, 61 mg, 2.53 mmol) was added portion wise. Finally, iodomethane (119 µL, 1.92 mmol) was added and the reaction was stirred overnight before the solvent was removed *in vacuo* and the crude material purified directly *via* flash column chromatography using *n*-pentane: EtOAc (10:1) affording 2-Methylphthalazin-1(*2H*)-one as a white solid (210 mg, 1.31 mmol, 64%).

^{1}H NMR (400 MHz, $CDCl_3$) δ = 8.42 – 8.38 (m, 1H), 8.12 (s, 1H), 7.81 – 7.70 (m, 2H), 7.69 – 7.65 (m, 1H), 3.83 (s, 3H); **^{13}C NMR** (100 MHz, $CDCl_3$) δ = 159.7, 137.6, 133.0, 131.6, 129.8, 127.8, 126.5, 126.0, 39.5. The data is in accordance with known literature.(*4*)

***tert*-Butyl 1-oxophthalazine-2(*1H*)-carboxylate**

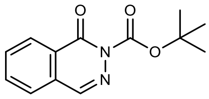

To a round bottom flask containing THF (10 mL) under an atmosphere of nitrogen was added, phthalazone (500 mg, 3.41 mmol), di-*tert*-butyl dicarbonate (1.11 g, 5.12 mmol) and DMAP (620 mg, 5.12 mmol). The reaction was stirred overnight at room temperature before the solvent was removed *in vacuo* and the crude material purified directly *via* flash column chromatography using *n*-pentane: EtOAc (5:1) *tert*-Butyl 1-oxophthalazine-2(*1H*)-carboxylate as a white solid (765 mg, 3.11 mmol, 91%).

^{1}H NMR (400 MHz, $CDCl_3$) δ = 8.43 (dd, *J* = 7.9, 1.3 Hz, 1H), 7.83 (td, *J* = 7.5, 1.4 Hz, 1H), 7.77 (td, *J* = 7.6, 1.3 Hz, 1H), 7.68 (dd, *J* = 7.5, 1.3 Hz, 1H), 1.66 (s, 9H);**^{13}C NMR** (100 MHz, $CDCl_3$) δ = 158.3, 150.9, 138.9, 134.2, 132.3, 129.2, 128.7, 127.5, 126.4, 85.9, 27.8 (3C); **IR** (*v,* cm^{-1}): 2980, 1720, 1641, 1470, 1325, 1130; **HRMS** (ESI) for $C_{13}H_{14}N_2O_3$ $[M+H]^+$ requires 247.1012 found 247.1015; **Mp**: 81 – 83 °C.

2-allylphthalazin-1(*2H*)-one

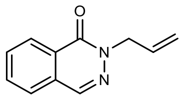

To a round bottom flask containing DMF (30 mL) under an atmosphere of nitrogen was added, phthalazone (500 mg, 3.42 mmol), anhydrous potassium carbonate (708 mg, 5.12 mmol) and allyl bromide (442 µL, 5.12 mmol). The reaction was stirred overnight at room temperature before the solvent was removed *in vacuo* and the crude material purified directly *via* flash column chromatography using *n*-pentane: EtOAc (3:1) affording 2-allylphthalazin-1(*2H*)-one as a brown oil (222 mg, 1.20 mmol, 35%)

^{1}H NMR (400 MHz, $CDCl_3$) δ = 8.42 (ddd, *J* = 7.5, 1.7, 0.7 Hz, 1H), 8.17 (d, *J* = 0.8 Hz, 1H), 7.83 – 7.72 (m, 2H), 7.71 – 7.67 (m, 1H), 6.04 (ddt, *J* = 17.2, 10.3, 5.9 Hz, 1H), 5.30 – 5.22 (m, 2H), 4.85 (dt, *J* = 5.9, 1.5 Hz, 2H); **^{13}C NMR** (100 MHz, $CDCl_3$) δ = 159.2, 138.1, 133.1, 132.5, 131.7, 129.7, 128.0, 126.8, 126.0, 118.0, 53.5. Data is in accordance with literature values.(*5*)

2-((2-triethylsilyl)ethoxyl)methyl)phthalazin-1(*2H*)-one

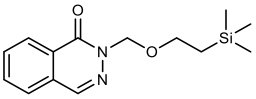

To a round bottom flask containing THF (10 mL) under an atmosphere of argon was added, phthalazone (480 mg, 3.28 mmol). To solution was then cooled to 0 °C before sodium hydride (60% in dispersion oil, 97 mg, 4.04 mmol) was added portion wise. The reaction was then stirred for 30 min at 0 °C before warming to room temperature after which 2-(trimethylsilyl)ethoxymethyl chloride (0.70 mL, 3.97 mmol) was added dropwise. The reaction mixture was left to stir overnight after which the solvent was removed *in vacuo*. The crude mixture was then extracted with DCM (3 x 10 mL) and washed with brine (3 x 10 mL). The organic layer was collected, dried with magnesium sulfate and the excess solvent removed *in vacuo*. The crude material was then purified by flash column chromatography (2:3 *n*-Pent:EtOAc) to afford 2-((2-triethylsilyl)ethoxyl)methyl) phthalazin-1(*2H*)-one as a colorless oil (435 mg, 1.57 mmol, 46%).

^{1}H NMR (400 MHz, $CDCl_3$) δ = 8.41 (d, *J* = 7.8 Hz, 1H), 8.15 (s, 1H), 7.76 (dt, *J* = 21.7, 7.6 Hz, 2H), 7.67 (d, *J* = 7.7 Hz, 1H), 5.55 (s, 2H), 3.74 – 3.68 (m, 2H), 0.99 – 0.92 (m, 2H), - 0.05 (s, 9H); **^{13}C NMR** (100 MHz, $CDCl_3$) δ = 161.4, 139.6, 134.9, 133.2, 131.2, 129.4, 128.4, 127.6, 80.4, 68.5, 19.5, 0.00 (3C); **IR** (*v*, cm^{-1}): 1661, 1081, 833, 758; **HRMS** (ESI) for $C_{14}H_{20}N_2{}^{23}NaO_2{}^{28}Si$ $[M+Na]^+$ requires 299.1186 found 299.1185.

2-(4,4,5,5-Tetramethyl-1,3,2-dioxaborolan-2-yl)benzoic acid

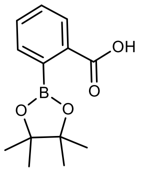

To a solution of anhydrous THF (20 mL) under an atmosphere of argon was added 2-boronobenzoic acid (1.00 g, 6.02 mmol), pinacol (0.71 g, 6.02 mmol) and magnesium sulfate (1.44 g, 12.04 mmol). The reaction was left to stir overnight at room temperature. Upon completion, the reaction was filtered and the excess solvent removed *in vacuo* affording 2-(4,4,5,5-Tetramethyl-1,3,2-dioxaborolan-2-yl)benzoic acid as a white solid (1.49 g, 6.00 mmol, 99%).

^{1}H NMR (400 MHz, DMSO-d$_6$) δ = 7.83 (dd, *J* = 7.6, 0.9 Hz, 1H), 7.55 (td, *J* = 7.3, 1.3 Hz, 1H), 7.46 (td, *J* = 7.5, 1.4, 1H), 7.43 – 7.39 (m, 1H), 1.29 (s, 12H); **^{13}C NMR** (100 MHz, DMSO-d$_6$) δ = 169.6, 134.7, 132.2, 131.9, 129.2, 128.3, 83.4 (2C), 25.0 (4C) (the carbon bearing the boron substituent is not observed); **IR** (*v,* cm^{-1}): 2972, 1679, 1344, 1304, 1141, 754; **HRMS** (ESI) for $C_{13}H_{16}{}^{10}BO_4$ [M-H]$^-$ requires 247.1212 found 247.1215; **Mp**: 118 – 120 °C.

(4-(Cyclopropanecarbonyl)piperazin-1-yl)(2-(4,4,5,5-tetramethyl-1,3,2-dioxaborolan-2-yl)phenyl)methanone

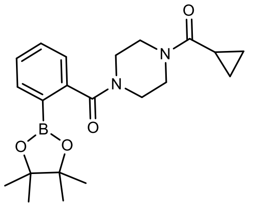

To a round bottom flask containing 2-(4,4,5,5-Tetramethyl-1,3,2-dioxaborolan-2-yl)benzoic acid (1.00 g, 4.03 mmol) was added DCM (40 mL), diisopropylethylamine (1.05 mL, 6.04 mmol) and HBTU (2.29 g, 6.04 mmol). The reaction mixture was stirred for 30 min before adding cyclopropylpiperazine-1-ylmethanone (0.86 mL, 6.04 mmol). The reaction was stirred overnight before the excess solvent with removed *in vacuo* and the crude material purified *via* flash column chromatography (EtOAc:MeOH 10:1) affording (4-(Cyclopropanecarbonyl)piperazin-1-yl)(2-(4,4,5,5-tetramethyl-1,3,2-dioxaborolan-2-yl)phenyl)methanone as a white solid (1.08 g, 2.82 mmol, 70%) in a mixture of rotamers (2:1). The shifts relating to the minor rotamer has been donated with an asterisk.*

^{1}H NMR (400 MHz, CDCl$_3$) δ = 7.81 (d, *J* = 7.4 Hz, 1H + 1H*), 7.45 (td, *J* = 7.6, 1.5 Hz, 1H + 1H*), 7.35 (td, *J* = 7.5, 1.3 Hz, 1H + 1H*), 7.22 (d, *J* = 7.5 Hz, 1H + 1H*), 3.86 – 3.69 (m, 4H, 4H*), 3.67 – 3.50 (m, 2H, 2H*), 3.34 – 3.07 (m, 2H, 2H*), 1.81 – 1.52 (m, 1H + 1H*), 1.29 (s, 12H + 12H*), 1.04 – 0.95 (m, 2H

+ 2H*), 0.67 – 0.87 (m, 2H + 2H*); **^{13}C NMR** (100 MHz, $CDCl_3$) δ = 172.3 (1C + 1C*), 171.3 (1C + 1C*), 142.3 (1C + 1C*), 135.7 (1C + 1C*), 131.2 (1C + 1C*), 128.2 (1C + 1C*), 125.4 (1C + 1C*), 84.1 (2C + 2C*), 47.3 (1C*), 47.0, 45.2 (1C*), 44.9, 41.9 (2C), 41.6 (2C*) 24.9 (4C + 4C*), 11.0 (1C + 1C*), 7.65 (2C + 2C*) (the carbon bearing the boron substituent is not observed); **IR** (*v,* cm^{-1}): 2970, 1634, 1467, 1213, 1014, 741; **HRMS** (ESI) for $C_{21}H_{30}{}^{10}BN_2O_4$ $[M+H]^+$ requires 384.2220 found 384.2218; **Mp**: 85 – 87 °C.

(4-(Cyclopropanecarbonyl)piperazin-1-yl)(2-fluorophenyl)methanone

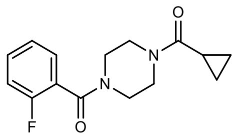

To a round bottom flask was added, 2-fluorobenzoic acid (1.00 g, 7.14 mmol), DCM (40 mL), diisopropylethylamine (1.86 mL, 10.7 mmol) and HBTU (4.06 g, 10.7 mmol). The reaction mixture was then stirred for 30 min at room temperature before adding cyclopropylpiperazine-1-ylmethanone (1.52 mL, 10.7 mmol). The reaction was stirred overnight before the excess solvent with removed *in vacuo* and the crude material purified *via* flash column chromatography (EtOAc:MeOH 10:1) affording (4-(Cyclopropanecarbonyl)piperazin-1-yl)(2-(4,4,5,5-tetramethyl-1,3,2-dioxaborolan-2-yl)phenyl)methanone as a white solid (1.32 g, 4.78 mmol 67%) in a mixture of rotamers (2:1). In each case the shift relating to the minor rotamer has been donated with an asterisk.*

^{1}H NMR (400 MHz, $CDCl_3$) δ = 7.40 – 7.29 (m, 2H + 2H*), 7.16 (td, *J* = 7.5, 1.1 Hz, 1H + 1H*), 7.04 (ddd, *J* = 9.4, 8.3, 1.1 Hz, 1H + 1H*), 3.88 – 3.46 (m, 6H + 6H*), 3.38 – 3.20 (m, 2H + 2H*), 1.76 – 1.58 (m, 1H + 1H*), 0.98 – 0.88 (m, 2H + 2H*), 0.83 – 0.62 (m, 2H + 2H*); **^{13}C NMR** (100 MHz, $CDCl_3$) δ = 172.3 (1C + 1C*), 165.4 (1C + 1C*), 158.0 (d, *J* = 247.7 Hz, 1C + 1C*), 131.7 (d, *J* = 8.0 Hz, 1C + 1C*), 129.1 (1C + 1C*), 124.9 (d, *J* = 3.5 Hz, 1C + 1C*), 123.5 (d, *J* = 17.4, 1C + 1C*), 115.8 (d, *J* = 21.3, 1C + 1C*), 47.0 (1C*), 46.7 (1C), 45.6 (1C*), 45.1 (1C), 42.2 (2C), 41.9 (1C*), 41.7 (1C*), 11.0 (1C + 1C*), 7.64 (2C + 2C*); **{^{1}H}^{19}F NMR** (376 MHz, $CDCl_3$) δ = - 115.0 (s, 1F*), - 115.1 (s, 1F); **IR** (*v,* cm^{-1}): 1630, 1460, 1219, 1004, 727; **HRMS** (ESI) for $C_{15}H_{18}{}^{19}FN_2O_2$ $[M+H]^+$ requires 277.1282 found 277.1285; **Mp**: 65 – 67 °C.

3-((4-oxo-3,4-dihydrophthalazin-1-yl)methyl)benzoic acid

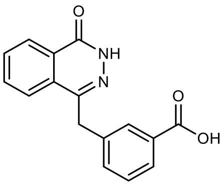

A solution of Dimethyl-(3-oxo-1,3-dihydrobenzofuran-1-yl)phosphonate (7.25 g, 30.0 mmol) and 3-formylbenzonitrile (2.62 g, 20.0 mmol) in THF (250 mL) was prepared at room temperature. The

solution was then cooled to 0 °C followed by the addition of Et_3N (6.10 mL, 30.0 mmol). The reaction mixture was warmed to room temperature and was stirred for 48 h, followed by concentration in vacuo to produce a white solid. The solid was suspended in water, collected by vacuum filtration and washed with hexane (2 × 20 mL) and Et_2O (3 × 20 mL) affording 3-((3-oxoisobenzofuran-1(3*H*)-ylidene)methyl)benzonitrile (4.40 g) as a crude white solid which was then taken through to the next step without further purification. 3-((3-oxoisobenzofuran-1(3*H*)-ylidene)methyl)benzonitrile (4.40 g, 17.8 mmol) was suspended in water (100 mL) and 13 M NaOH was added (26.4 mL, 238 mmol). The mixture was heated to 90 °C and stirred for 2 h, after which it was warmed to reflux (140 °C), followed by the addition of hydrazine monohydrate (7.48 mL, 240 mmol) and a further 16 h of stirring. The mixture was then cooled to room temperature and acidified with 5 M HCl to an approximate pH of 2. The solid precipitate was collected by vacuum filtration and washed with water (100 mL) and Et_2O (3 × 100 mL) affording 3-((4-oxo-3,4-dihydrophthalazin-1-yl)methyl)benzoic acid (4.18 g, 15.0 mmol, 50%, two steps) as a white solid.

^{1}H NMR (400 MHz, DMSO-d_6) δ = 12.64 (s, 1H), 8.25 (dd, *J* = 7.9, 1.4 Hz, 1H), 7.96 (d, *J* = 7.9 Hz, 1H), 7.91 – 7.85 (m, 2H), 7.82 (dd, *J* = 7.6, 1.2 Hz, 1H), 7.80 – 7.75 (m, 1H), 7.58 (dt, *J* = 7.7, 1.5 Hz, 1H), 7.42 (t, *J* = 7.7 Hz, 1H), 4.38 (s, 2H); **^{13}C NMR** (100 MHz, DMSO-d_6) δ = 167.6, 159.8, 145.5, 139.1, 134.0, 133.7, 132.0, 131.4, 129.8, 129.5, 129.3, 128.3, 127.9, 126.5, 126.0; **IR** (*v,* cm^{-1}): 3004, 1686, 1457, 1270, 1082, 937, 762, 666; **HRMS** (ESI) for $C_{16}H_{11}N_2O_3$ [M-H]$^-$ requires 279.0840 found 279.0647; **Mp**: > 250 °C.

4-(3-(4-(cyclopropanecabonyl)piperazine-1-carbnonyl)benzyl)phthalazin-1(*2H*)-one

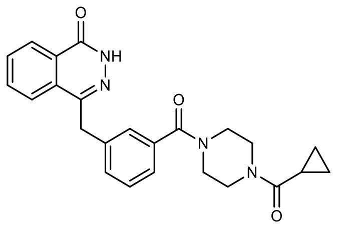

To a solution of 3-((4-oxo-3,4-dihydrophthalazin-1-yl)methyl)benzoic acid (1.00 g, 3.57 mmol) in DCM (100 mL) was added DIPEA (930 µL, 5.36 mmol) and HBTU (2.63 g, 5.36 mmol). The reaction mixture was stirred for 1 h before addition of cyclopropylpiperazine-1-ylmethanone (760 µL, 5.36 mmol) was carried out. The reaction mixture was then stirred at room temperature for 48 h, and the reaction mixture was extracted with DCM (3 × 50 mL) and washed with water (3 × 50 mL). The organic layers were collected, dried with $MgSO_4$ and the excess solvent removed *in vacuo*. Purification *via* reverse phase HLPC was then carried (Supplemental Figure 2) out affording 4-(3-(4-

(cyclopropanecabonyl)piperazine-1-carbnonyl)benzyl)phthalazin-1(*2H*)-one (810 mg, 55**%**) as a white solid.

^{1}H NMR (400 MHz, $CDCl_3$) δ = 11.83 (bs, 1H), 8.46 – 8.39 (m, 1H), 7.73 – 7.67 (m, 3H), 7.37 – 7.30 (m, 3H), 7.27 – 7.20 (m, 1H), 4.33 (s, 2H), 3.89 – 3.18 (m, 8H), 1.78 – 1.60 (m, 1H), 1.00 – 0.90 (m, 2H), 0.82 – 0.64 (m, 2H); **^{13}C NMR** (100 MHz, $CDCl_3$) δ = 172.4, 170.4, 161.1, 145.7, 138.3, 135.5, 133.5, 131.5, 130.2, 129.6, 129.0, 128.2, 127.2, 127.0, 125.5, 125.1, 47.5, 45.3, 42.3 (2C), 38.5, 14.2, 11.0, 7.73; **IR** (*v,* cm^{-1}): 2925, 1632, 1430, 1353, 1101, 772; **HRMS** (ESI) for $C_{24}H_{25}N_4O_3$ $[M+H]^+$ requires 417.1856 found 417.2001; **Mp**: 82 – 84 °C.

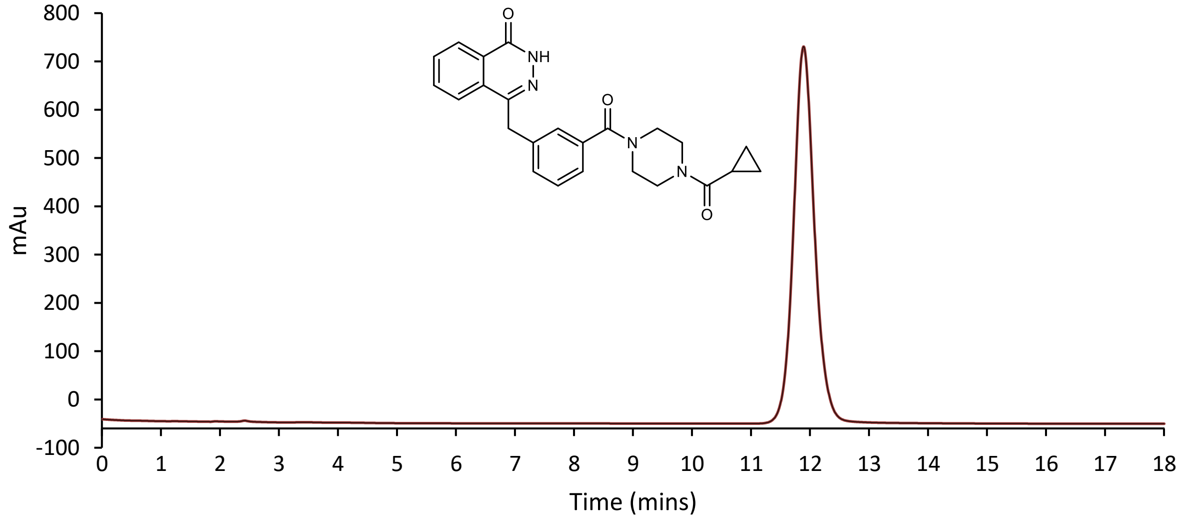

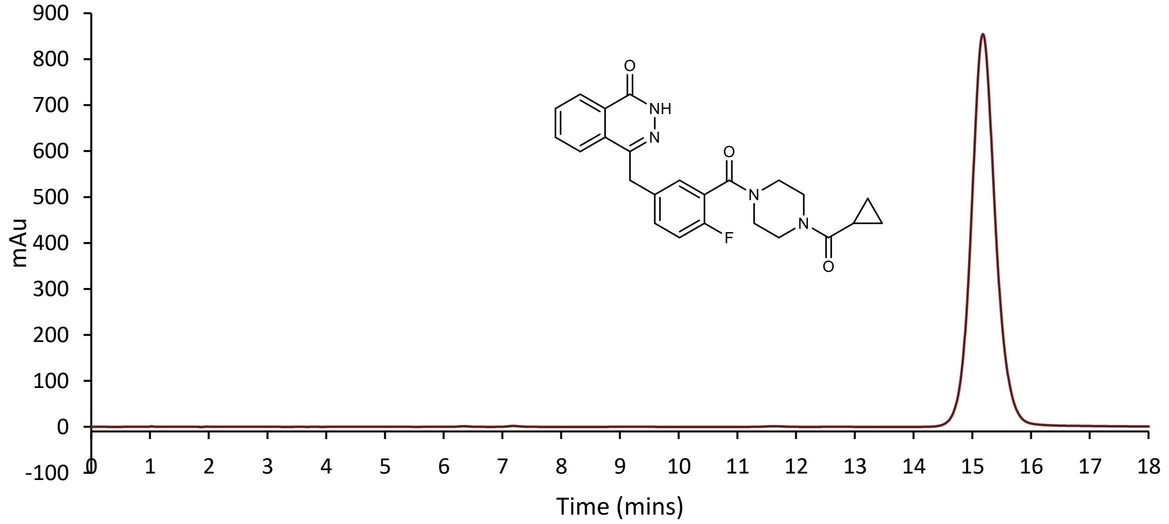

Supplemental Figure 2. *HPLC UV Trace of 4-(3-(4-(cyclopropanecabonyl)piperazine-1-carbnonyl)benzyl)phthalazin-1(2H)-one and olaparib. HPLC Eluent: Synergi 4 µm Hydro-RP 80A, 150 x 4.6 mm with 25% MeCN/75% H_2O (isocratic 1 mL/min) monitoring with UV (220 nm).*

Synthesis of [Cu(OTf)$_2$(impy)$_4$] complex

A solution of imidazo[1,2-b]pyridazine (impy) (758 mg, 6.36 mmol, 10 equiv.) in MeOH (1 mL) was added dropwise at 55°C to a solution of Cu(OTf)$_2$ (230 mg, 0.636 mmol, 1.0 equiv.) in MeOH (1 mL). The blue precipitate which formed was washed with Et_2O (3 x 2 mL), then recrystallized from hot MeOH to afford [Cu(OTf)$_2$(impy)$_4$] (324 mg, 0.387 mmol, 61%).

Anal. Calcd. for $C_{26}H_{20}CuF_6N_{12}O_6S_2$: C, 37.26; H, 2.41; N, 20.05. Found: C, 37.07; H, 2.33; N, 19.91; **IR** (ATR, neat): ν (cm^{-1}) = 2981, 1620, 1541, 1503, 1374, 1352, 1306, 1281, 1241, 1221, 1149, 1071, 1027, 950, 918, 879, 801, 755, 733, 632.

2. Radiochemical synthesis

Radiochemistry:

[^{18}F]Fluoride was produced by Alliance Medical (UK) via the $^{18}O(p,n)^{18}F$ reaction and delivered as [^{18}F]fluoride in ^{18}O-enriched-water. Radiosynthesis and azeotropic drying was performed on a NanoTek microfluidic device (Advion).

Procedure for preparation of a solution of [^{18}F]KF/K$_{222}$ in MeCN:

A solution of Kryptofix 222 (15 mg) and K_2CO_3 (3 mg) in 1 mL of MeCN/H_2O, 4:1 was freshly prepared. [^{18}F]Fluoride (3.0-4.0 GBq) was separated from ^{18}O-enriched-water using a Chromafix PSHCO3 ^{18}F separation cartridge (45 mg) and subsequently released with 900 µL (in 6 x 150 µL portions) of the K_{222}/K_2CO_3 solution into a 5 mL V-vial containing a magnetic stir bar in the concentrator. The solution was dried with five cycles of azeotropic drying with MeCN (5 x 200 µl) under a flow of N_2 at 105°C. The dried [^{18}F]KF/K$_{222}$ residue was redissolved in anhydrous MeCN (500 - 1000 µL).

General procedure for small scale heterocycle screening experiments with 4-(4,4,5,5-tetramethyl-1,3,2-dioxaborolan-2-yl)benzonitrile:

A solution of [^{18}F]KF/K$_{222}$ in MeCN (20 - 30 MBq, 10-50 µL) was dispensed into a V-vial containing Cu(OTf)$_2$(py)$_4$ (0.0053 mmol), 4-(4,4,5,5-tetramethyl-1,3,2-dioxaborolan-2-yl)benzonitrile (13.7 mg, 0.06 mmol) and a magnetic stirrer bar. Air (20 mL) was flushed through the reaction vial using a syringe and then a solution containing the heterocycle (0.06 mmol) in DMF (300 µL) was added via syringe. The sealed vial was heated at 110 °C for 20 min. The reaction was quenched by addition of water (200 µL). An aliquot was removed for analysis by radioTLC and HPLC for radiochemical conversion and product identity. Analysis was performed using a Waters Nova-Pak C18 column (4 µm, 3.9 x 150 mm) at a flow rate 1 mL/min. Radio-TLC was performed on Merck Kiesegel 60 F254 plates, using n-hexane/EtOAc (1:1) as eluent. Analysis was performed using a plastic scintillator/PMT detector.

General procedure for small scale ^{18}F-labeling of arenes:

A solution of [^{18}F]KF/K_{222} in MeCN (20 - 30 MBq, 10 - 50 µL) was dispensed into a V-vial containing Copper source (0.03 mmol), aryl pinacol boronate (0.02 mmol) and a magnetic stirrer bar. Air (20 mL) was flushed through the reaction vial using a syringe and then solvent (300 µL) was added via syringe. The sealed vial was heated at 110 °C for 20 min. The reaction was quenched by addition of water (200 µL). An aliquot was removed for analysis by radioTLC and HPLC for radiochemical conversion and product identity. Analysis was performed using the gradient given below with a Waters Nova-Pak C18 column (4 µm, 3.9 x 150 mm) at a flow rate 1 mL/min. Radio-TLC was performed on Merck Kiesegel 60 F254 plates, using as eluent DCM/MeOH (9:1). Analysis was performed using a plastic scintillator/PMT detector.

HPLC gradient for small scale heterocycle screening experiments and small scale ^{18}F-labeling of arenes:

Water/MeCN, 1 mL/min, Waters Nova-Pak C18 Column, 4 µm, 3.9 x 150 mm 0 - 1 min (5% MeCN) isocratic 1 - 10 min (5% MeCN to 95% MeCN) linear increase 10 - 14 min (95% MeCN) isocratic 14 - 15 min (95% MeCN to 5% MeCN) linear decrease 15 - 17 min (5% MeCN) isocratic.

Procedure for the Synthesis and Isolation of ^{18}F-Olaparib:

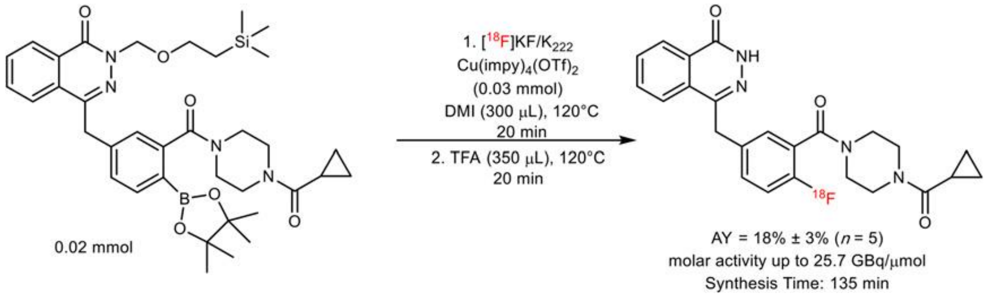

^{18}F-olaparib was obtained *via* the Cu-mediated ^{18}F-fluorodeboronation of the corresponding boronic ester precursor (Figure 1, 2), using methodology previously described by Tredwell *et al. (6)* [^{18}F]Fluoride was separated from ^{18}O-enriched-water using an anion exchange cartridge (Sep-Pak Accell Plus QMA Carbonate Plus Light Cartridge, 46 mg Sorbent per Cartridge, 40 µm particle size, Waters) and released with 900 µL (in 6 x 150 µL portions) of a solution of $K_{222}/K_2C_2O_4/K_2CO_3$ (kryptofix 222 (6.3 mg), $K_2C_2O_4$ (1 mg) and K_2CO_3 (0.1 mg) in 1 mL of MeCN/H_2O, 4:1) into a 5 mL V-vial containing a magnetic stir bar in the concentrator. The solution was dried with five cycles of azeotropic drying with MeCN (5 x 200 µL) under a flow of N_2 at 105°C. The 5 mL vial containing the dried [^{18}F]KF/$K_{2.2.2}$ complex was purged with 30 mL of air using a syringe and then a solution of arylboronate precursor (13.4 mg, 0.02 mmol) and $Cu(OTf)_2(impy)_4$ (25 mg, 0.03 mmol) in anhydrous 1,3-dimethyl-2-

imidazolidinone (DMI) (300 µL) was added. The mixture was heated at 120 °C for 20 min in a sealed vial with stirring. After 20 min, TFA (350 µL) was added and stirring was continued at 120 °C for 20 min. The reaction was then cooled to room temperature before quenching with H_2O (6 mL) and eluting over an Oasis HLB Plus cartridge (preconditioned with 2 mL MeOH and 10 mL H_2O). The vial was then rinsed with CH_3CN:H_2O (1:9, 2.0 mL), and eluting over the Oasis HLB cartridge after which the product was eluted with CH_3CN (2.0 mL) into a 5 mL V-vial. The MeCN was evaporated under a flow of N_2 at 100 °C until approximately 50 µL remained. The reaction mixture was then diluted in with 28% MeCN/ 72% 25 mM NH_4HCO_2 in H_2O and loaded directly onto a 2 mL HPLC loop and injected on a semi-Prep HPLC column (Synergi 4µm Hydro-RP 250x10mm) and eluted into a collection vial with 28% MeCN/ 72% 25 mM NH_4HCO_2 in H_2O monitoring with UV (254 nm) and radioactive traces.

The ^{18}F-olaparib was collected in 10 mL of H_2O and eluted over an Oasis HLB Plus cartridge (preconditioned with 2 mL MeOH and 10 mL H_2O). The cartridge was washed with H_2O (1.0 mL), after which the product was eluted with EtOH (2.0 mL). The ethanol was evaporated under a flow of N_2 while heating at 100 °C. The dry residue was then re-dissolved in 10% DMSO/PBS (pH = 7.4).

The Molar Activity of ^{18}F-olaparib was assessed by radio-HPLC, using an analytical Synergi 4 µm Hydro-RP 80A column, 150 x 4.6 mm eluted with 25% MeCN/75% H_2O (isocratic 1 mL/min), monitoring with UV (220 nm) and radioactive traces.

Test reactions were carried out on components of the molecule, to explore the compatibility of all chemical motifs with the Cu-assisted radiolabelling reaction (Supplemental Figure 3, 4, 5, 6). Additional optimisation of the Cu-complex catalyst and drying methods was performed to improve activity yields (Supplemental Table 1, 2). Molar activity was calculated based on a standard series and co-injection of cold, unlabelled olaparib (Supplemental Figure 7, Supplemental Table 3).

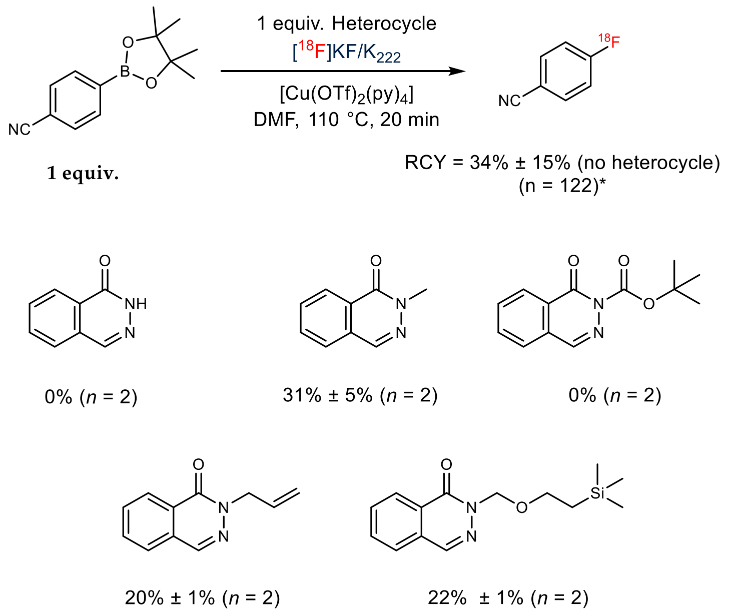

Supplemental Figure 3: Screening of Protecting Groups: *See reference (*7*)

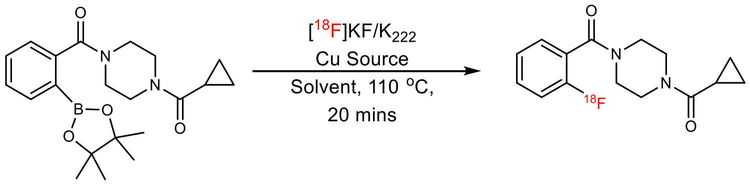

Supplemental Figure 4: Radiochemical conversion of (4-(Cyclopropanecarbonyl) piperazin-1-yl)(2-(4,4,5,5-tetramethyl-1,3,2-dioxaborolan-2-yl)phenyl)methanone

Supplemental Table 1. Radiochemical conversion of (4-(Cyclopropanecarbonyl)piperazin-1-yl)(2-(4,4,5,5-tetramethyl-1,3,2-dioxaborolan-2-yl)phenyl)methanone

**Cu Source**	**Solvent (400 µL)**	**RCC (*n* = 2)**
$Cu(OTf)_2(py)_4$	DMA	17% ± 3%
$Cu(OTf)_2(impy)_4$	DMA	48% ± 1%
$Cu(OTf)_2(impy)_4$	DMI	82% ± 1%

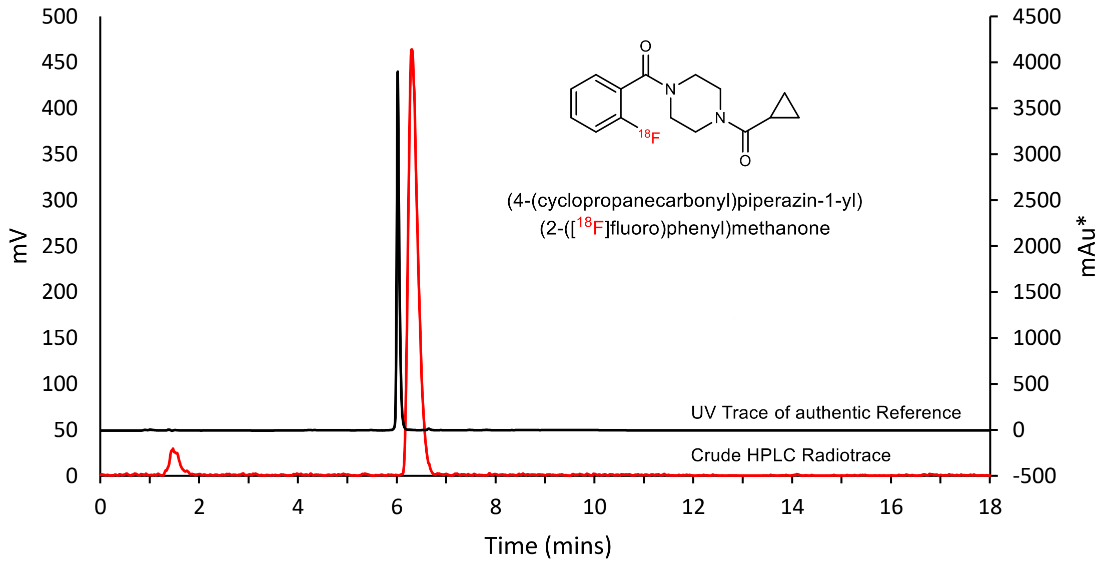

Supplemental Figure 5. UV Trace and Radiotrace of (4-(Cyclopropanecarbonyl)piperazin-1-yl)(2-fluorophenyl)methanone

Supplemental Table 2: ^{18}F-Olaparib Isolation Results:

Run	QMA Ion	Activity (MBq)				RCY (%)[b]	Synthesis Time (min)	Molar Activity (GBqµmol^{-1})
		Starting	Dried	HLB 1	HLB 2			
1	Oxalate[a]	5500	3070	1050	290	5	149	7.2
2	Oxalate[a]	3620	2650	1076	550	15	136	10.1
3	Oxalate[a]	3400	1502	711	220	6	154	2.8
4	Oxalate[a]	7750	3120	1215	309	4	135	9.8
5	Oxalate[a]	8080	3140	1152	470	6	135	21.3
			**Activity Yield[c]: 7% ± 4% (*n* = 5)**					
6	Carbonate	8510	7420	3890	1168	14	140	10.9
7	Carbonate	10000	8420	4330	2300	23	130	21.3
8	Carbonate	10000	8950	3510	1836	18	129	25.7
9	Carbonate	1510	1281	640	278	18	144	2.7
10	Carbonate	7780	6730	3100	1343	17	132	15.4
			**Activity Yield[d]: 18% ± 3% (*n* = 5)**					

[a]Pre-conditioned with 3 mL of a 10 mg/ml $K_2C_2O_{4(aq)}$ solution followed by 5 mL H_2O at a flow rate of 3 mL/min; [b]non-decay corrected; [c]AY under oxalate drying conditions; [d]AY under carbonate drying conditions.

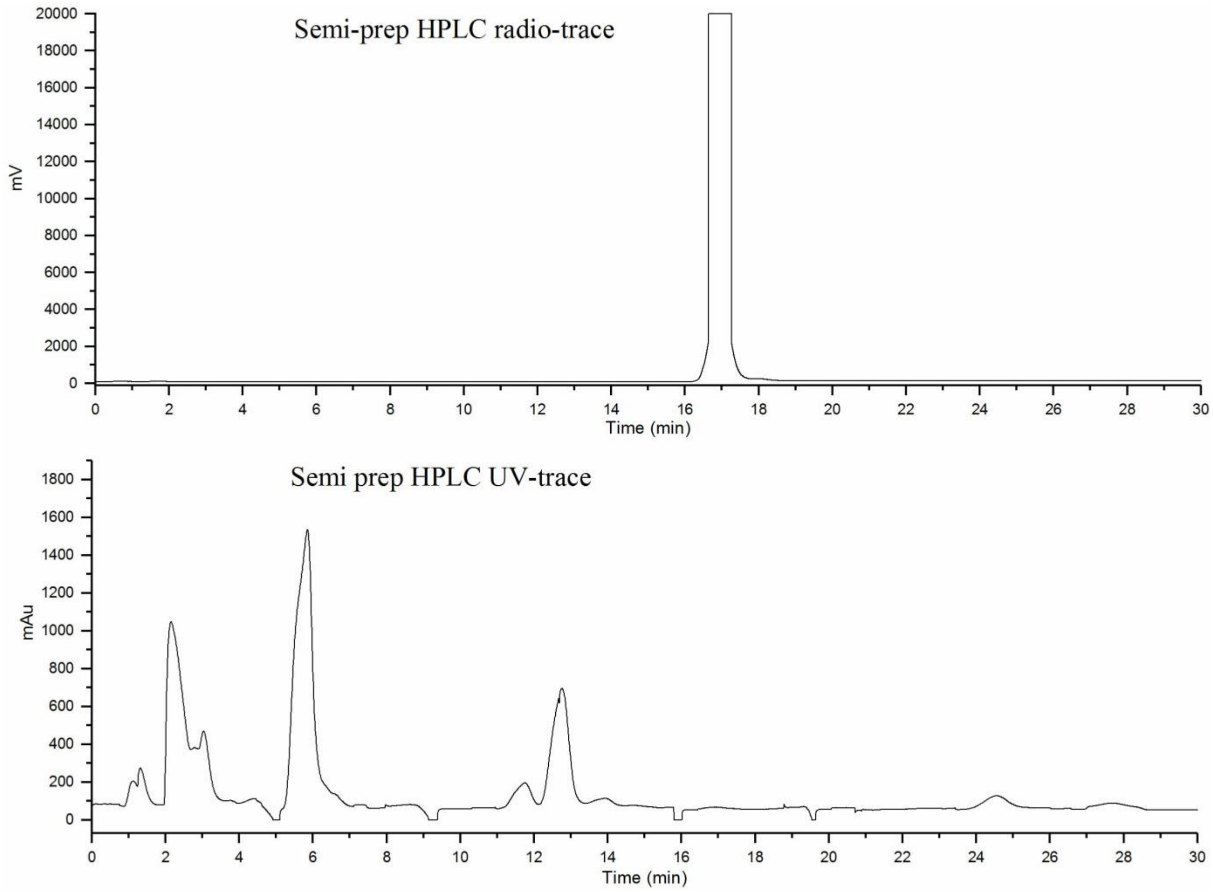

Supplemental Figure 6. Semi-prep radioHPLC purification of ^{18}F-olaparib

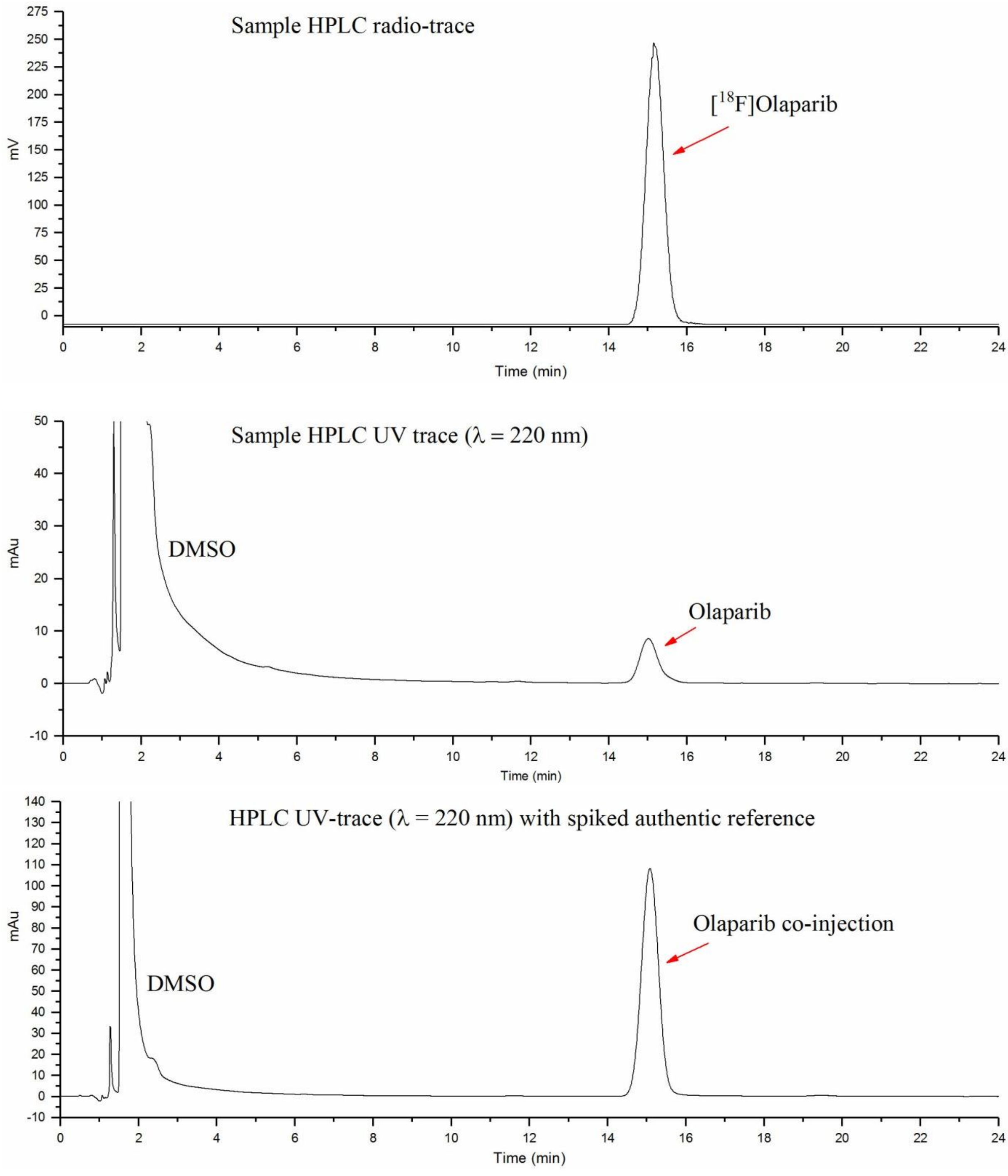

Supplemental Figure 7. Purified ^{18}F-olaparib was injected onto an analytical column. Additionally, a sample spiked with an authentic reference sample of olaparib (0.7 µg) was analyzed. Analytical HPLC conditions are listed in the previous section.

Supplemental Data: Molar Activity of ^{18}F-olaparib:

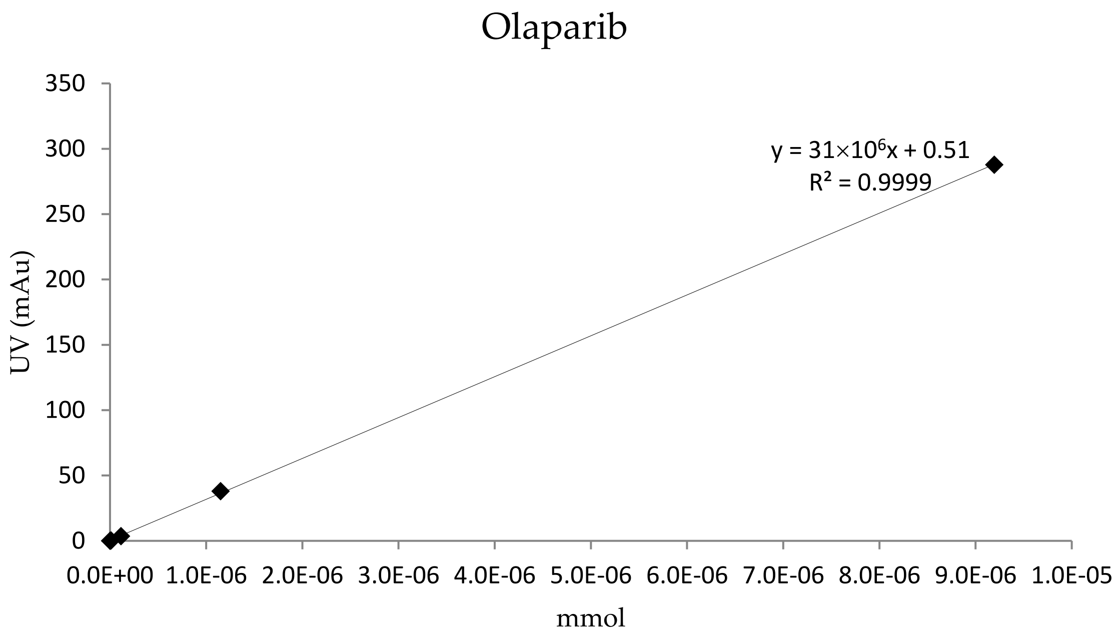

Supplemental Table 3. Molar Activity Calculations for ^{18}F-olaparib [a]Under oxalate drying conditions; [b]Under carbonate drying conditions.

Injection Number	Activity (MBq)	Area (mAu)	Mmol injected ($*10^{-7}$)	Molar Activity ($GBq.\mu mol^{-1}$)
1[a]	2.0	9.16	2.7	7.2
2[a]	1.4	4.82	1.3	10.4
3[a]	1.6	18.29	5.6	2.8
4[a]	0.7	2.73	0.71	9.8
5[a]	3.1	5.06	1.4	21.3
6[b]	1.1	3.67	1.0	10.9
7[b]	1.6	2.86	0.75	21.3
8[b]	1.7	2.58	0.66	25.7
9[b]	2.2	25.79	8.1	2.7
10[b]	2.6	5.79	1.7	15.4

2. NMR Spectra of Novel Compounds:

2-Bromo-5-formylbenzonitrile

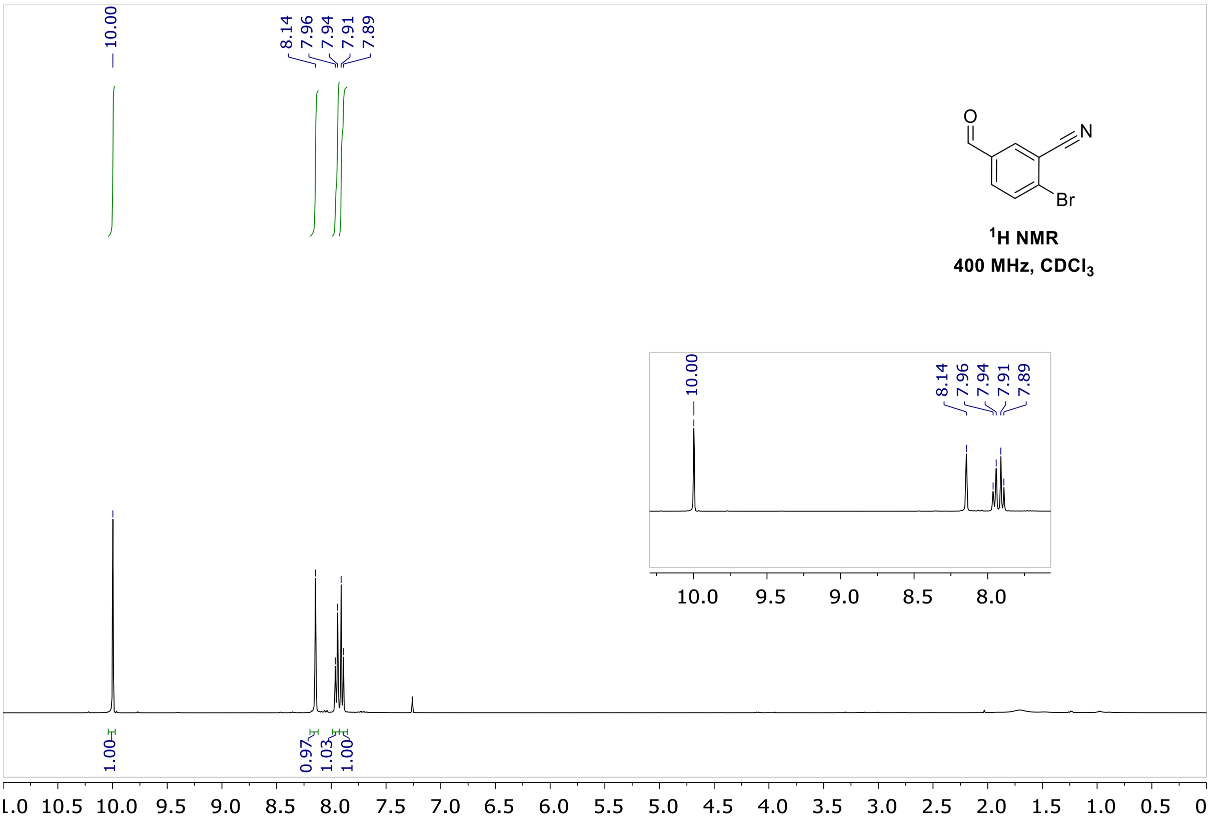

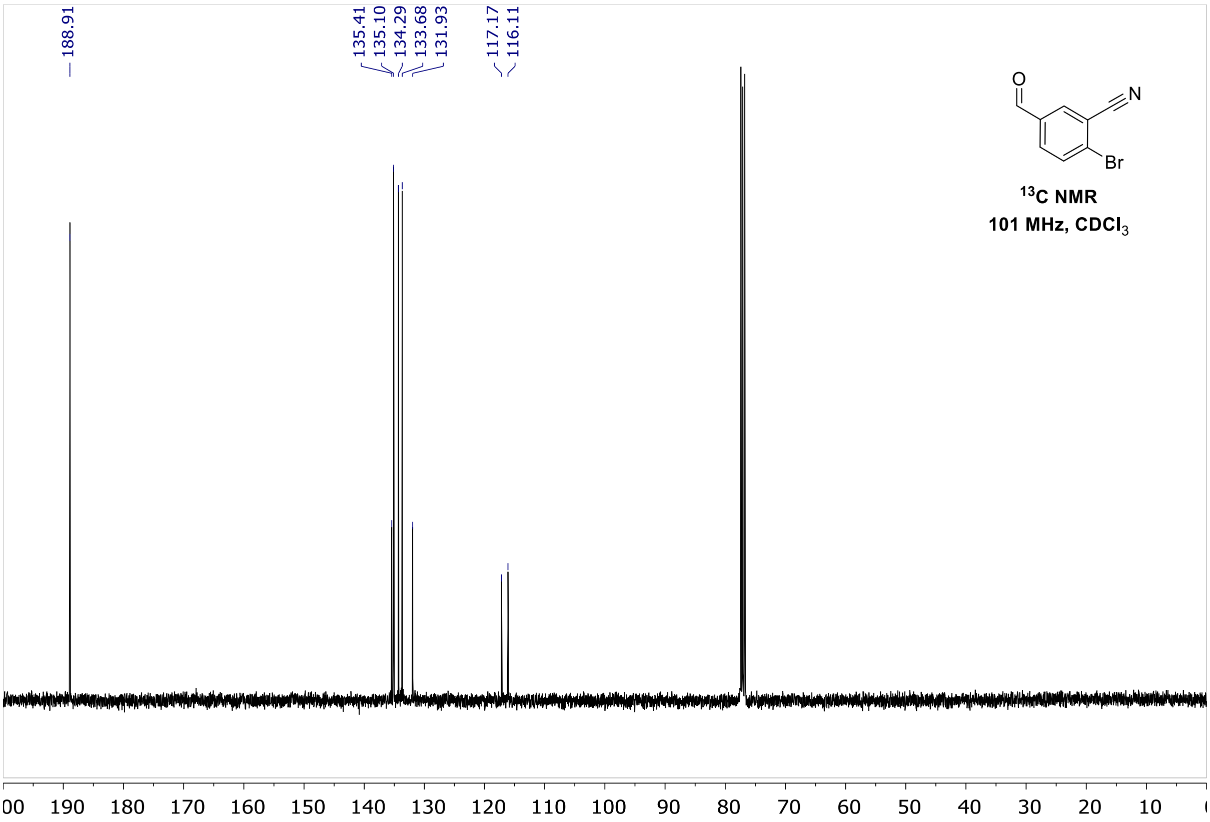

2-Bromo-5-((3-oxoisobenzofuran-1(*3H*)-ylidene)methyl)benzonitrile

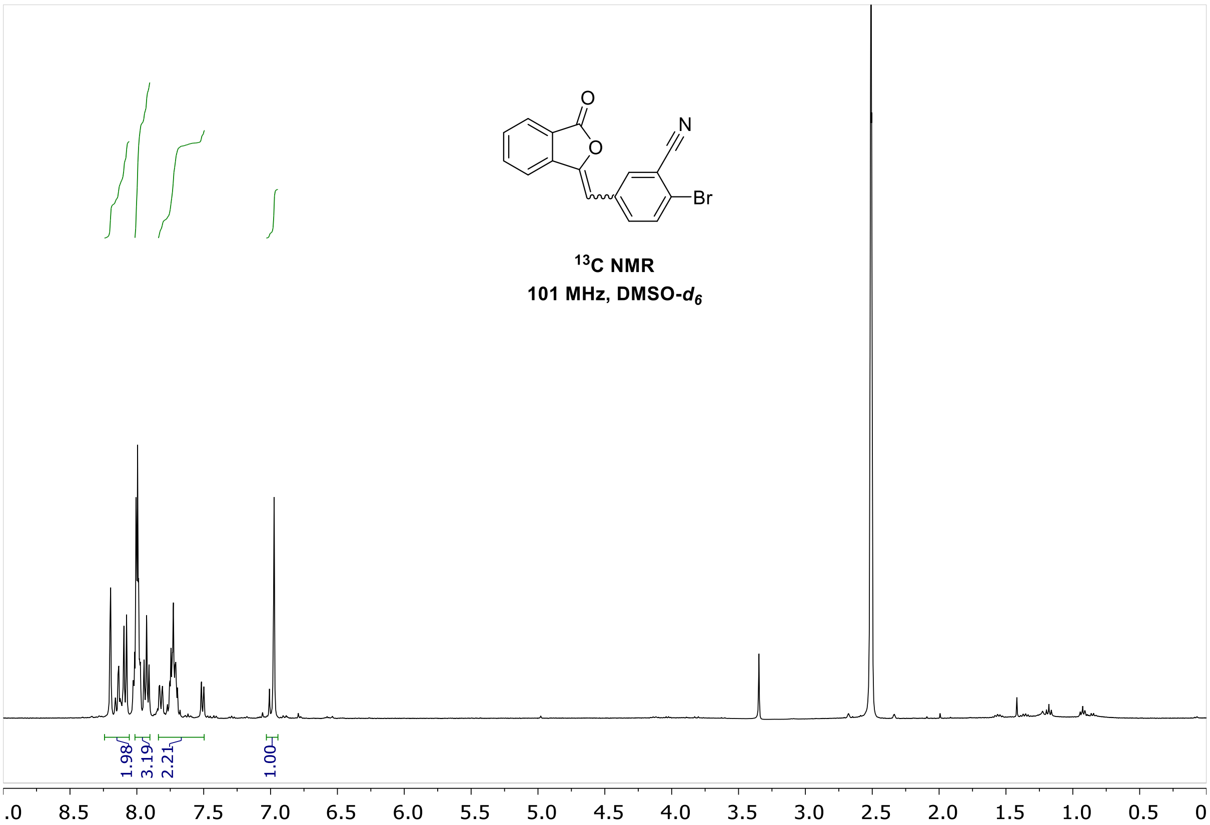

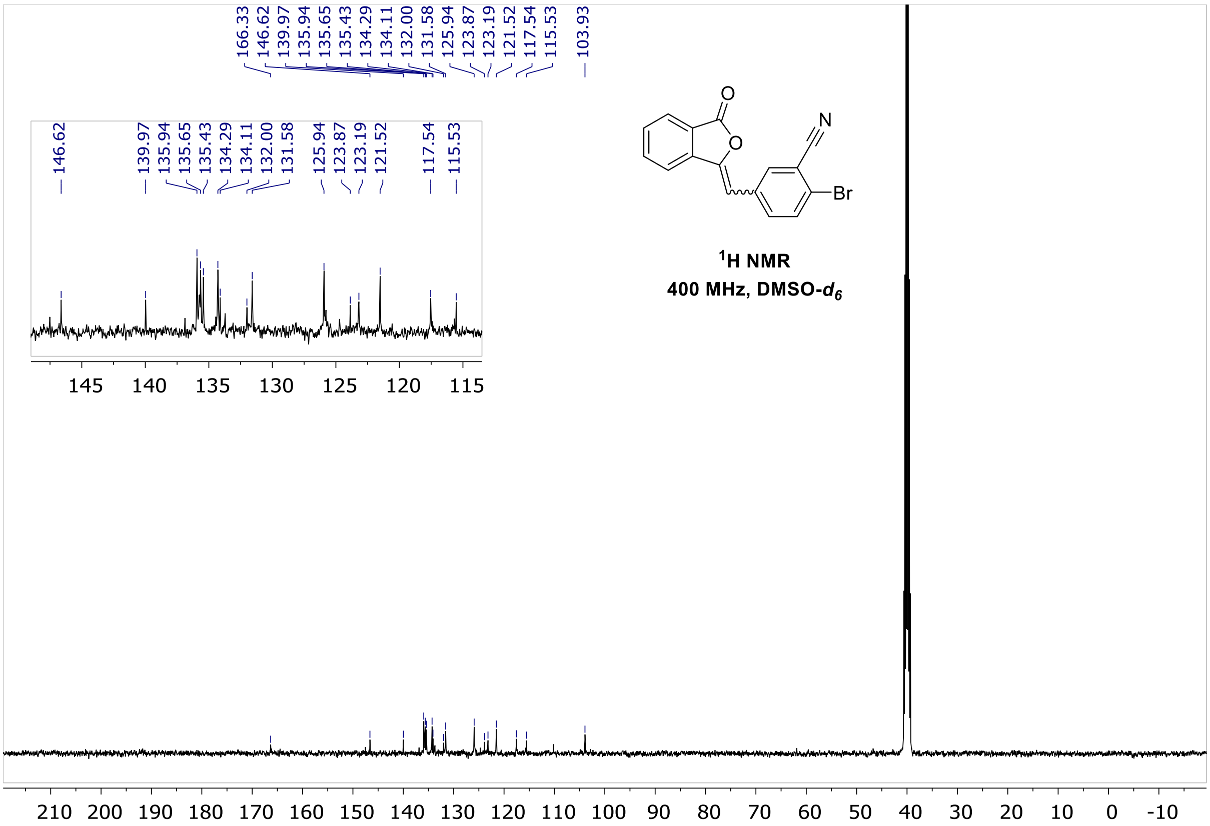

2-Bromo-5-((4-oxo-3,4-dihydrophthalazin-1-yl)methyl)benzoic acid

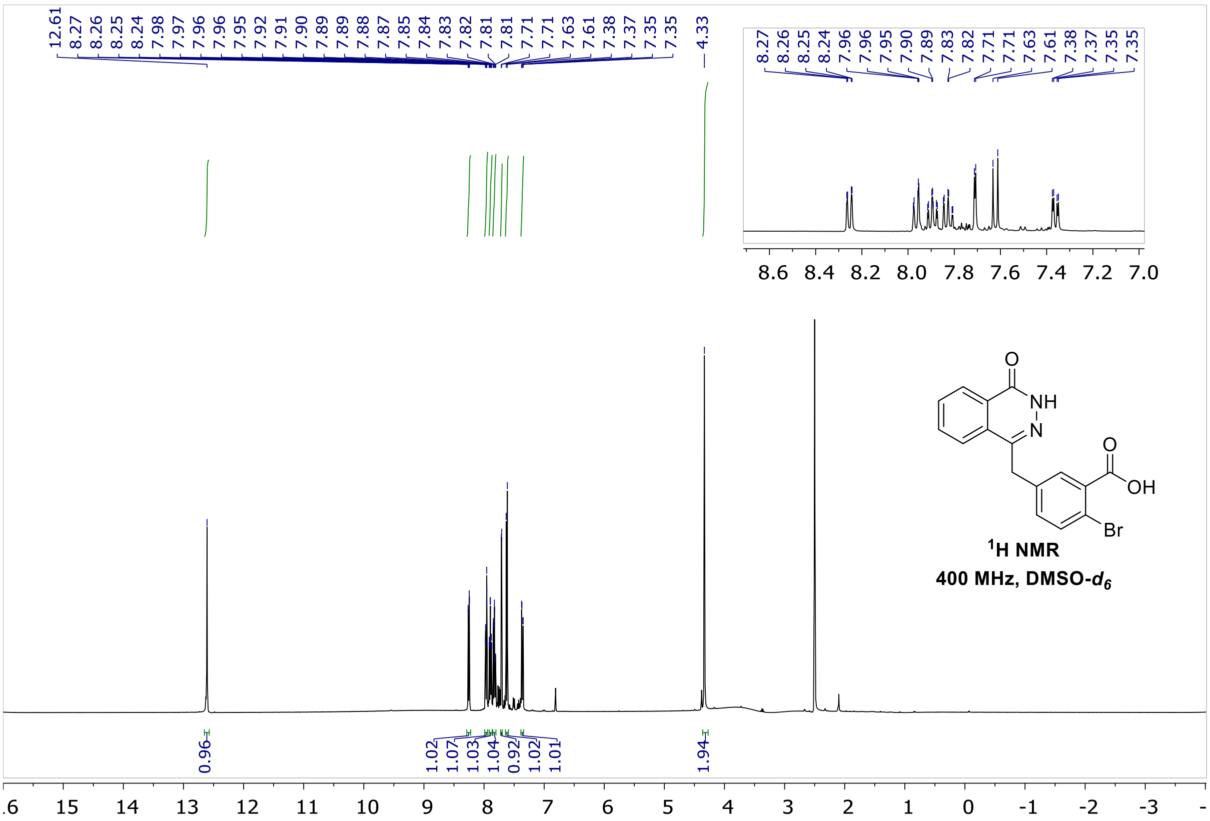

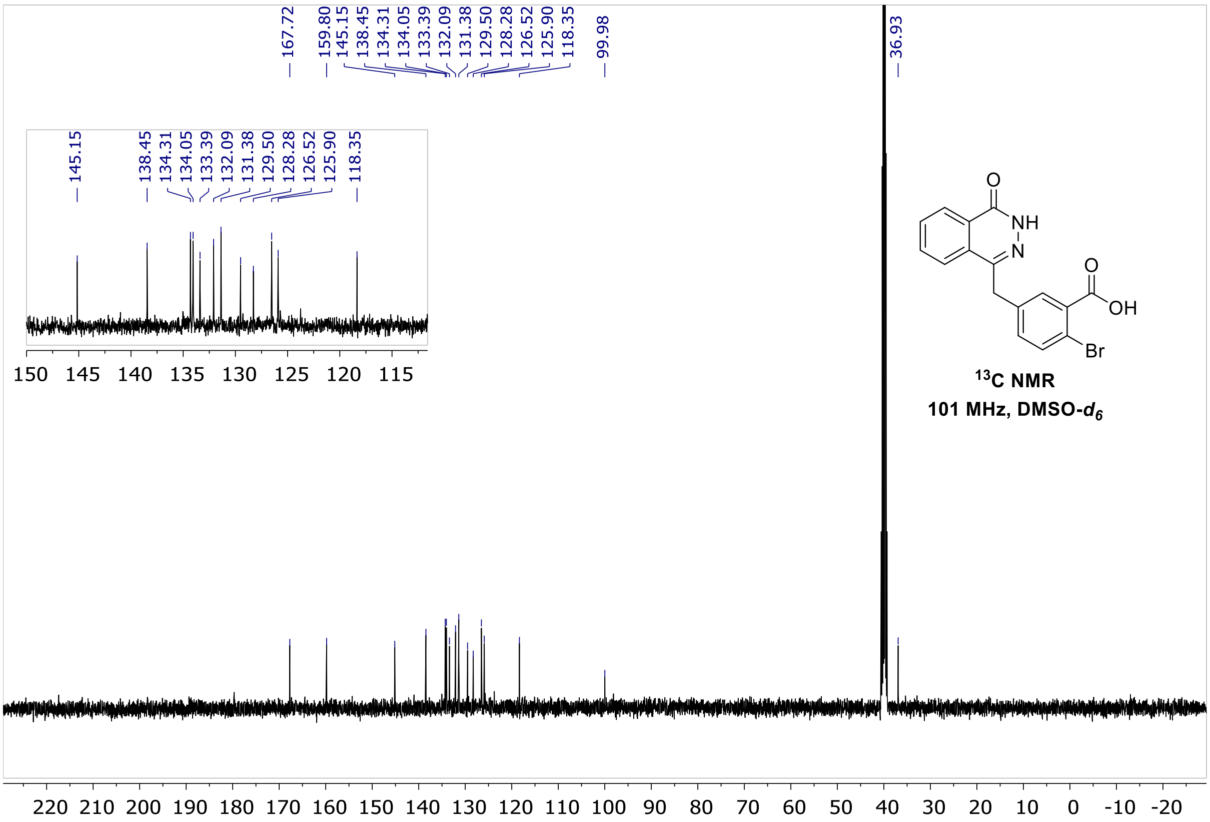

Methyl-2-bromo-5-((4-oxo-3,4-dihydrophthalazin-1-yl)methyl)benzoate

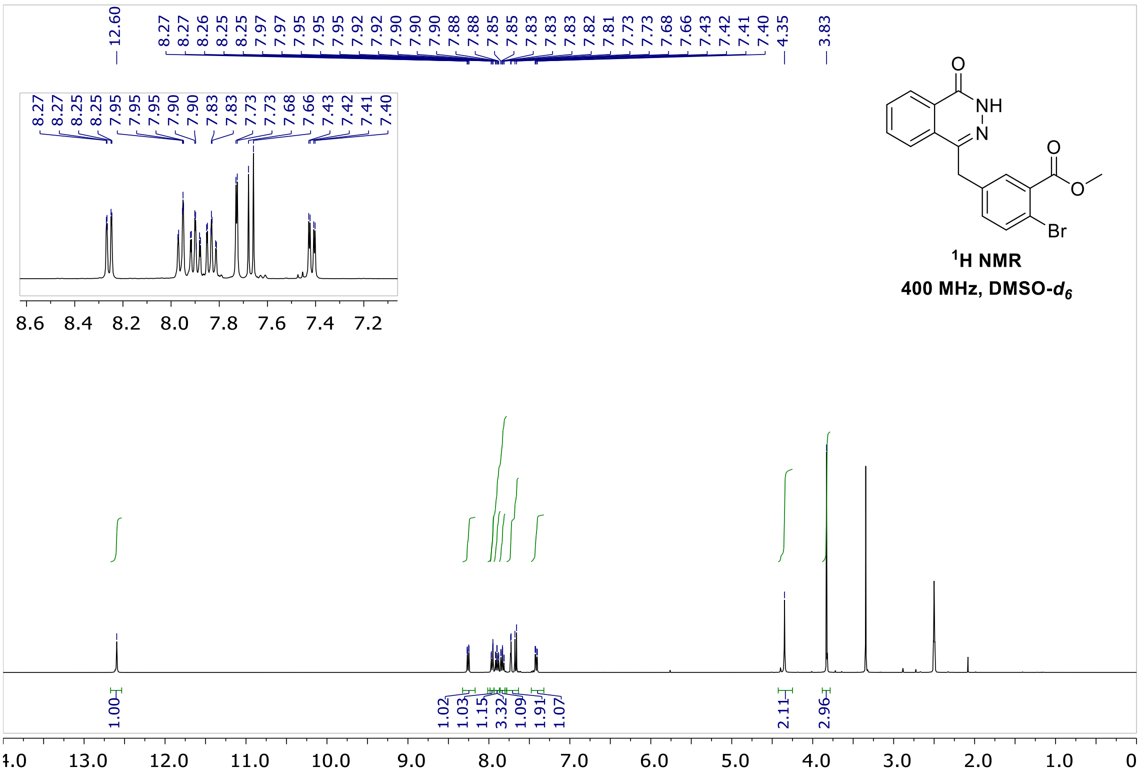

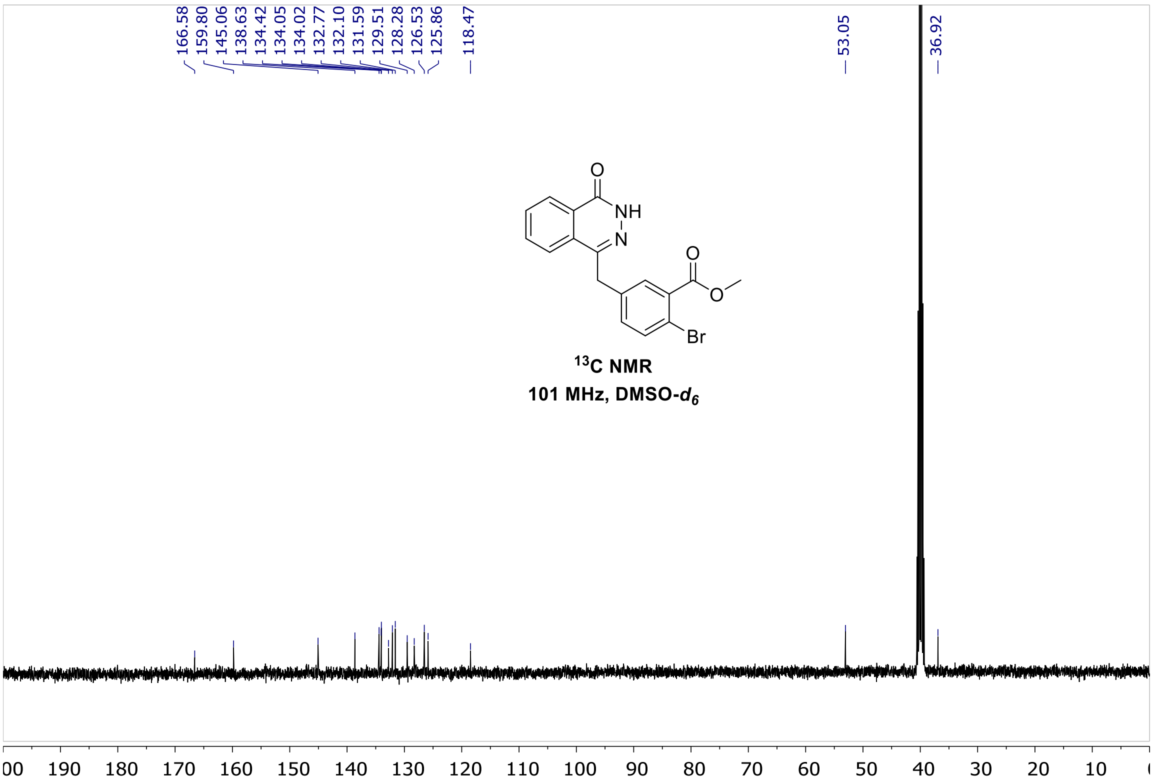

Methyl-2-bromo-5-((4-oxo-((2-(trimethylzilyl)ethoxy)methyl)-3,4-dihydro phthalazin-1-yl) methyl)benzoate

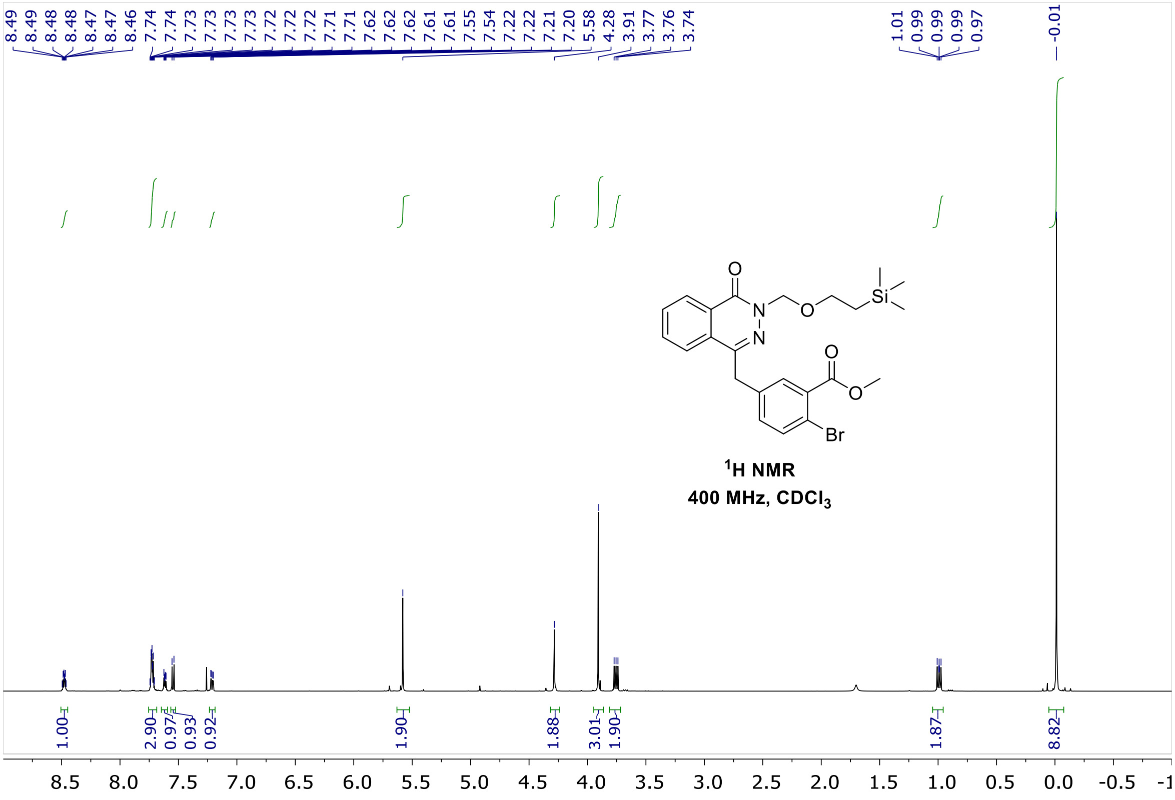

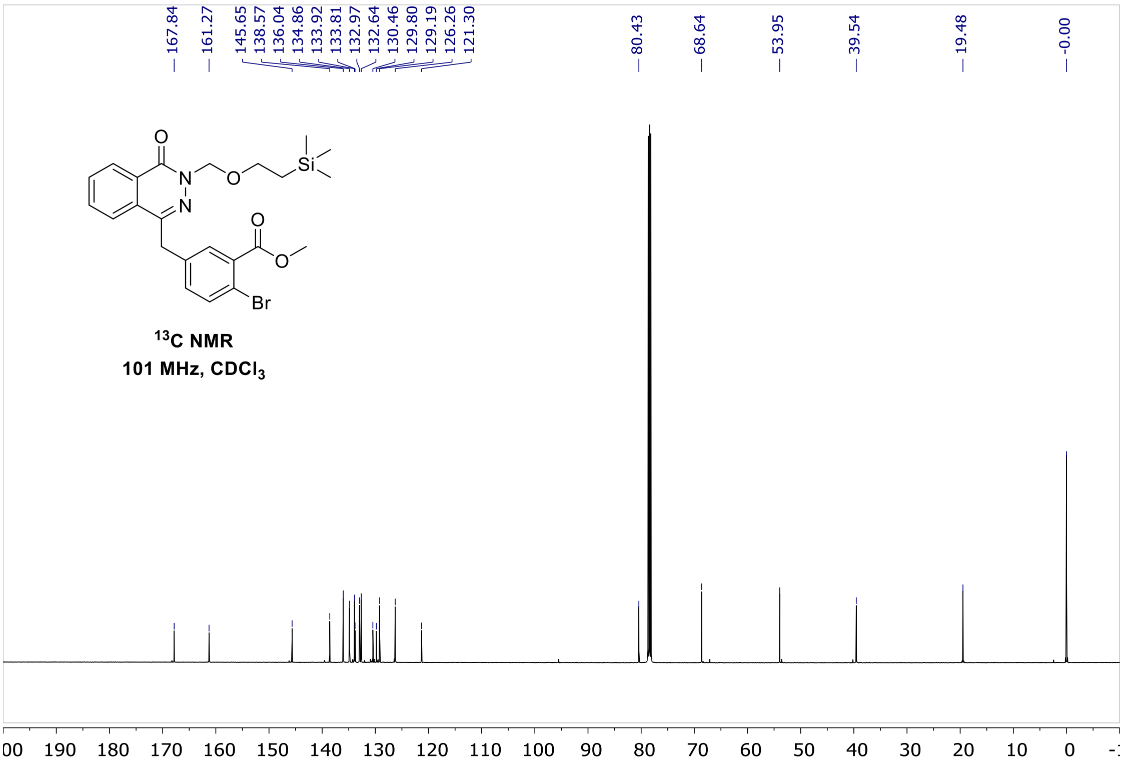

4-(3-(4-(cyclopropanecarbonyl)piperazine-1-carbonyl)-4-(4,4,5,5-tetramethyl-1,3,2-dioxaborolan-2-yl)benzyl)-2-((2-trimethlsilyl)ethoxy)methyl)phthalazin-1(2*H*)-one

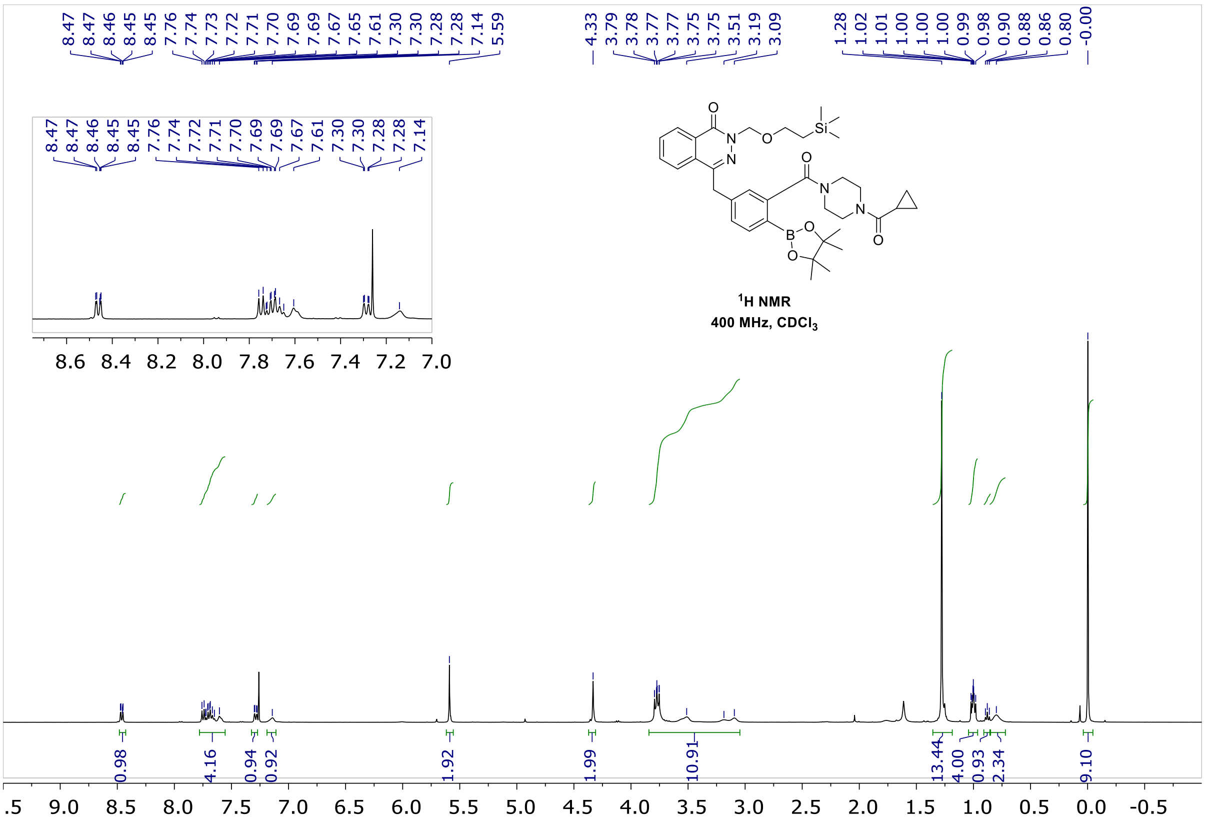

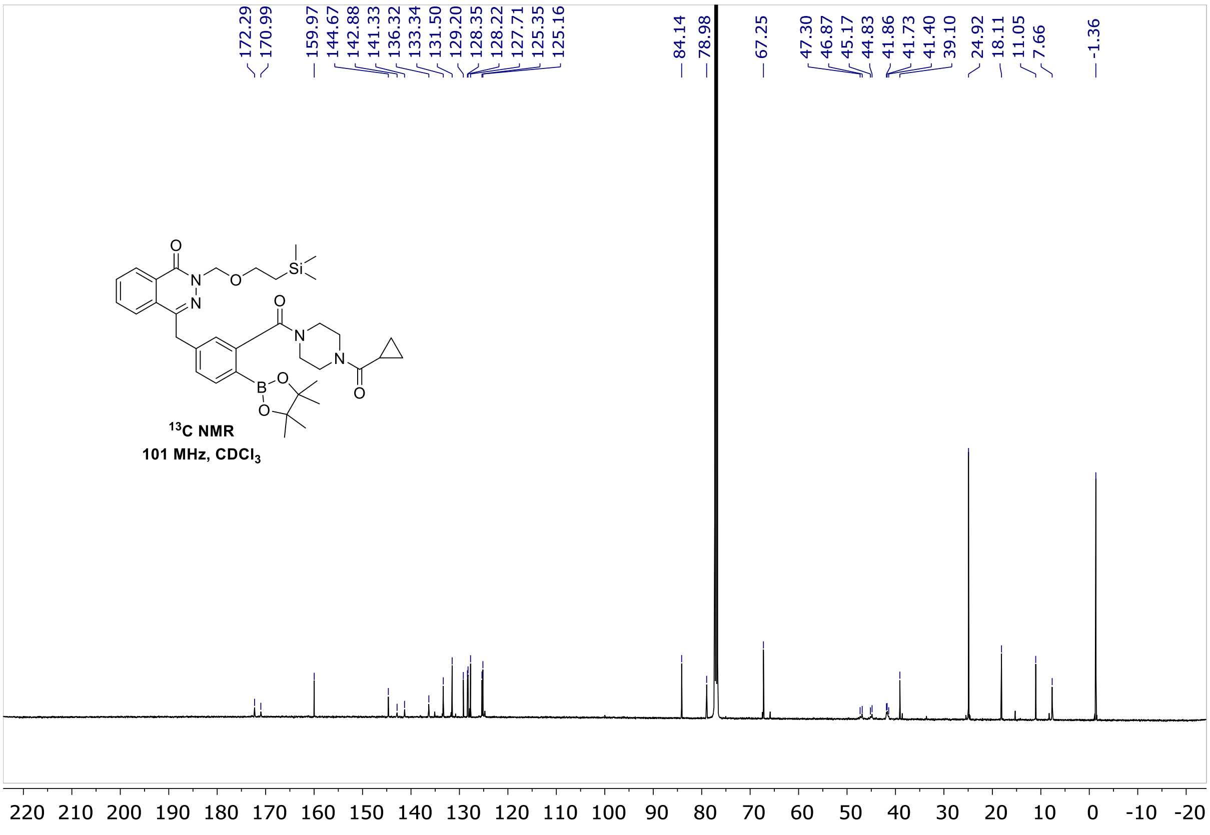

tert-Butyl 1-oxophthalazine-2(1H)-carboxylate

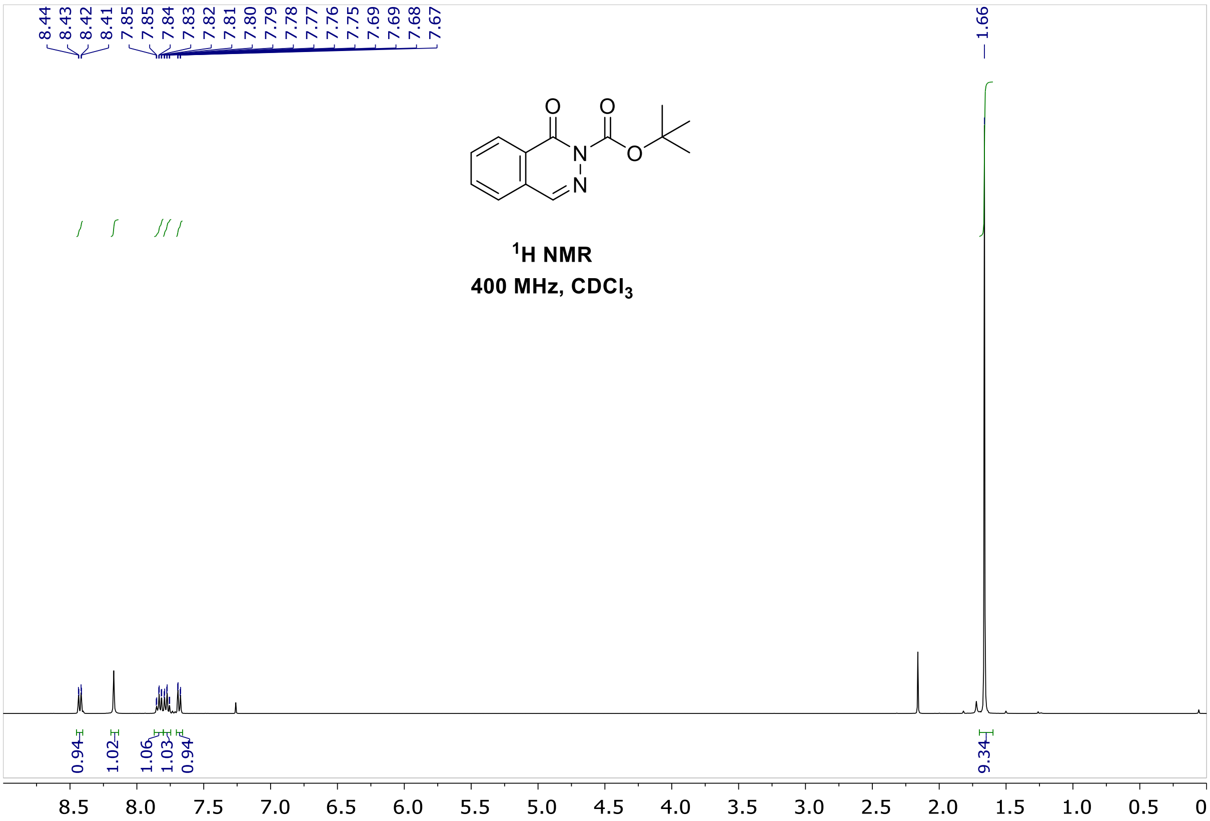

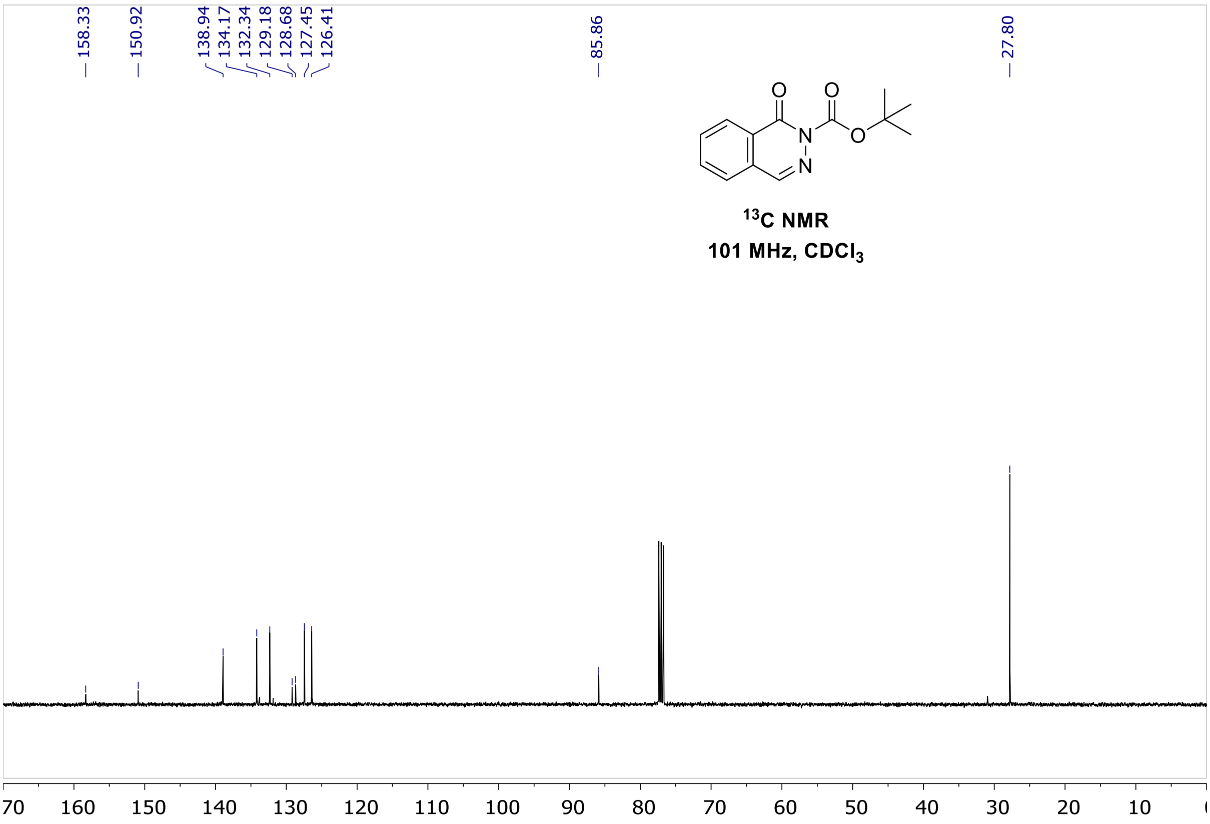

2-((2-triethylsilyl)ethoxyl)methyl)phthalazin-1(2*H*)-one

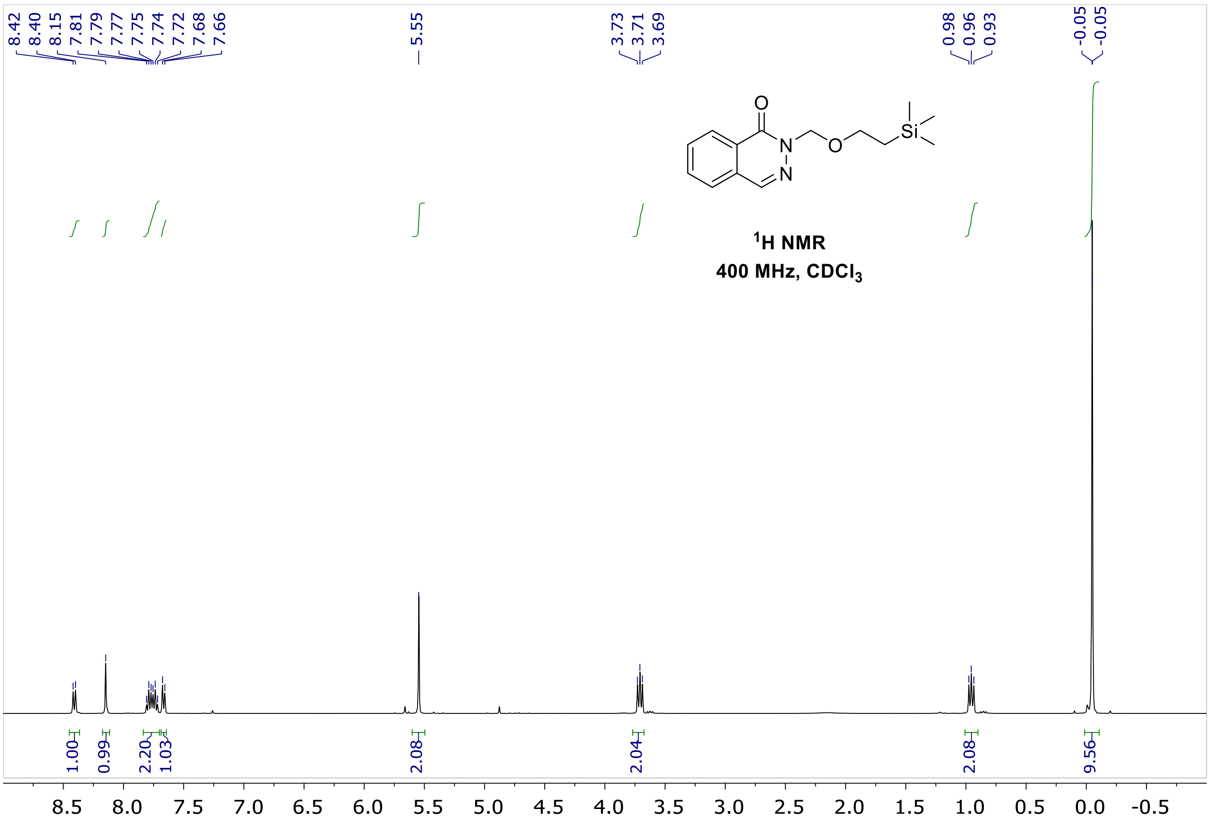

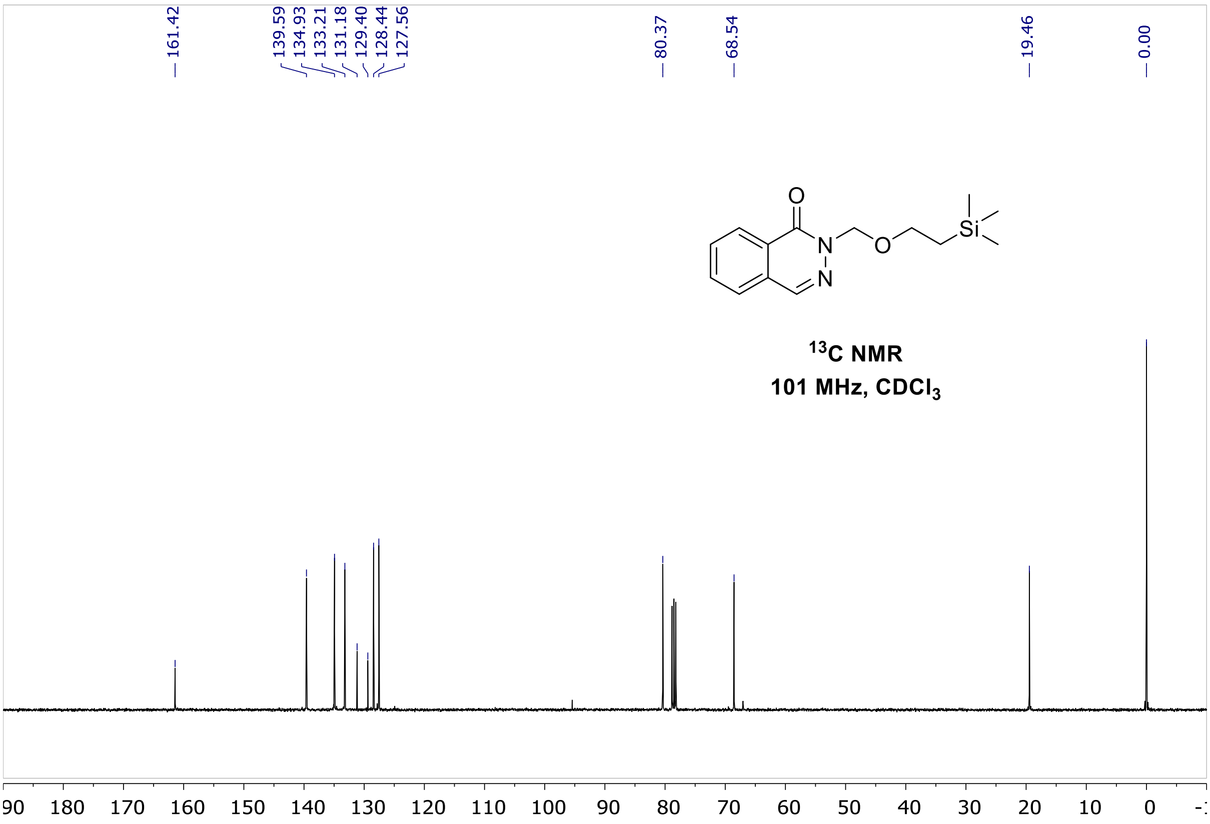

2-(4,4,5,5-Tetramethyl-1,3,2-dioxaborolan-2-yl)benzoic acid

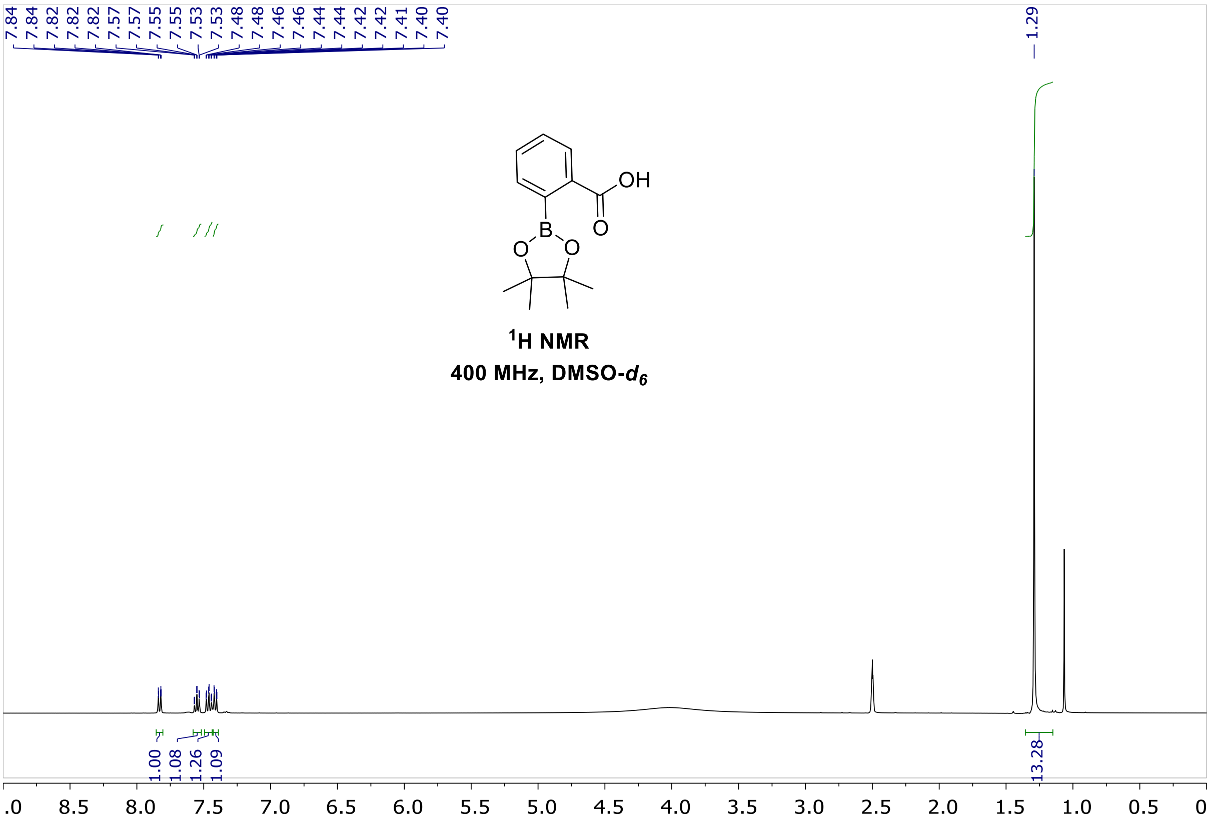

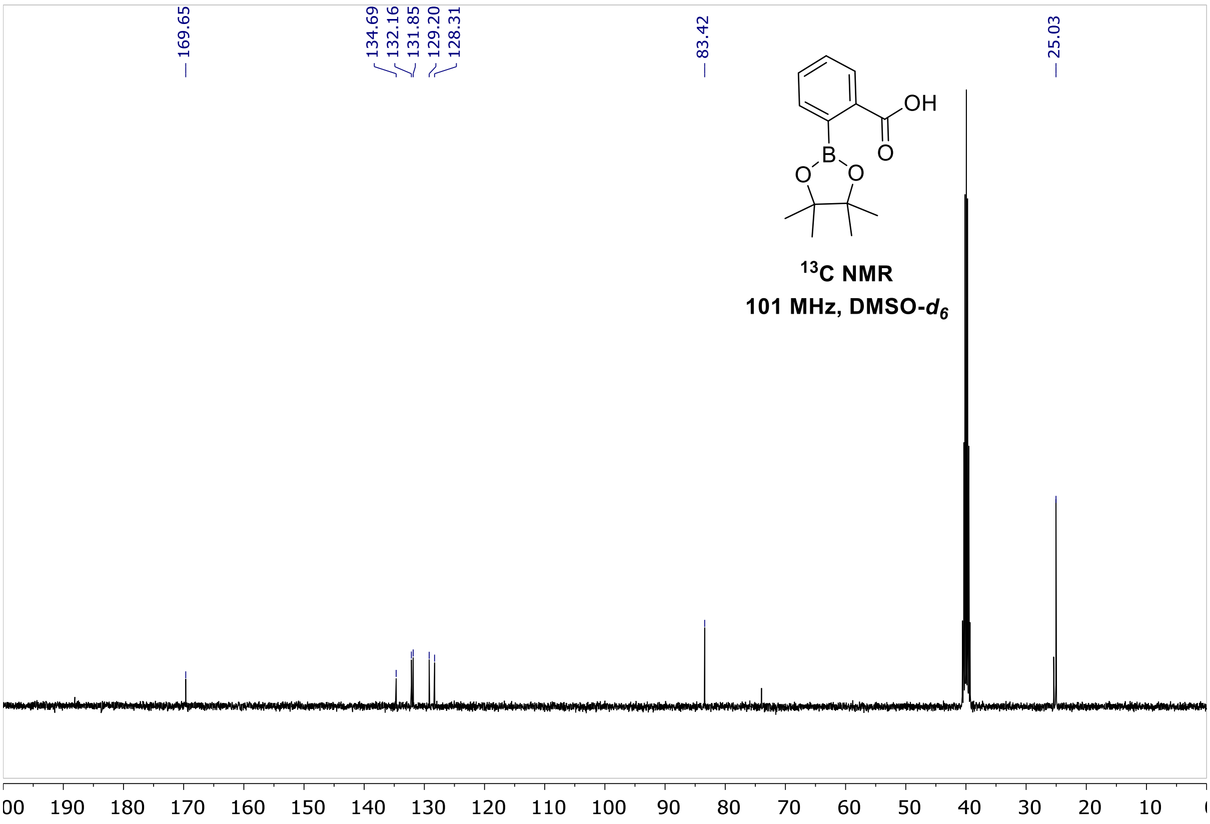

(4-(Cyclopropanecarbonyl)piperazin-1-yl)(2-(4,4,5,5-tetramethyl-1,3,2-dioxaborolan-2-yl)phenyl)methanone

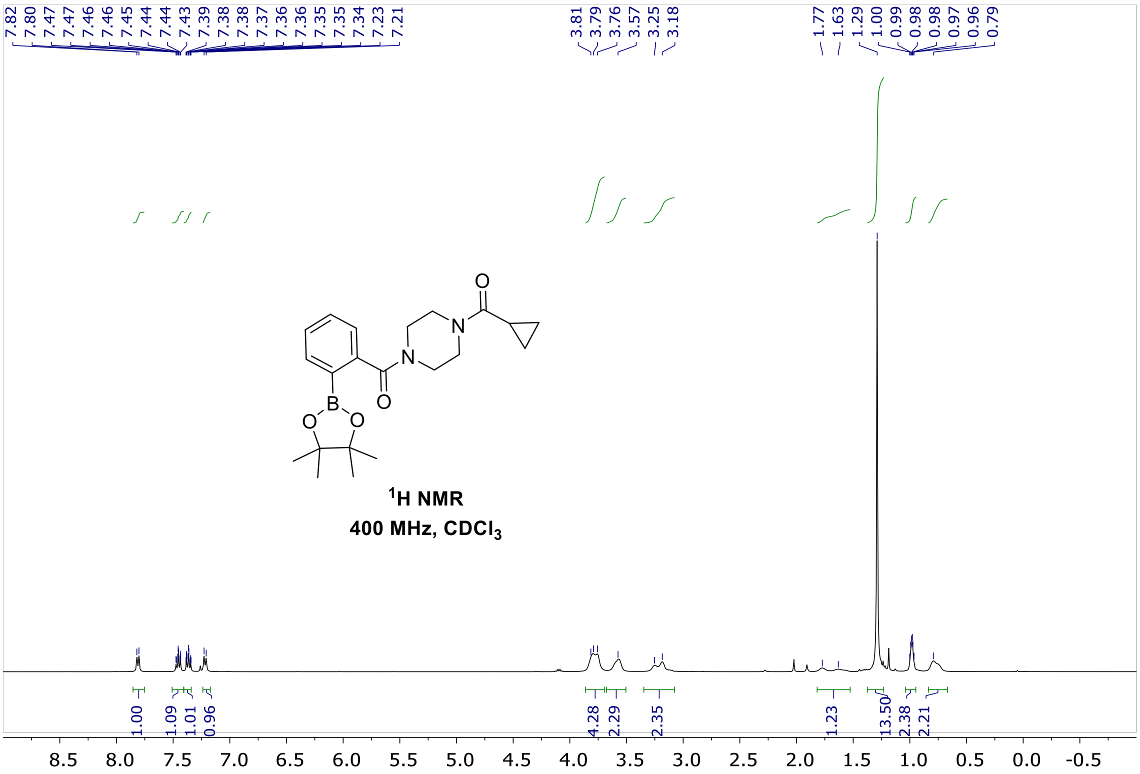

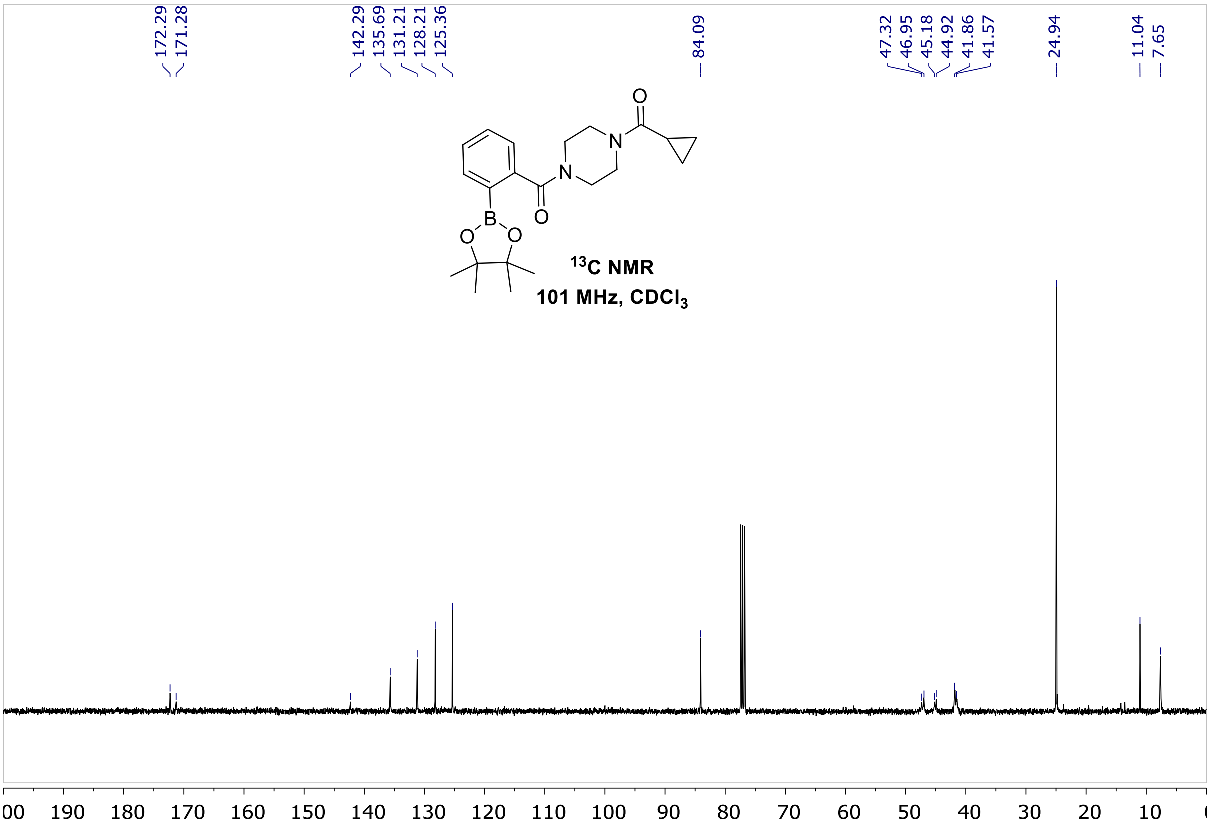

(4-(Cyclopropanecarbonyl)piperazin-1-yl)(2-fluorophenyl)methanone

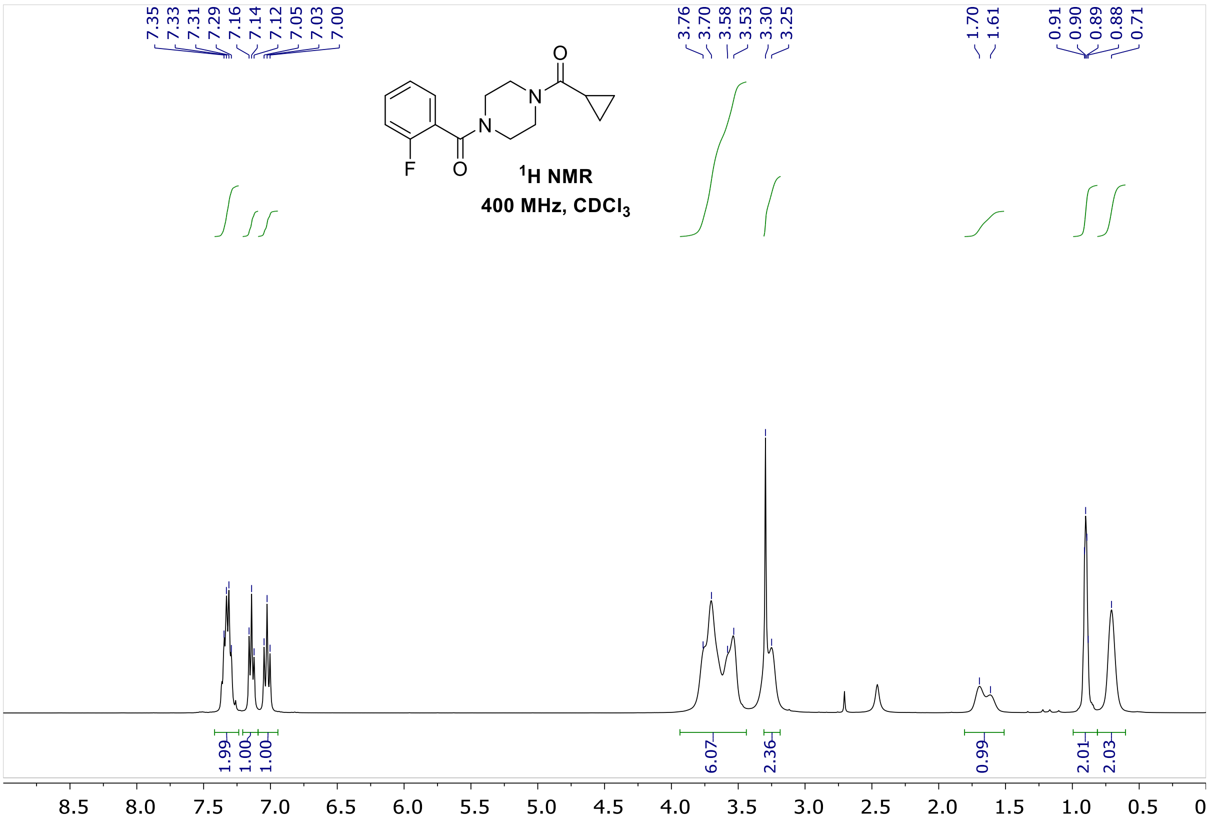

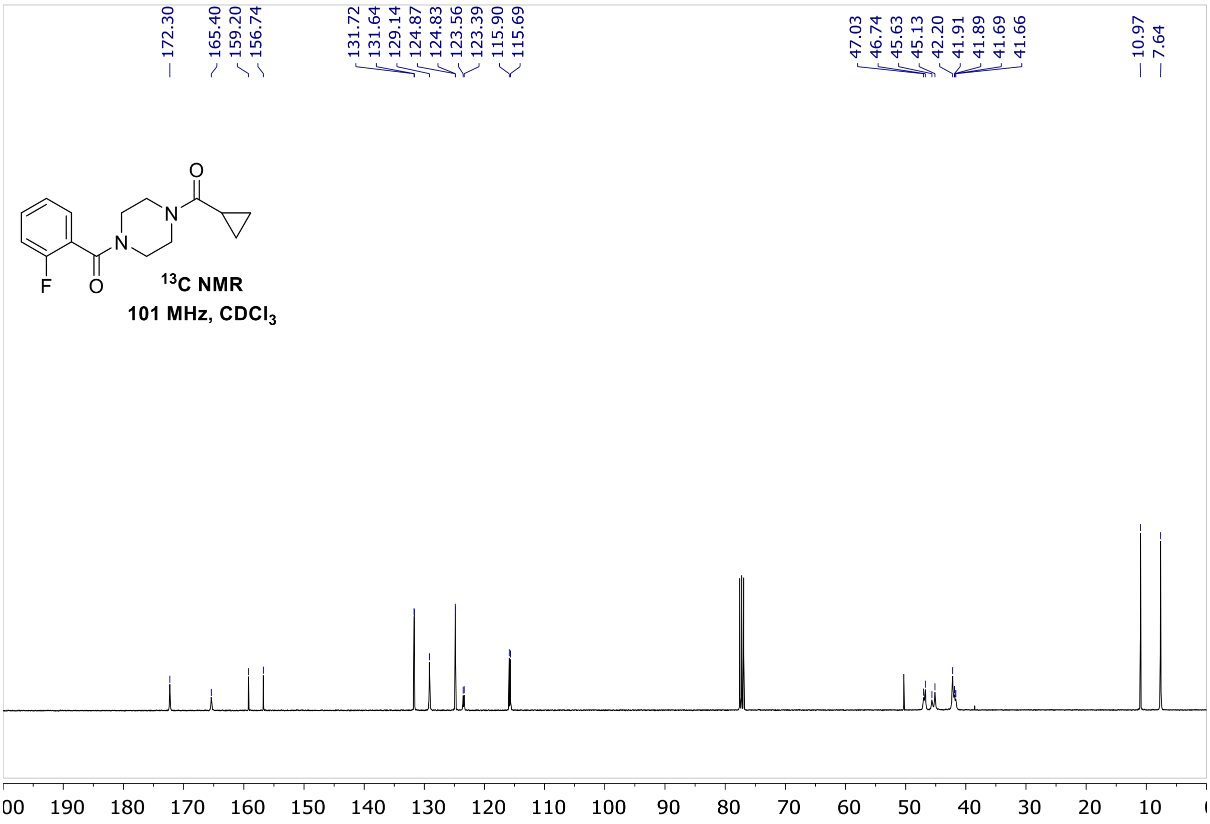

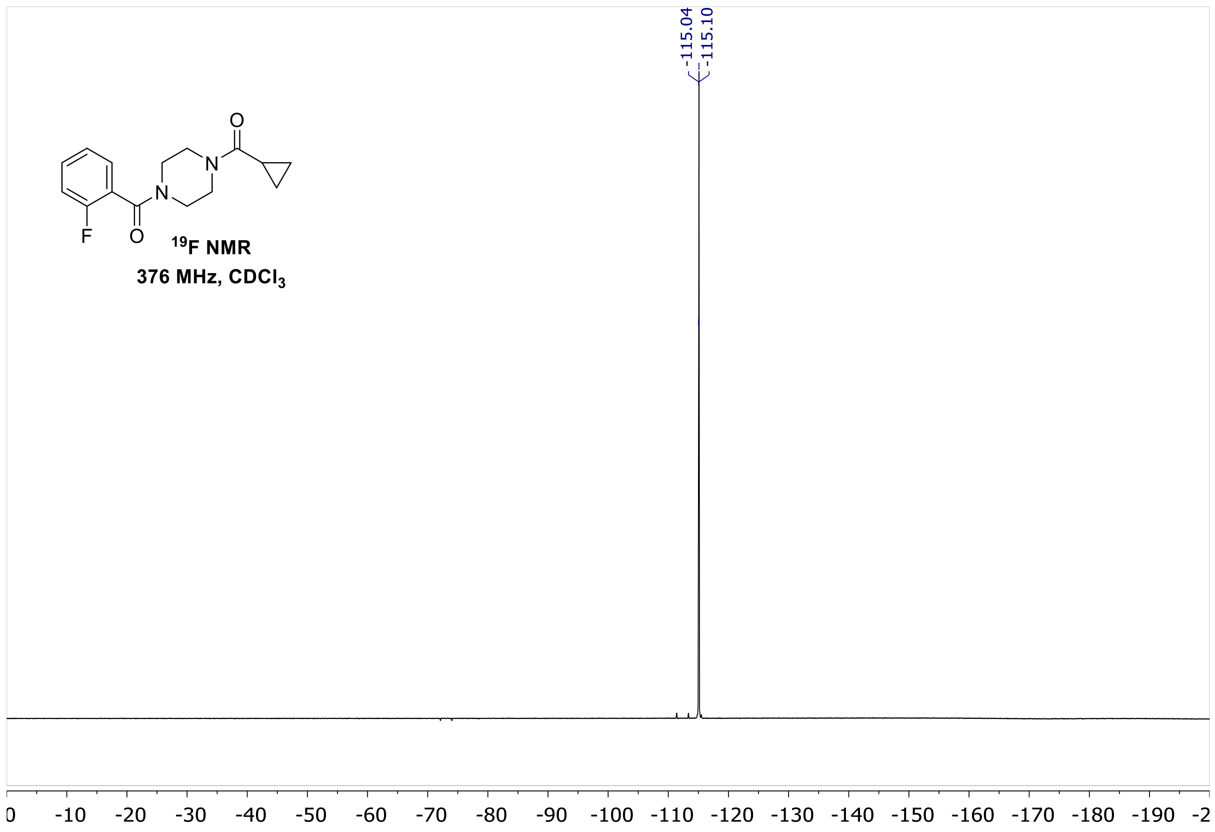

-115.04
-115.10
F
O
O
N
N
19F NMR
376 MHz, CDCl3
0 -10 -20 -30 -40 -50 -60 -70 -80 -90 -100 -110 -120 -130 -140 -150 -160 -170 -180 -190 -2

3-((4-oxo-3,4-dihydrophthalazin-1-yl)methyl)benzoic acid

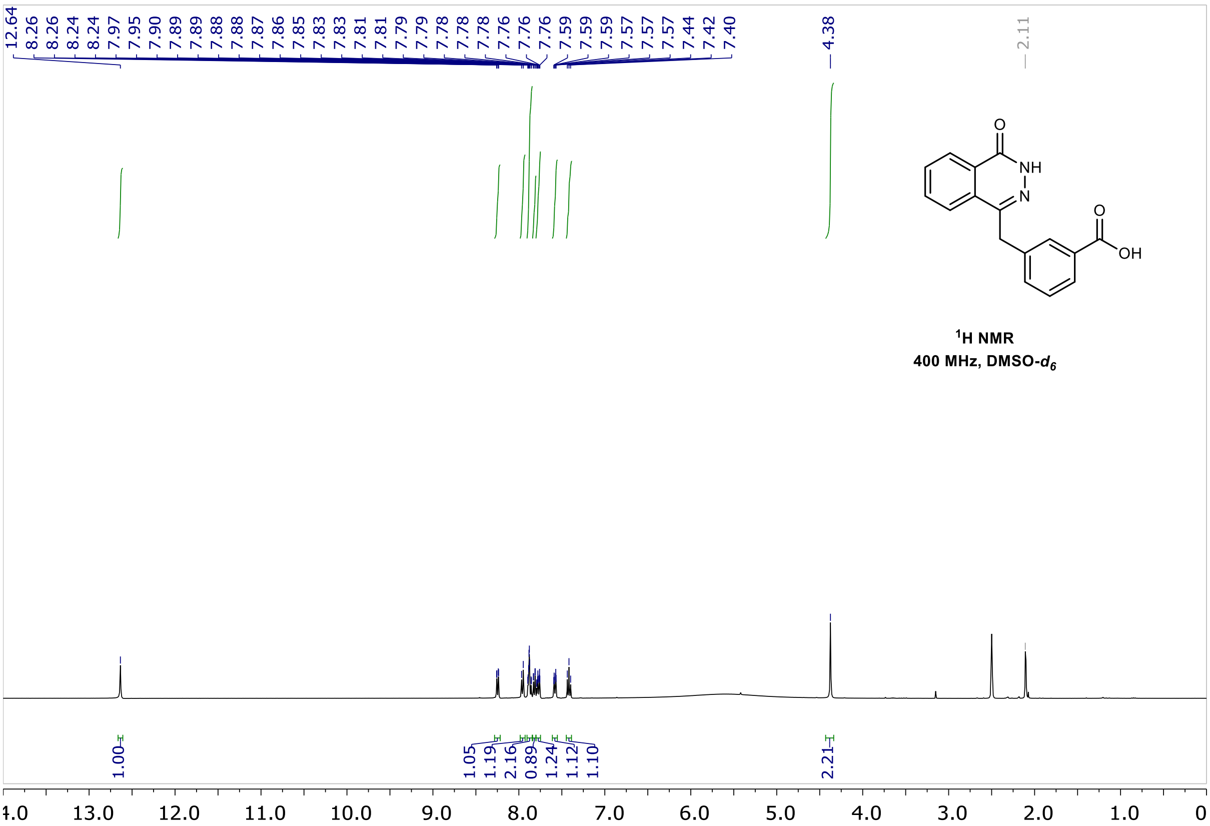

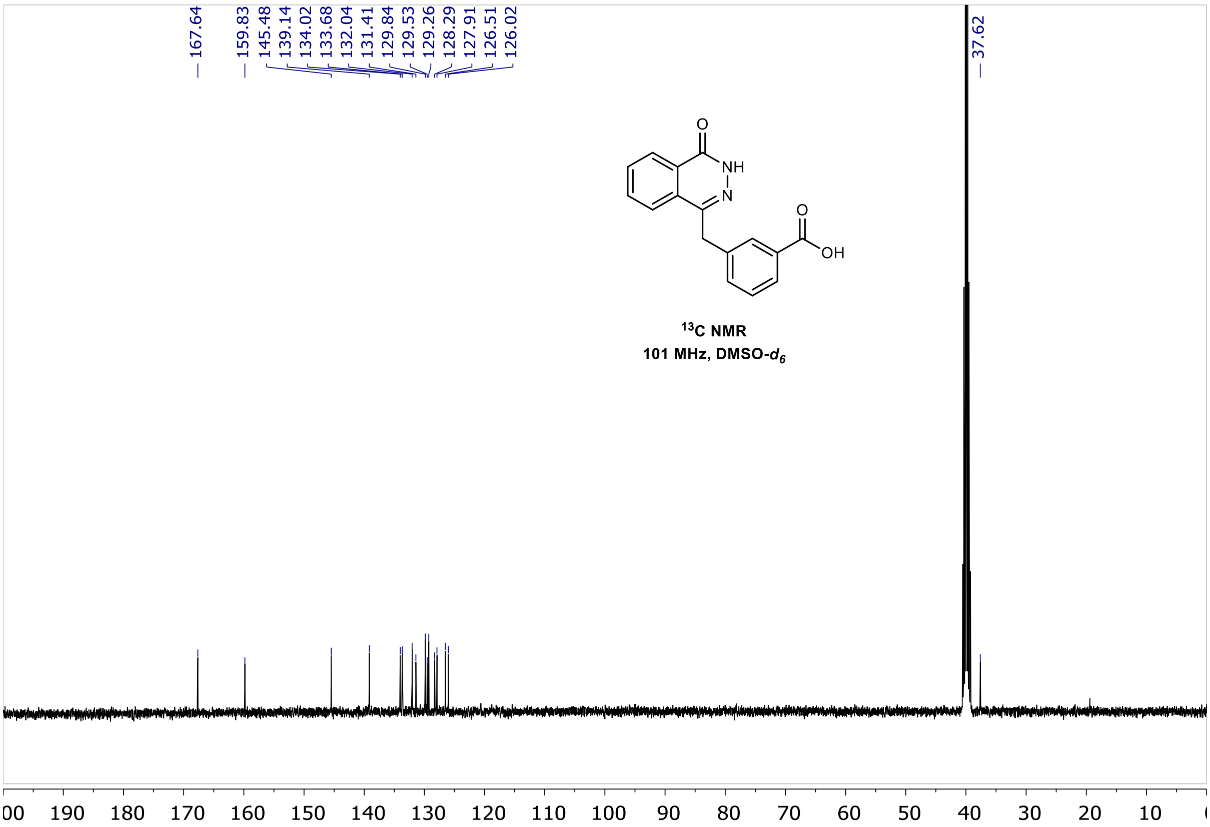

4-(3-(4-(cyclopropanecabonyl)piperazine-1-carbnonyl)benzyl)phthalazin-1(*2H*)-one

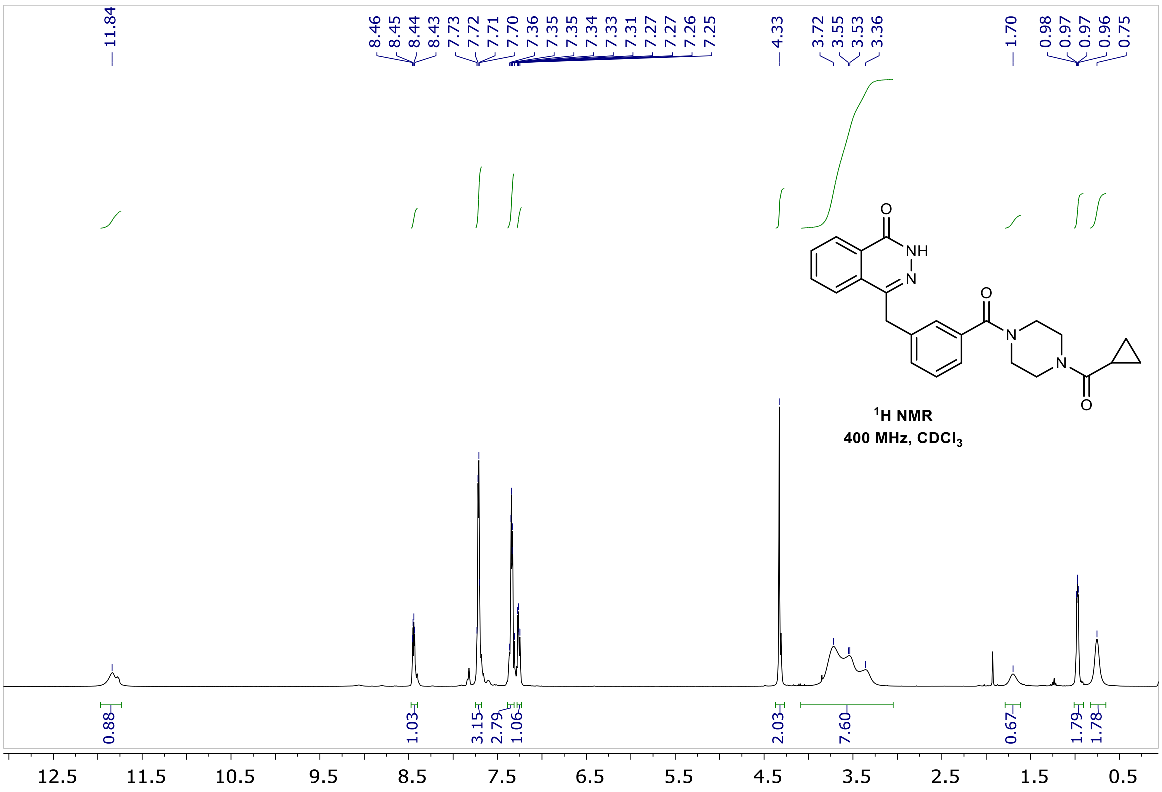

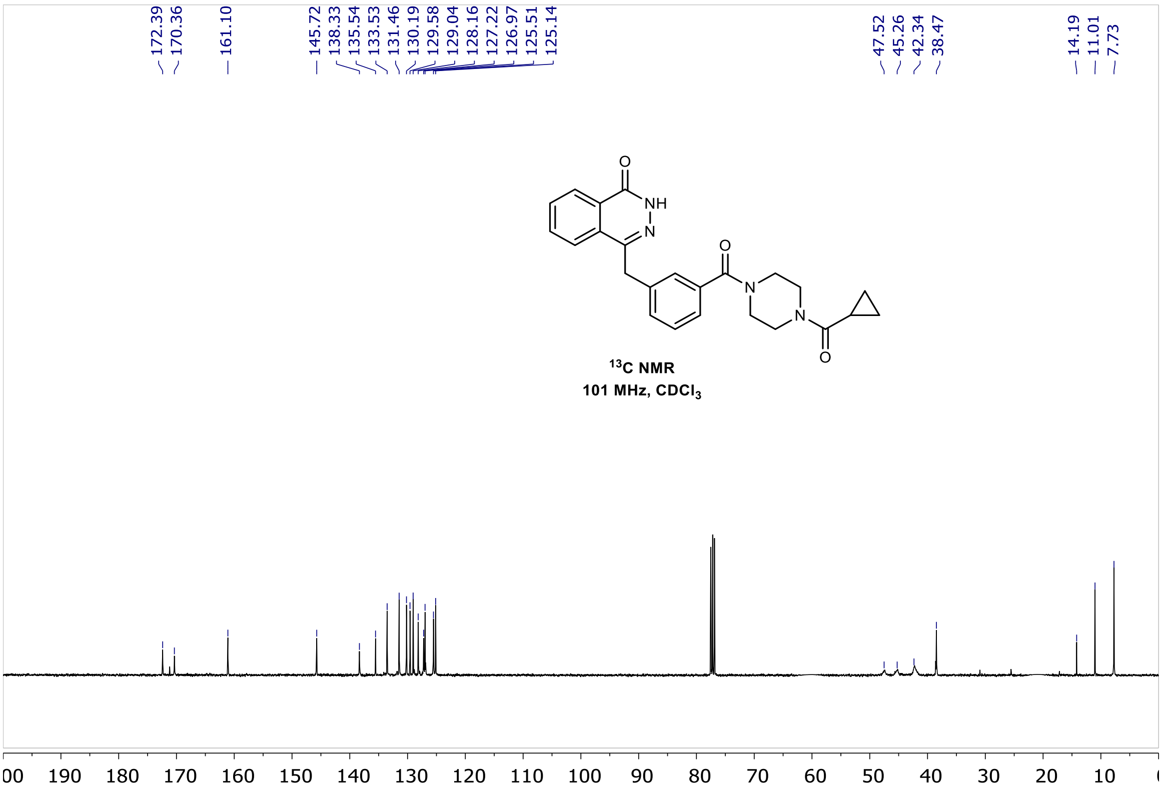

3. Supplemental Material: Biology

1. Cells

PSN-1, MiaPaCa-2, and Capan-1 human pancreatic duct adenocarcinoma cells were originally purchased from ATCC. Cells were maintained in Dulbecco's Modified Eagle Medium (DMEM), supplemented with 10% foetal bovine serum (FBS), 2 mM L-glutamine, 100 units/mL penicillin, and 0.1 mg/mL streptomycin. Cells were grown in a 37°C environment containing 5% CO_2 and were harvested and passaged as required using Trypsin-EDTA solution. Cells were authenticated by the provider and the cumulative length of culture was less than 6 months following retrieval from liquid nitrogen storage. Cells were regularly tested to confirm the absence of mycoplasma contamination. Capan-1 cells used in this study were subsequently found by STR profiling not to match with the ATCC-held profile. However, low PARP enzyme expression was confirmed by Western blot and immunohistochemistry.

2. Western Blot

Western blot probing for PARP-1 was performed after cells were exposed to external beam radiation (0 or 10 Gy; ^{137}Cs source, using a using an IBL-637 ^{137}Cs irradiator, Cisbio International; 1 Gy/min) with 2 or 24 hours recovery.

Total protein preparations were produced at 4°C on approximately 1×10^7 cells using RIPA lysis buffer (50 mM Tris, pH 8, 1% NP40, 0.5% sodium deoxycholate, 0.1% sodium dodecyl sulphate, 150 mM sodium chloride, cOmplete™ protease inhibitor cocktail [Sigma-Aldrich]). The cell lysates were isolated by centrifugation after lysis through a 21G hypodermic syringe and 30 second sonication. Thirty microgram lysate samples were run on a 4-12% Bis-Tris MES gel (Novex), transferred to a PVDF membrane and exposed to a 1:500 dilution of anti-PARP-1 antibody (Proteintech 13371-ap-1, lot 00045356) or anti-PGP-1 (abcam 170904, 1:2000), followed by a 1:3000 dilution of the secondary goat anti-rabbit-HRP (Bio-Rad). The membrane was exposed to autoradiography film after development using an ECL western blot substrate solution (Pierce Thermo Scientific 32209). β-actin was used as the loading control.

3. Immunocytochemistry

Cells were plated onto 8-chamber slides (Falcon CultureSlides) at 7×10^4 cells per chamber in 0.75 mL culture media and incubated overnight in a 37°C CO_2 incubator until approximately 80% confluent. The slides were briefly in phosphate-buffered saline (PBS) pH7.4, and the cells fixed in 4% formaldehyde/PBS for 10 min. The slides were washed in PBS for 3 x 5 minutes, and cells permeabilized

in 0.5% Triton X100 (Sigma) for 10 min. The cells were washed as before and non-specific binding was blocked by incubation of the slides in 2% BSA/PBS for 1 hour. To each appropriate section approximately 100µl primary antibody diluted 1:100 and 1:250 in 2% BSA/PBS, or just 2% BSA/PBS, was applied, and incubated in a humid chamber for 2 hours. The primary antibodies used was anti-PARP-1 rabbit polyclonal (ProteinTech 13371-ap-1). The slides were washed in PBS for 3 x 5 minutes, and the secondary goat-anti-rabbit IgG-594 antibody (Life Technologies Alexa Fluor, 1:500 dilution in 2% BSA/PBS), was applied and incubated for 1 hour. The slides were then washed in PBS for 3 x 5 minutes, excess fluid removed with a tissue, and a drop of Vectashield + DAPI (Vector Laboratories) was applied to each section. Finally a coverslip was gently lowered onto each slide, and Covergrip (Biotium) used to seal the coverslips. Prepared slides were stored at 4°C in the dark. The slides were analyzed using a Leica SP8 confocal fluorescent microscope.

4. Cell uptake experiments

Cell uptake of ^{18}F-olaparib in PSN-1, MiaPaCa-2 and Capan-1 cells was determined as previously described (*8-10*). Aliquots of cells (1.5×10^5 cells/well) were seeded in 24-well plates in warm cell culture medium (500 µL) and the cells were allowed to adhere overnight. Cells were irradiated (10 Gy; dose rate 0.8 Gy/min) or sham-irradiated and then returned to an incubator (37°C, 5% CO_2) for 2-48 h. The cell culture medium was then removed and cells were washed once with fresh cell culture medium (500 µL). In 500 µL of cell culture medium (not supplemented with FBS, L-glutamine, or penicillin/streptomycin), ^{18}F-olaparib (50 kBq) was added to each well and the cells were then incubated at 37°C. In the blocking groups, non-radioactive olaparib, talazoparib, or rucaparib were also added in increasing concentrations (10 pM - 10 µM. After 30 or 60 minutes, the cell culture medium was removed and combined with two washes (500 µL) of cell culture medium. The remaining monolayer of cells was then lysed with 0.1 M sodium hydroxide for 20 minutes at room temperature. The amount of radioactivity contained within the cell culture medium and the cell lysate fractions was measured using a gamma counter. Protein levels from parallel plates were quantified using a Pierce BCA protein assay kit (Thermo Scientific) according to the manufacturer's recommendations and bovine serum albumin was used as the protein standard. Cell uptake levels of ^{18}F-olaparib were normalized to percent of the total added radioactivity per milligram protein. These experiments were performed in triplicate on at least three separate occasions. IC50 values were calculated using GraphPad Prism software (GraphPad Software, San Diego, CA, USA). Data are presented as mean ± SEM.

(Radiation dose dependency)

Aliquots of cells (7.5×10^4 cells/well) were seeded in 24-well plates in warm cell culture medium (500 µL). After 4 h, cells were irradiated (0, 2, 4, 6, 8, or 10 Gy; dose rate 0.8 Gy/min) and then returned to an incubator (37°C, 5% CO_2). After 24 or 48 h, the cell culture medium was then removed and cells were washed once with fresh cell culture medium (500 µL). In 500 µL of cell culture medium (not supplemented with FBS, L-glutamine, or penicillin/streptomycin), ^{18}F-olaparib (50 kBq) was added to each well and the cells were then incubated at 37°C. In the blocking groups, non-radioactive olaparib was also added to each well to achieve a concentration of 10 µM. After 30 minutes, the cell culture medium was removed and combined with two washes (500 µL) of cell culture medium. The remaining monolayer of cells was then lysed with 0.1 M sodium hydroxide for 20 minutes at room temperature. The amount of radioactivity contained within the cell culture medium and the cell lysate fractions was measured using a gamma counter. Protein levels from parallel plates were quantified using a Pierce BCA protein assay kit (Thermo Scientific) according to the manufacturer's recommendations and bovine serum albumin was used as the protein standard. Radiotracer cell uptake levels were normalized to percent of the total added radioactivity per milligram protein.

5. *In vivo* tumor models

All animal procedures were performed in accordance with the UK Animals (Scientific Procedures) Act 1986 and with local ethical committee approval. Animals were housed in IVC cages in sex-matched groups up to 5 per cage, in an artificial day-night cycle facility, with ad libitum access to food and water.

(PSN-1 and Capan-1)

Cells were harvested using trypsin, washed twice using PBS, and reconstituted in DMEM. PSN-1 or Capan-1 xenograft tumors were established in the right hind flank of female NOD/SCID mice (Charles River, UK) by subcutaneous injection of 1×10^6 cells in PBS:matrigel (150 µL). Tumor volumes (V) were calculated after calliper measurement using the following equation: $V = (a^2 \times b)/2$, where a is the width of the tumor and b the length (small and large diameters, respectively). The individual relative tumor volume (RTV) was defined as Vt/V_0, where Vt is the volume at a given time and V_0 at the start of treatment. Animals were entered in in vivo studies when their tumor was at least 200 mm^3.

(CaNT)

The murine adenocarcinoma NT (CaNT) was implanted subcutaneously onto the right thigh of 6-7 week-old female CBA/Carl mice. Fifty µL of a crude cell suspension, prepared by mechanical

dissociation of an excized tumor from a donor animal, was injected. Tumors were selected for imaging when the geometric mean diameter reached 6-8 mm (volumes calculated as above), approximately 3 weeks after implantation (*11*).

(Irradiation of tumors)

Irradiation of subcutaneous tumor xenografts was performed using a Gulmay 320 kV system (2.0 Gy/min). A dose of 10 Gy was delivered to the tumor using 300 kV X-rays (Gulmay 320kV irradiator; 2 Gy/min). The radiation set-up allowed irradiation of the right hind quarter, including the tumor and right leg, only. Control mice were anesthetized and sham-irradiated for the same length of time. Irradiation was performed 2 hours prior to administration of ^{18}Folaparib (see figure 3a, here repeated for clarity).

6. PET/CT Imaging

(^{18}F-olaparib PET imaging)

Two hours prior to PET/CT imaging, animals were exposed to X-irradiation of the tumor (10 Gy or sham) (see schedule below). Radiation was delivered using a Gulmay 320 kV X-irradiator; 2.0 Gy/min. Anesthesia was maintained at 2.5 % isoflurane throughout the duration of the irradiation, and animals were allowed to recover. To study the relationship of PARP-1 expression, ^{18}F-olaparib uptake and hypoxia, the same animals were administered EF5 (0.6 µg in 200 µL 0.9% saline) intraperitoneally, one hour prior to PET/CT imaging. Some animals were administered an excess of cold, unlabelled olaparib (0.5 mg in 250 ml 10%DMSO (v/v) in PBS), intravenously, 30 minutes prior to administration of ^{18}F-olaparib. Naïve CBA/Carl mice, not bearing tumors, were not irradiated, nor was EF5 administered.

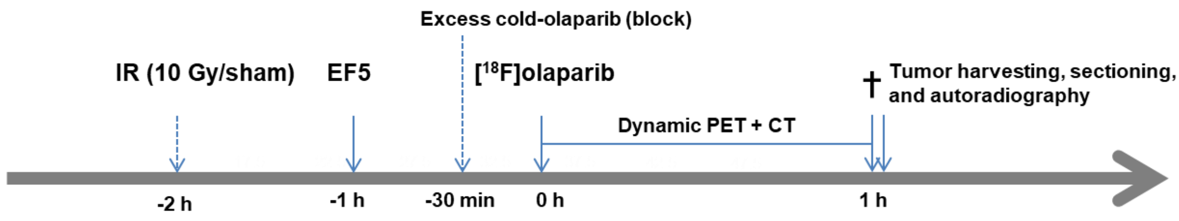

Dynamic PET/CT images were acquired using a VECTor^{4}CT integrated PET/CT system. Mice were anesthetized by 4% isoflurane gas (0.5 L/min O_2) and placed on a custom-built imaging cradle in a prone position. Animals were intravenously injected with ^{18}F-olaparib or (3-5 MBq, 8-11 GBq/µmol, through a tail vein catheter, and dynamic imaging was performed over 45-60 min after radiolabeled compound administration. PET acquisition (150 s per frame, using list mode acquisition) using an ultra-high resolution rat/mouse 1.8 mm collimator, followed by a cone-beam CT scan (55 kV, 0.19 mA) for

anatomical reference and attenuation correction. Anesthesia was maintained at 2.5 % isoflurane throughout the duration of the image acquisition. PET images were reconstructed using U-SPECT-Rec3.22 software (MILabs, Utrecht, The Netherlands), applying a pixel-based algorithm with 8 subsets, 6 iterations and 0.8 mm voxel size for ^{18}F (energy window settings 477.9-584.1 keV).

(image analysis)

Reconstructed images were viewed and analyzed using PMOD v.3.37 (PMOD Technologies, Zurich, Switzerland). The radioactivity in each volume of interest was calculated as percent injected dose per cubic cm (%ID/mL).

7. *Ex vivo* biodistribution

After PET/CT image acquisition, animals were euthanized by cervical dislocation and selected organs, tissues and blood were removed. The amount of radioactivity in each organ was measured using a 2480 WIZARD^2 gamma counter (PerkinElmer). Counts per minute were converted into MBq using a calibration curve generated from known standards. Values were decay-corrected to the time of injection, and the percentage of the injected dose per gram (%ID/g) of each tissue was calculated.

8. *Ex vivo* analysis

(Autoradiography)

After imaging and automated gammacounting, selected tissues from mice were flash-frozen with dry ice. If required, samples were stored at -80°C overnight. Frozen tissue was sectioned (8 µm) using an OTF5000 cryotome (Bright Instruments Ltd). Tissue sections were thaw-mounted onto Superfrost PLUS glass microscope slides (Menzel-Glaser, Thermo Scientific) and allowed to dry at room temperature. The slides were then exposed to a storage phosphor screen (PerkinElmer, Super Resolution, 12.5 x 25.2 cm) in a standard X-ray cassette for 15 h at 4°C or -20°C. The phosphor screen was then imaged using a Cyclone® Plus Storage Phosphor System (PerkinElmer) and images were analyzed with OptiQuant 5.0 (PerkinElmer) and ImageJ (NIH).

(Immunohistochemistry)

PARP-1 staining:

PSN-1 xenografts harvested from mice were flash frozen and 8 μm sections were prepared using a cryostat. Sections were stored at -80°C until use. Slides were allowed to reach room temperature (10 minutes), then washed briefly in phosphate-buffered saline (PBS) pH7.4. The slides were fixed in 4% formaldehyde/PBS for 10 min, then washed three times in PBS for 5 min. Sections were permeabilized

in 0.5% Triton X100 (Sigma) for 10 min, washed, and non-specific binding was blocked by incubation of the slides in 2% BSA/PBS for 1 h. Slides were briefly allowed to dry and each section was isolated using a PAP pen (Sigma). To each appropriate section approximately 100 µl primary anti-PARP-1 polyclonal antibody (ProteinTech 13371-AP-1) diluted 1:250 in 2% BSA/PBS, or just 2% BSA/PBS, was applied, and incubated in a humid chamber overnight at 4°C. The slides were washed three times in PBS for 5 min, and the secondary goat-anti-rabbit IgG-488 antibody (Life Technologies Alexa Fluor, 1:500 dilution in 2% BSA/PBS), was applied and incubated for 1 h. The slides were then washed in PBS, excess fluid removed, mounted using Vectashield containing DAPI (Vector Laboratories). Slides were stored at 4°C in the dark. Images were acquired using a Leica SP8 confocal fluorescent microscope.

EF5 staining:

Tumor hypoxia was confirmed by immunohistological staining for EF5 (2-(2-nitro-1H-imidazol-1-yl)-N-(2,2,3,3,3-pentafluoropropyl)-acetamide). For EF5 studies, mice were administered with 10 mM EF5 in 0.9% saline i.v. 2 h prior to tumor excision (EF5 was obtained from Dr. Cameron Koch, University of Pennsylvania (*12*)). To determine the correlation between ^{18}F-olaparib uptake and hypoxia, tumor slices were analyzed by both autoradiography and EF5 IHC (*13*) (*11*) (*14*). Object-based overlap between both modalities was determined by first co-registering autoradiography and fluorescence microscopy images using a rigid transformation. Then, Manders' overlap coefficients (M1) were calculated using the JACoP plug-in for Image J (methods of Manders for spatial intensity correlation analysis with Costes method for automatic thresholding).

9. Statistical methods

All statistical analyses and nonlinear regression were performed using GraphPad Prism (GraphPad Software, San Diego, CA, USA). Data were tested for normality and analyzed either by the unpaired, two-tailed Student's t-test where appropriate, or 1-way analysis of variance (ANOVA) for multiple comparisons, with Dunnet's post-tests to calculate significance of differences between groups. All data were obtained at least in triplicate and results reported as mean ± standard deviation, unless stated otherwise.

10. Supplemental tables: biodistribution data

Supplemental Table 4: *Ex vivo* biodistribution ^{18}F-olaparib in naïve CBA/Carl mice.

	%ID/g		
Blood	0.11	0.11	0.14
Heart	0.19	0.17	0.34
Lung	0.29	0.19	0.34
Liver	2.55	4.02	5.27
Spleen	2.09	1.91	3.68
Stomach	0.46	0.26	0.58
Large intestine	0.92	1.64	2.99
Small intestine	7.75	10.33	17.40
Pancreas	0.91	0.70	1.30
Kidney	0.59	0.60	1.01
Muscle	0.20	0.14	0.34
Skin	0.16	0.10	0.27
Fat	0.05	0.03	0.26

Supplemental Table 5: *Ex vivo* biodistribution ^{18}F-olaparib in PSN-1 xenograft bearing mice.

	**^{18}F-olaparib**						**^{18}F-olaparib + IR (10 Gy)**			**^{18}F-olaparib + block**			
Blood	0.41	0.73	0.57	0.24	0.21	0.19	0.63	0.83	1.17	2.12	2.29	2.51	1.06
Tumor	1.94	4.07	4.20	3.27	2.43	3.07	6.43	3.03	6.59	1.68	0.92	1.09	1.09
Heart	3.18	2.05	1.74	1.58	0.85	1.07	3.14	2.49	2.88	1.98	1.59	1.51	0.78
Lung	4.68	3.51	3.18	2.15	1.45	1.69	3.94	4.47	4.47	1.61	1.40	1.32	0.74
Liver	26.2	34.6	27.0	21.0	13.5	18.0	25.1	33.8	45.7	29.1	32.9	22.4	23.1
Spleen	17.5	13.7	11.3	14.0	7.36	8.01	45.0	20.5	14.3	1.62	1.46	1.24	0.69
Stomach	2.59	3.53	1.81	1.80	0.55	0.60	4.15	0.92	0.87	1.44	0.79	0.46	0.21
Large intestine	12.6	30.5	80.8	27.0	28.9	6.61	27.5	28.9	16.2	20.1	13.3	61.4	12.7
Small intestine	54.5	58.1	30.6	31.9	18.6	26.6	65.1	113	95.2	89.9	77.2	96.4	132
Pancreas	8.49	9.38	7.25	5.52	2.78	4.24	13.6	10.3	11.4	1.92	2.29	1.91	1.79
Kidney	10.5	7.95	6.02	7.04	3.41	3.66	10.0	9.25	10.6	13.1	5.09	3.98	2.44
Muscle	1.60	1.40	1.45	1.12	0.70	0.73	3.71	2.01	1.76	1.58	1.08	1.18	0.65
Bone	3.38	4.54	2.71	1.88	1.26	1.50	9.12	4.13	3.81	1.06	0.78	0.80	0.46
Skin	1.50	1.75	2.06	1.25	0.87	1.14	2.10	2.38	2.66	1.65	1.09	1.10	0.66
Fat	0.97	0.82	0.68	0.45	0.44	0.37	0.66	1.41	0.82	0.53	0.62	0.35	0.19

Supplemental Table 6: *Ex vivo* biodistribution ^{18}F-olaparib in Capan-1 xenograft bearing mice.

	**^{18}F-olaparib**				**^{18}F-olaparib + IR (10 Gy)**			**^{18}F-olaparib + block**		
Blood	0.30	0.23	0.31	0.72	0.86	0.43	1.16	0.25	0.27	0.56
Tumor	2.48	3.21	2.94	1.73	1.85	1.70	2.48	1.27	1.04	0.69
Heart	1.75	0.87	1.81	2.24	0.92	0.48	0.80	0.53	1.62	1.83
Lung	2.60	1.49	2.66	3.97	0.83	0.60	0.85	0.90	2.04	1.86
Liver	11.78	5.79	10.32	22.63	9.16	5.93	6.67	3.62	6.88	9.79
Spleen	9.84	6.13	8.40	15.10	0.77	0.73	0.82	2.80	7.93	6.43
Stomach	1.55	0.63	1.14	3.42	0.38	0.36	0.64	0.30	0.49	0.94
Large intestine	4.99	4.50	4.14	8.14	5.21	3.30	5.86	2.91	4.06	5.00
Small Intestine	12.27	17.29	11.58	13.70	15.58	9.80	19.97	0.45	11.70	46.04
Pancreas	9.32	8.12	7.87	13.16	1.89	1.59	1.84	4.56	10.82	10.22
Kidney	4.32	2.05	4.91	5.22	4.66	1.03	3.67	1.26	3.32	4.16
Muscle	0.65	0.45	0.63	1.44	0.63	0.29	0.48	0.28	0.65	0.88
Bone	1.46	0.98	1.26	3.08	0.31	0.16	0.19	0.47	1.27	2.17
Skin	0.02	0.55	0.77	1.54	0.67	0.23	0.33	0.14	0.46	1.16
Fat	0.46	0.19	0.27	0.69	0.24	0.13	0.19	0.22	0.77	0.36
Brain	0.82	0.03	0.03	0.10	0.06	0.03	0.03	0.03	0.03	0.04
Caecum	5.11	4.35	3.31	24.11	7.58	4.46	5.92	9.13	4.00	5.07
Gallbladder	81.02	24.53	34.02	446.94	68.68	31.81	36.90	105.86	35.11	88.82

Supplemental Table 7: *Ex vivo* biodistribution ^{18}F-olaparib in CaNT allograft bearing CBA/Carl mice.

	**^{18}F-olaparib**		**^{18}F-olaparib + IR (10 Gy)**		**^{18}F-olaparib + block**
Blood	1.78	8.00	9.79	3.12	4.00
Tumor	1.48	2.83	1.43	1.92	0.98
Heart	7.34	0.00	8.74	7.03	2.51
Lung	12.5	9.73	10.9	9.61	2.29
Liver	84.6	110	93.2	100	74.3
Spleen	43.3	47.0	69.0	58.3	2.41
Stomach	6.17	12.8	9.40	4.20	4.09
Large intestine	24.0	47.8	60.1	47.0	61.2
Small intestine	200	279	455	190	603
Pancreas	15.1	20.8	29.5	23.6	3.41
Kidney	23.9	21.0	24.5	19.0	8.45
Muscle	5.84	4.63	5.45	5.31	2.65
Bone	11.7	10.2	15.7	11.5	5.18
Skin	3.82	4.65	5.02	5.12	1.16
Fat	7.19	1.12	2.52	2.34	1.57
Brain	0.29	0.23	0.20	0.25	0.22
Caecum	32.5	56.2	42.9	44.6	101
Gallbladder	953	126	155	57.3	194

11. Supplemental Results

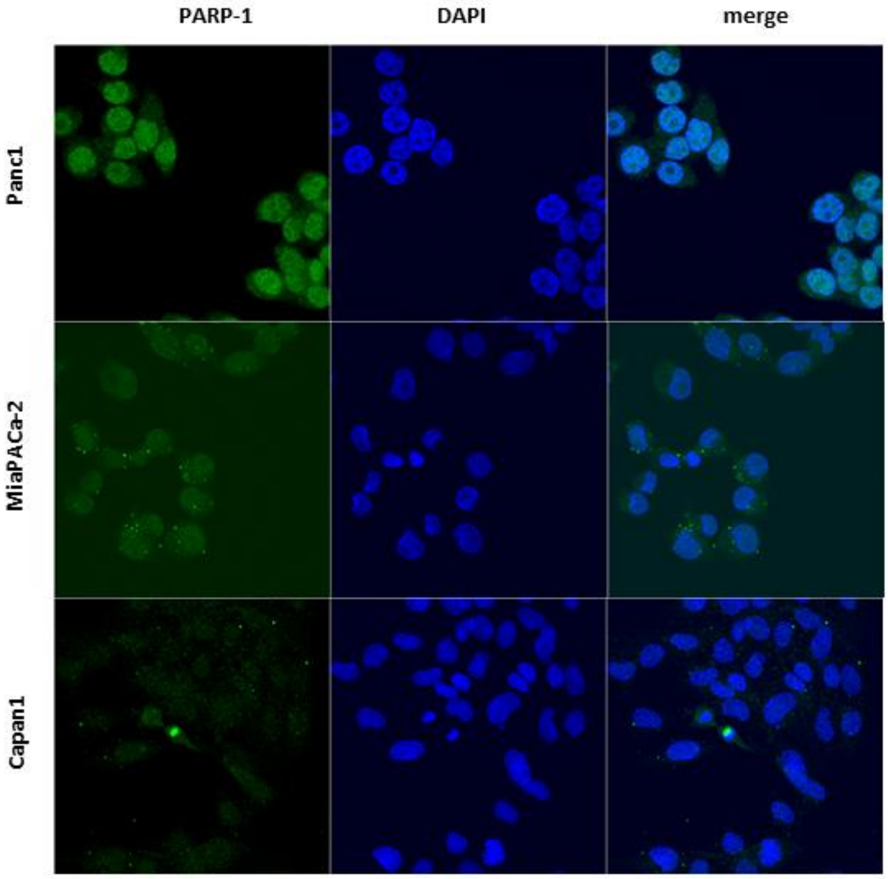

Supplemental Fig 8: *Immunocytochemistry of PARP-1 in a selection of PDAC cell lines.*

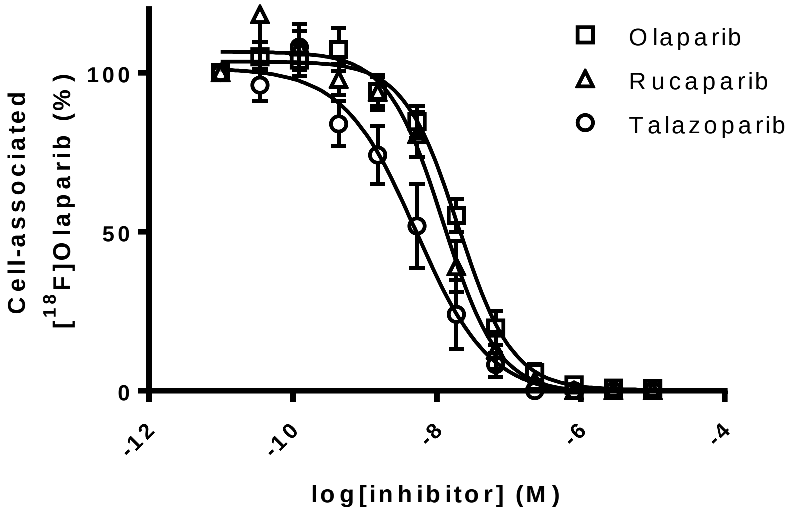

Supplemental Fig 9: *Displacement of* ^{18}F-olaparib *uptake in PSN-1 cells by a selection of several PARP inhibitors.*

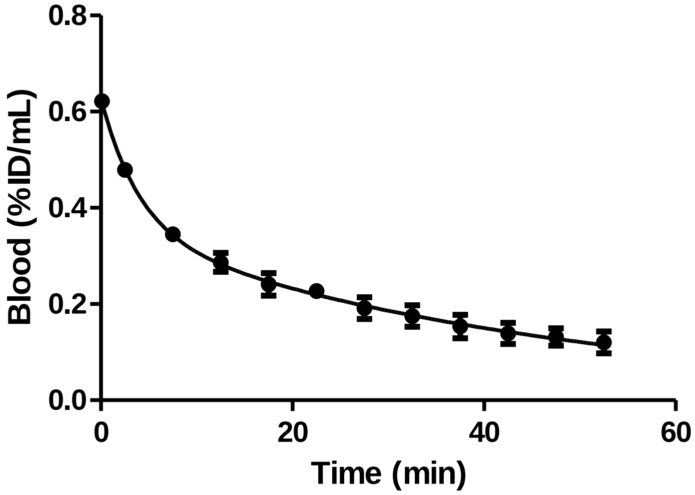

Supplemental Fig 10*: Concentration of* ^{18}F-olaparib *in blood of naïve CBA*/Carl *mice.*

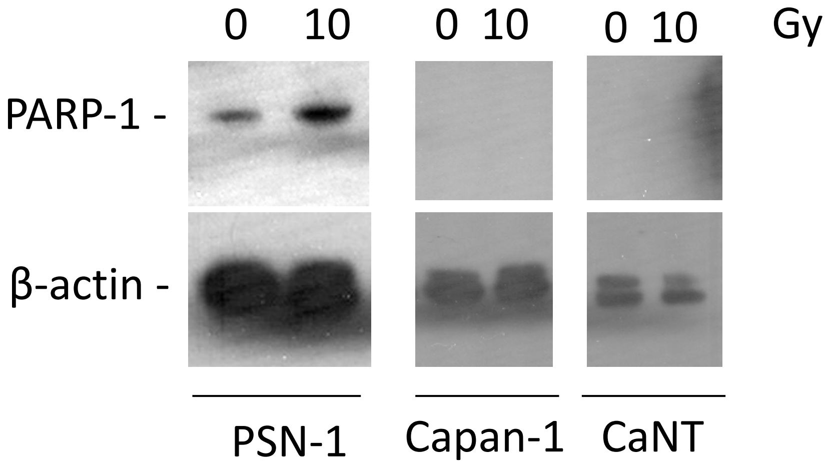

Supplemental Fig 11*:Western blot of xenograft tumor tissue from PSN-1, Capan-1, and CaNT tumors, after irradiation (10 Gy) or sham-irradiation (0 Gy). Xenografts from randomly selected animals were analyzed.*

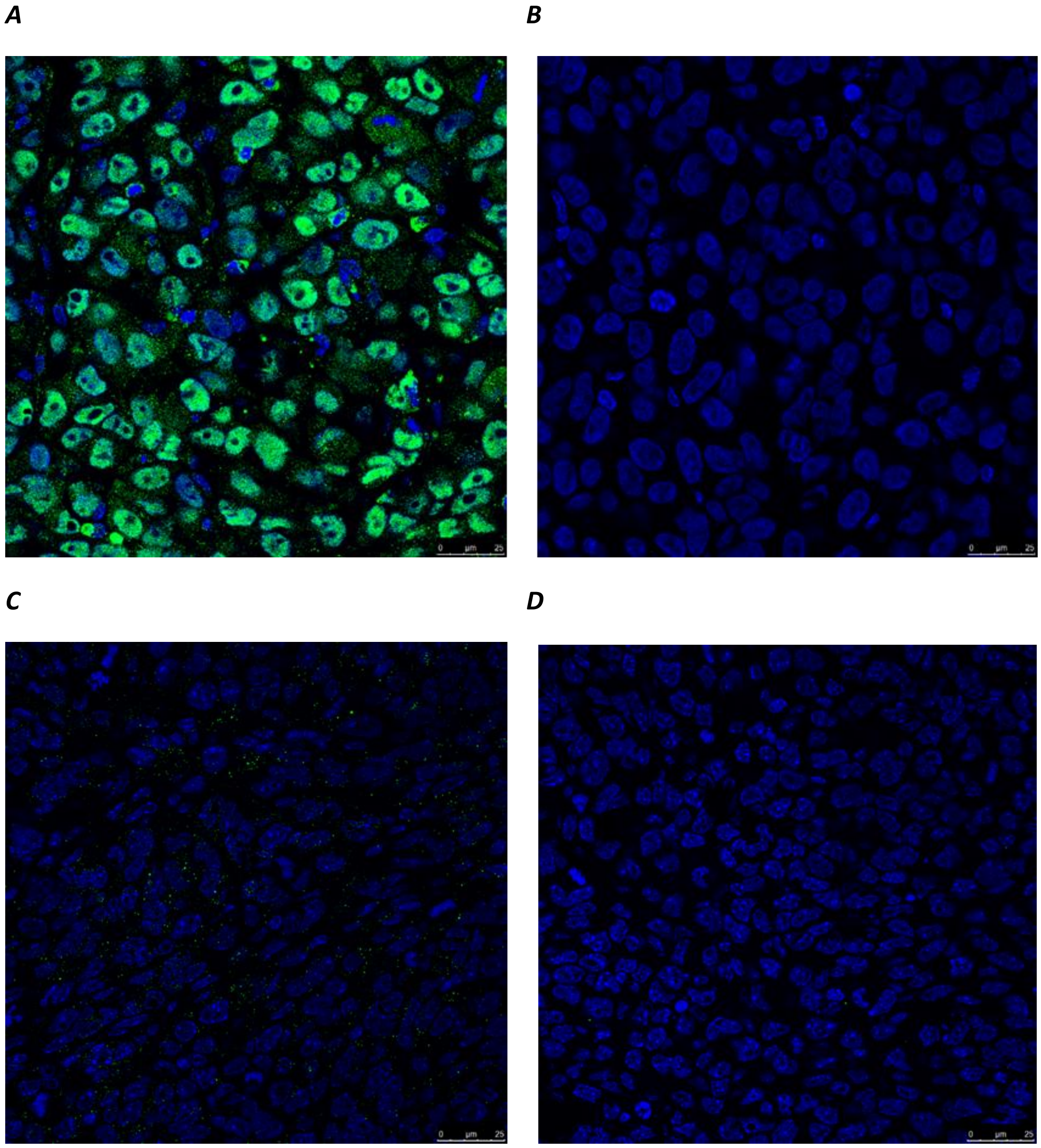

Supplemental Fig 12*: Immunohistochemistry staining for PARP-1 on xenograft tumor tissue from PSN-1 (A), CaNT tumors (C). Controls using only secondary antibody showed minimal non-specific staining (B, D).*

A

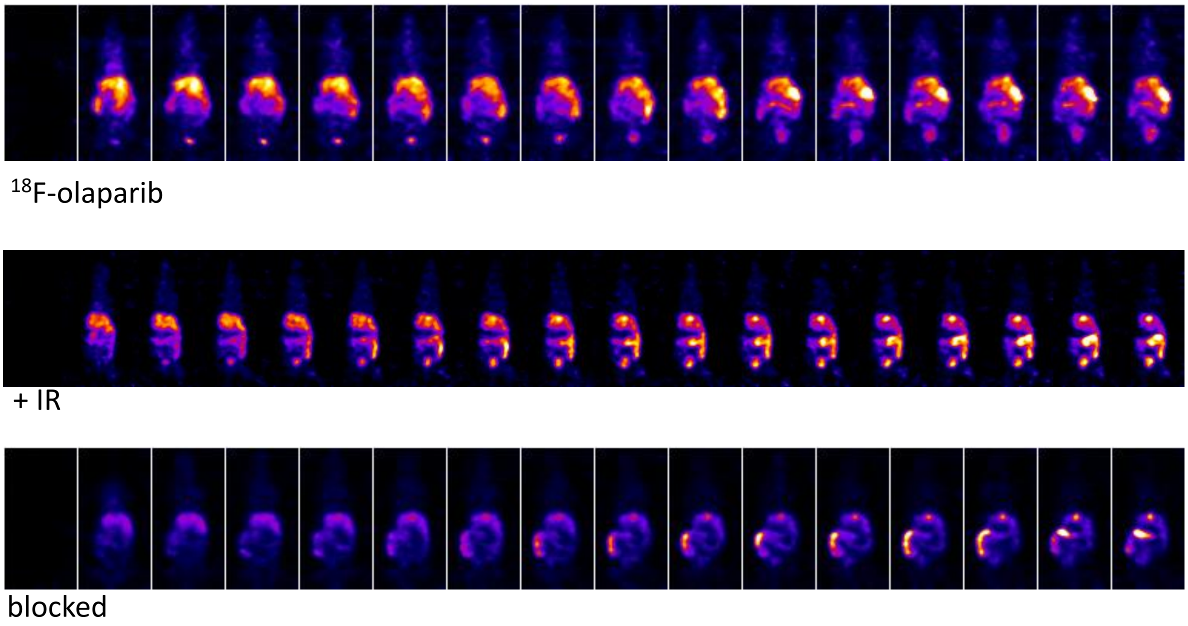
^{18}F-olaparib
+ IR
blocked

B

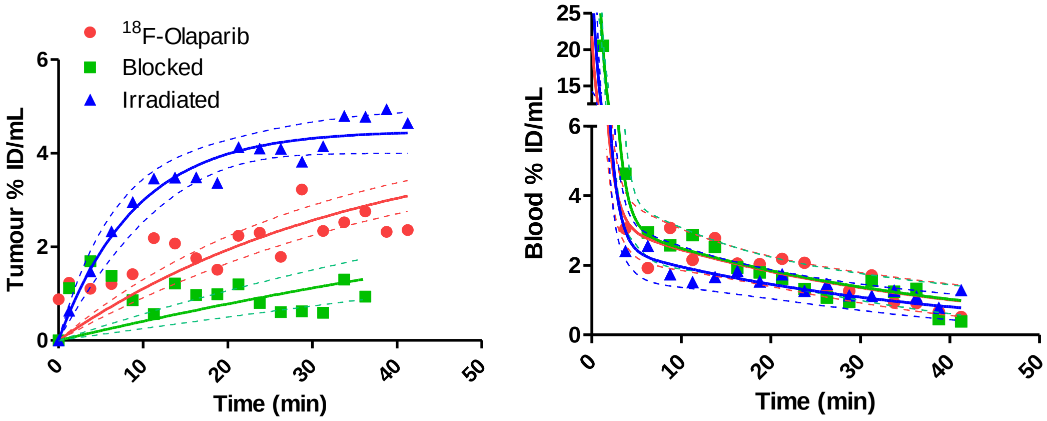
^{18}F-Olaparib
Blocked
Irradiated
Tumour % ID/mL
Time (min)
Blood % ID/mL
Time (min)

C

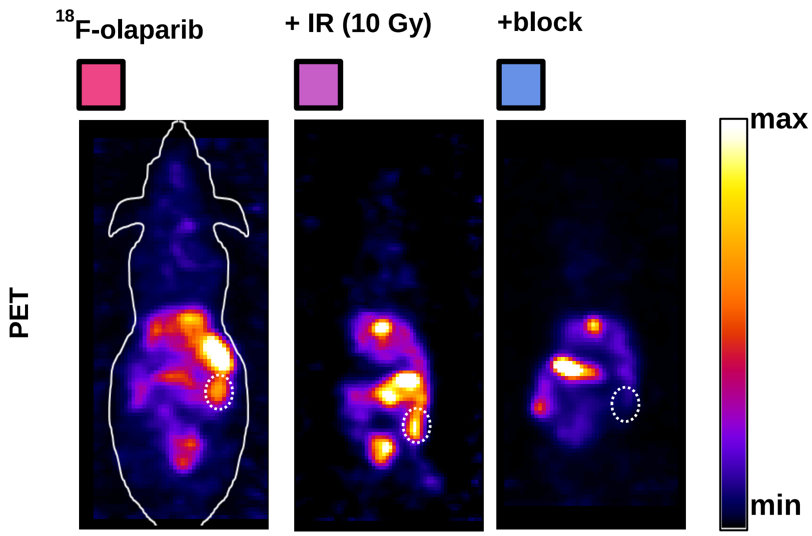

***Supplemental Fig 13**: (A) Representative dynamic PET images after intravenous bolus injection of [^{18}F]olaparib in PSN-1 tumor-bearing mice. Images are presented as coronal Maximum Intensity Projections, representing the same animals and using the same color scale as in Figure 5. (B) Image quantification of A. (C) Maximum intensity projections of the same animals, using the same color scale. Dashed circles indicate the position of the xenograft tumor.*

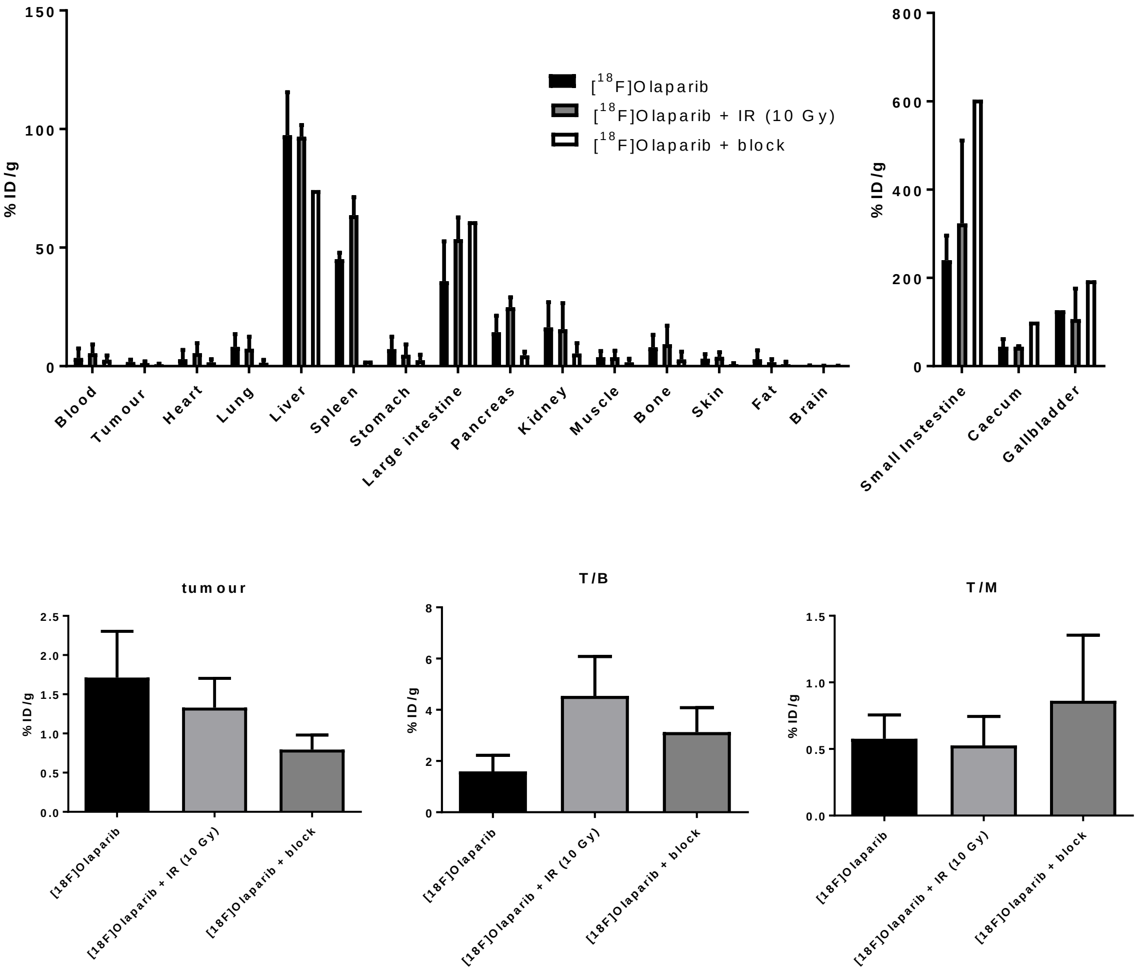

Supplemental Fig 14: *Ex vivo biodistribution of* ^{18}F-olaparib *in CaNT tumor-bearing mice, 1 h after intravenous injection.*

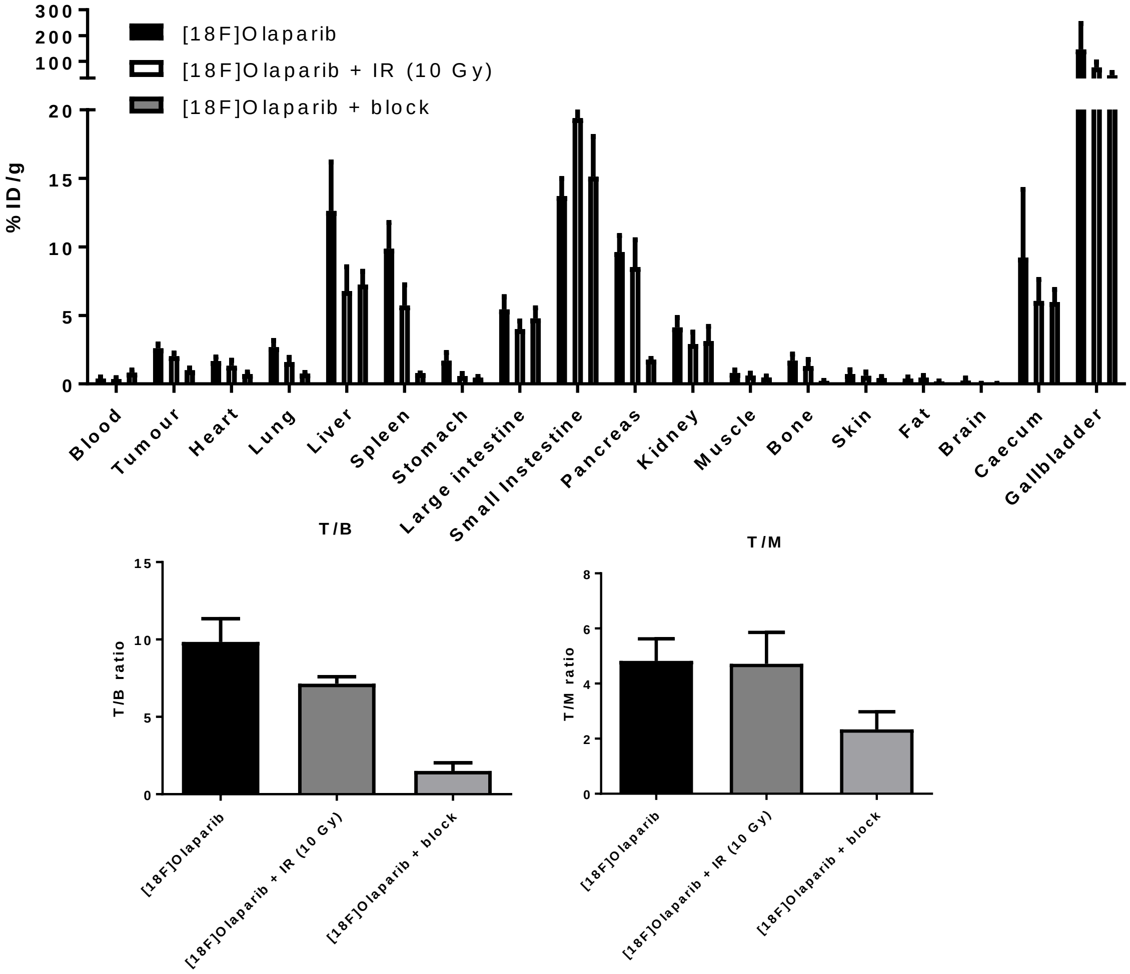

Supplemental Fig 15*: Ex vivo biodistribution of* ^{18}F-olaparib *in Capan-1 tumor-bearing mice, 1 h after intravenous injection.*

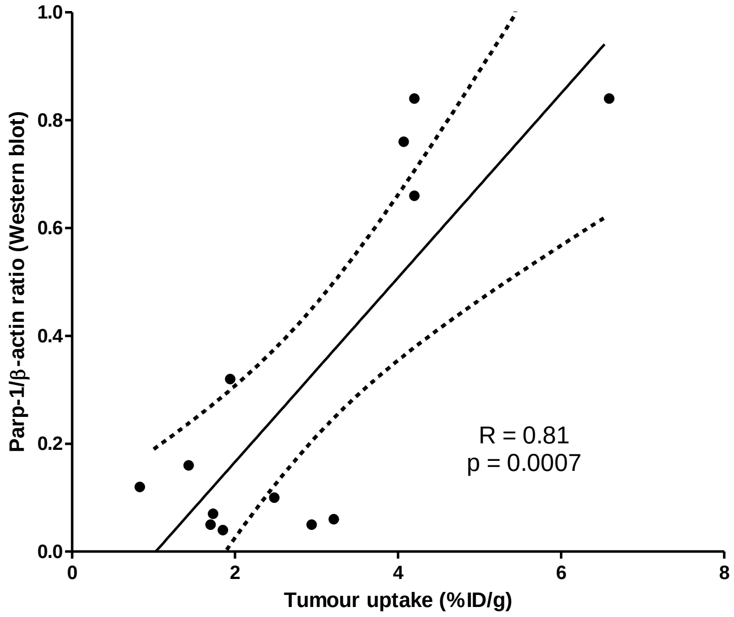

Supplemental Fig 16: *Correlation of tumor uptake determined by ex vivo biodistribution and PARP-1 expression measure via Western blot*

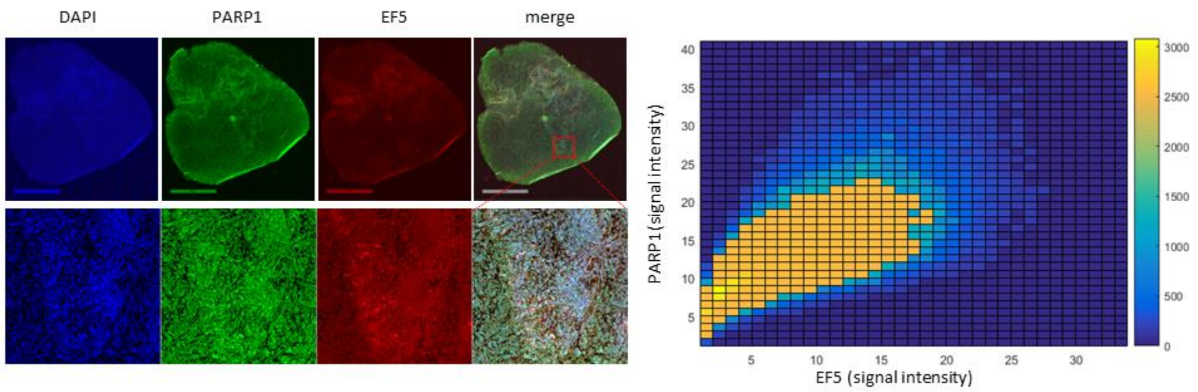

Supplemental Fig 17: *Representative section from a naive PSN-1 xenograft tumor, stained for PARP-1 and EF5, including a representative high-resolution detail, and a cytofluorogram comparing PARP-1 versus EF5 signal intensity in the high-resolution image.*

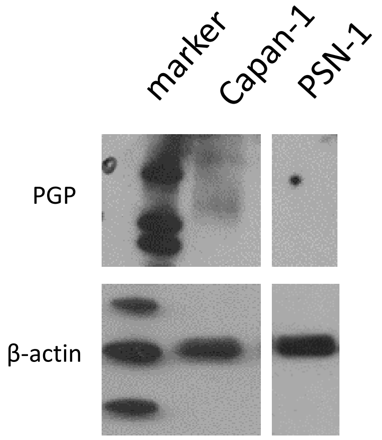

Supplemental Fig 18: *PGP pump expression in the cell lines used in this study.*

REFERENCES for supplemental material

1. Pangborn AB, Giardello MA, Grubbs RH, Rosen RK, Timmers FJ. Safe and convenient procedure for solvent purification. *Organometallics.* 1996;15:1518-1520.

2. Menear KA, Adcock C, Boulter R, et al. 4-[3-(4-cyclopropanecarbonylpiperazine-1-carbonyl)-4-fluorobenzyl]-2H-phthalazin- 1-one: a novel bioavailable inhibitor of poly(ADP-ribose) polymerase-1. *J Med Chem.* 2008;51:6581-6591.

3. Ravat P, Borozdina Y, Ito Y, Enkelmann V, Baumgarten M. Crystal Engineering of Tolane Bridged Nitronyl Nitroxide Biradicals: Candidates for Quantum Magnets. *Cryst Growth Des.* 2014;14:5840-5846.

4. Outerbridge VM, Landge SM, Tamaki H, Török B. Microwave-Promoted Solid-Acid Catalyzed One-Pot Synthesis of Phthalazinones. *Synthesis.* 2009;11:1801-1806.

5. Nezhawy AOH, Gaballah ST, Radwan MAA. Studying the reactivity of (phthalazin-1(2H)-on-2-yl)methyl trichloroacetimidate towards different C- and O-nucleophiles. *Tetrahedron Letters.* 2009;50:6646-6650.

6. Tredwell M, Preshlock SM, Taylor NJ, et al. A general copper-mediated nucleophilic 18F fluorination of arenes. *Angew Chem Int Ed Engl.* 2014;53:7751-7755.

7. Taylor NJ, Emer E, Preshlock S, et al. Derisking the Cu-Mediated ^{18}F-Fluorination of Heterocyclic Positron Emission Tomography Radioligands. *J Am Chem Soc.* 2017;139:8267-8276.

8. Cornelissen B, Hu M, McLarty K, Costantini D, Reilly RM. Cellular penetration and nuclear importation properties of 111In-labeled and 123I-labeled HIV-1 tat peptide immunoconjugates in BT-474 human breast cancer cells. *Nucl Med Biol.* 2007;34:37-46.

9. Thurber GM, Reiner T, Yang KS, Kohler RH, Weissleder R. Effect of small-molecule modification on single-cell pharmacokinetics of PARP inhibitors. *Mol Cancer Ther.* 2014;13:986-995.

10. Reiner T, Lacy J, Keliher EJ, et al. Imaging therapeutic PARP inhibition in vivo through bioorthogonally developed companion imaging agents. *Neoplasia.* 2012;14:169-177.

11. Kersemans V, Cornelissen B, Hueting R, et al. Hypoxia imaging using PET and SPECT: the effects of anesthetic and carrier gas on [Cu]-ATSM, [Tc]-HL91 and [F]-FMISO tumor hypoxia accumulation. *PLoS One.* 2011;6:e25911.

12. Koch CJ. http://www.hypoxia-imaging.org/v2/methods/ef5manual.htm. Accessed 21/01/2011, 2011.

13. Lee J, Siemann DW, Koch CJ, Lord EM. Direct relationship between radiobiological hypoxia in tumors and monoclonal antibody detection of EF5 cellular adducts. *Int J Cancer.* 1996;67:372-378.

14. Hueting R, Kersemans V, Cornelissen B, et al. A comparison of the behavior of ^{64}Cu-acetate and ^{64}Cu-ATSM in vitro and in vivo. *J Nucl Med.* 2014;55:128-134.
